# NUPR1, a new target in liver cancer: implication in controlling cell growth, migration, invasion and sorafenib resistance

**DOI:** 10.1038/cddis.2016.175

**Published:** 2016-06-23

**Authors:** M R Emma, J L Iovanna, D Bachvarov, R Puleio, G R Loria, G Augello, S Candido, M Libra, A Gulino, V Cancila, J A McCubrey, G Montalto, M Cervello

**Affiliations:** 1 Institute of Biomedicine and Molecular Immunology “Alberto Monroy”, National Research Council (CNR), Palermo, Italy; 2 Biomedic Department of Internal Medicine and Specialties (DiBiMIS), University of Palermo, Palermo, Italy; 3 INSERM UMR1068, Center of Research in Cancerology of Marseille (CRCM), Marseille, France; 4 Cancer Research Centre, Hôpital L'Hotel-Dieu de Québec, Centre Hospitalier Universitaire de Québec, Quebec City (Quebec), Canada; 5 Department of Molecular Medicine, Faculty of Medicine, Laval University, Quebec City (Quebec), Canada; 6 Istituto Zooprofilattico Sperimentale della Sicilia “A. Mirri”, Histopathology and Immunohistochemistry Laboratory, Palermo, Italy; 7 Department of Biomedical and Biotechnological Sciences, University of Catania, Catania, Italy; 8 Tumor Immunology Unit, Department of Health Science, University of Palermo, Palermo, Italy; 9 Department of Microbiology and Immunology, Brody School of Medicine at East Carolina University, Greenville, NC, USA

## Abstract

Sorafenib, an oral multikinase inhibitor, is the only approved agent for the treatment of advanced hepatocellular carcinoma (HCC). However, its benefits are modest, and as its mechanisms of action remain elusive, a better understanding of its anticancer effects is needed. Based on our previous study results, we investigated here the implication of the nuclear protein 1 (NUPR1) in HCC and its role in sorafenib treatment. NUPR1 is a stress-inducible protein that is overexpressed in various malignancies, but its role in HCC is not yet fully understood. We found that NUPR1 expression was significantly higher in primary human HCC samples than in the normal liver. Knockdown of *NUPR1* significantly increased cell sensitivity to sorafenib and inhibited the cell growth, migration and invasion of HCC cells, both *in vitro* and *in vivo*. Moreover, *NUPR1* silencing influenced the expression of *RELB* and *IER3* genes. Unsurprisingly, *RELB* and *IER3* knockdown also inhibited HCC cell viability, growth and migration. Using gene expression profiling of HCC cells following stable *NUPR1* knockdown, we found that genes functionally involved in cell death and survival, cellular response to therapies, lipid metabolism, cell growth and proliferation, molecular transport and cellular movement were mostly suppressed. Network analysis of dynamic gene expression identified NF-*κ*B and ERK as downregulated gene nodes, and several HCC-related oncogenes were also suppressed. We identified *Runt-related transcription factor 2* (*RUNX2*) gene as a *NUPR1*-regulated gene and demonstrated that *RUNX2* gene silencing inhibits HCC cell viability, growth, migration and increased cell sensitivity to sorafenib. We propose that the NUPR1/RELB/IER3/RUNX2 pathway has a pivotal role in hepatocarcinogenesis. The identification of the NUPR1/RELB/IER3/RUNX2 pathway as a potential therapeutic target may contribute to the development of new treatment strategies for HCC management.

Hepatocellular carcinoma (HCC) is the most common liver cancer, accounting for 90% of primary liver cancers and is currently the third major cause of cancer-related deaths globally.^1^ Although recent progress in diagnostic and treatment technologies has improved survival, the long-term survival of HCC patients remains dismal owing to the lack of adequate therapies. Despite the approval of sorafenib (Nexavar, BAY43-9006), an oral multi-kinase inhibitor that targets Raf kinases, and several other tyrosine kinases, including vascular endothelial growth factor receptor-2/3, platelet-derived growth factor receptor-*β*, Fms-like tyrosine kinase 3 and c-Kit, for the treatment of advanced HCC,^1^ and its extensive application in clinical practice during the past few years, it is increasingly clear that the benefits of sorafenib are modest, and the precise mechanism of action of this drug remains elusive. Indeed, several targets other than Raf and receptor tyrosine kinases have been identified,^2, 3^ including signal transducer and activator of transcription 3 signaling,^4^ and the secretory pathway.^5^ The mitochondria is also a sorafenib target, as shown by the inhibition of mitochondrial respiration in liver cancer cells and a shift toward aerobic glycolysis and glycolytic adaptation response to mitochondrial damage during sorafenib treatment.^6, 7^

Several reports have indicated that sorafenib may induce cell death signaling pathways via endoplasmic reticulum (ER) stress activation.^8, 9, 10^ Our recent studies analyzing the molecular mechanisms of sorafenib treatment in human HCC cells identified several genes involved in ER stress response modulated by sorafenib.^11^ In particular, the stress-inducible gene *nuclear protein-1* (*NUPR1*, also known as *p8/Com-1*) was upregulated after sorafenib treatment and its expression was found to be additionally potentiated on treatment with the anti-inflammatory drug celecoxib.^11, 12^

The *NUPR1* gene was initially identified as a transcriptional factor, which is rapidly and strongly induced in rat pancreatic acinar cells during acute pancreatitis, developing pancreas and pancreatic regeneration.^13^ Recently, NUPR1, a small highly basic and loosely folded stress-inducible multifunctional protein, has emerged as a new drug-targetable protein whose blockade could prevent cancer progression and metastasis development.^14^ The functions of NUPR1 in the various tissues with different molecular contexts may be different or even opposite, therefore it can be seen as a double-edged knife with its ability to promote both tumor suppression and tumor development.^14, 15, 16, 17^ It is also implicated in drug resistance mechanisms in pancreatic and breast cancer models.^18, 19^ In particular, in the pancreatic model some types of stress increased the expression of *NUPR1* and of three of its target genes, activating transcription factor 4 (*ATF4*), C/EBP-homologous protein (*CHOP*) and Tribbles homolog 3 (*TRB3*), acting in the ER stress pathway and inducing cell death.^15^ On the other hand, subsequent studies conducted with the same tumor model showed that NUPR1 can induce the expression of pro-survival genes, such as V-rel avian reticuloendotheliosis viral oncogene homolog B (*RELB*) and immediate early response 3 (*IER3*).^16^

In the liver, NUPR1 has been shown to be an important element in hepatocyte stress response after hepatic injury by CCl_4_.^20^ NUPR1 has also been identified as a key regulator and metabolic switch in response to mitochondrial damage during liver cancer progression.^21^ In addition, Bak *et al.*^22^ recently reported that *NUPR1* is activated by hepatitis B virus X protein and mediates the cell growth and survival of HBV-positive cells.

As the role of NUPR1 in hepatocarcinogenesis is not yet fully understood, we decided to examine its involvement in the context of sorafenib treatment in HCC cells.

## Results

### Sorafenib treatment increases NUPR1 expression levels

**Figure 1:**
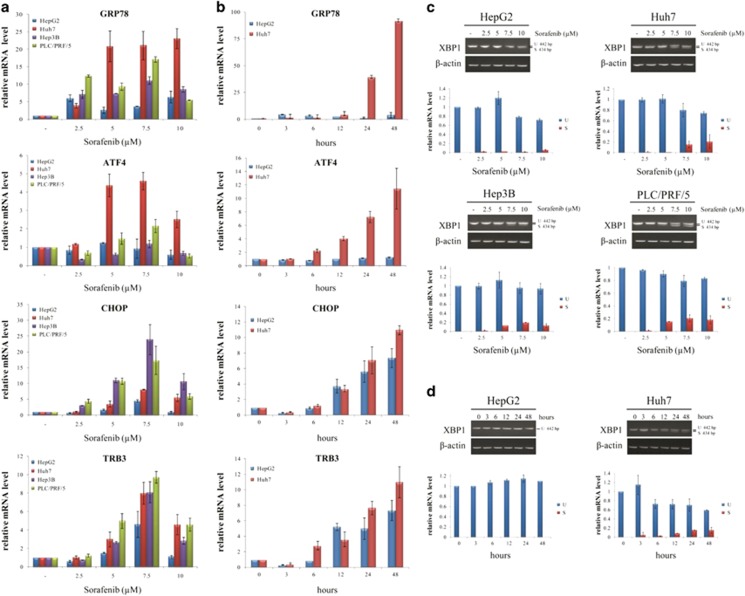
Expression of ER stress genes after sorafenib treatment in HCC cells. (**a** and **c**) Dose- and (**b** and **d**) time-dependent effects of sorafenib treatment on ER stress gene expression in HCC cell lines determined by qPCR (**a** and **b**) and semiquantitative-PCR (**c** and **d**). In panels (**a** and **c**), HCC cells were treated with the indicated concentrations of sorafenib, and total RNA was extracted after 24 h of treatment. In panels (**b** and **d**), HepG2 and Huh7 cells were treated with 7.5 *μ*M sorafenib, and total RNA was extracted at different times of treatment. U=unspliced XBP1 mRNA; S=spliced XBP1 mRNA. In panels (**a** and **b**), relative expression was calculated as the ratio of drug-treated samples *versus* control (DMSO) and corrected by the quantified expression level of *β*-actin. The results shown are the means±S.D. of three experiments, each performed in triplicate

NUPR1 is a stress-inducible protein and, as mentioned before, one possible mechanism of action of sorafenib is induction of ER stress response.^8, 9, 10^ Therefore, we first investigated the effects of sorafenib on well-known ER stress-regulated genes. Sorafenib treatment activated ER stress response in HCC cell lines in a dose- and time-dependent manner (Figure 1). Genes involved in ER stress response, such as *GRP78*, *ATF4*, *CHOP* and *TRB3* were upregulated (Figures 1a and b), and splicing of X-box-binding protein 1 (XBP1) mRNA was also induced (Figures 1c and d).

**Figure 2:**
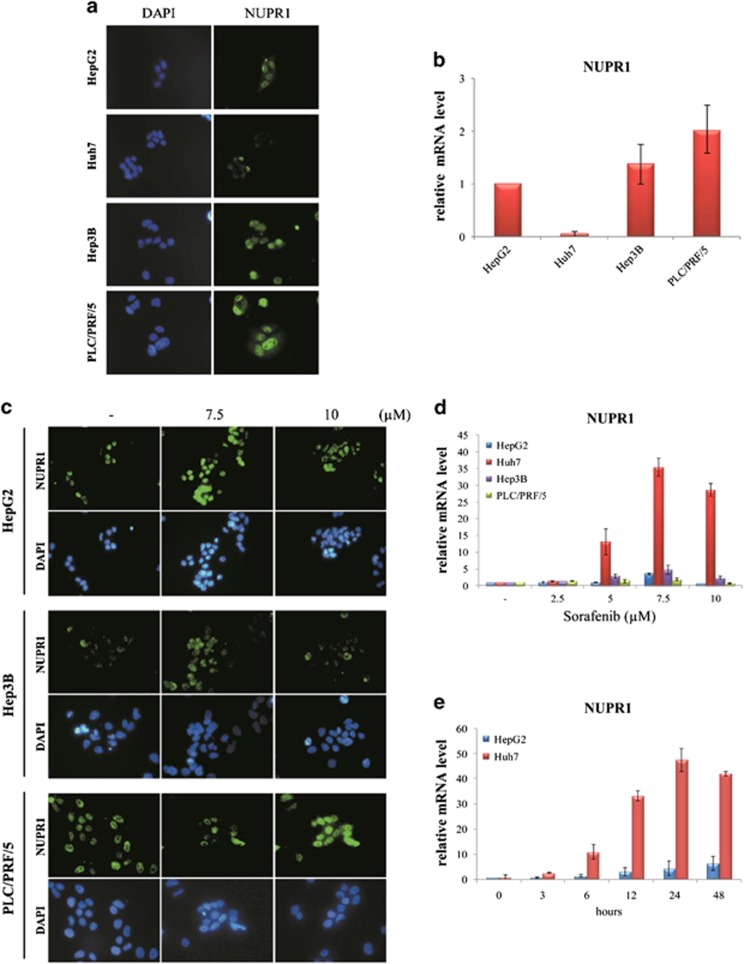
Expression of NUPR1 in HCC cells. (**a**) NUPR1 protein and (**b**) mRNA expression in HCC cells in basal condition. (**c**) Immunofluorescence analysis of NUPR1 protein expression after treatment for 3 h with the indicated concentrations of sorafenib in HCC cells. (**d**) HCC cells were treated with the indicated concentrations of sorafenib, and total RNA was extracted after 24 h of treatment. (**e**) HepG2 and Huh7 cells were treated with 7.5 *μ*M sorafenib, and total RNA was extracted at different times of treatment

We then analyzed the basal expression of NUPR1 on the human HCC cell lines HepG2, Huh7, Hep3B and PLC/PRF/5, both at the protein and mRNA levels. HCC cell lines showed different expression levels of NUPR1 (Figures 2a and b). PLC/PRF/5 cells expressed the highest levels while Huh7 cells showed the lowest expression of both protein (Figure 2a) and mRNA (Figure 2b).

Subsequently, NUPR1 protein expression in HCC cells was investigated by immunofluorescence analysis after sorafenib treatment. As shown in Figure 2c, NUPR1 was localized in the nuclei of HCC cells, and its expression increased after sorafenib treatment. NUPR1 mRNA expression was similarly induced in HCC cell lines in a dose- and time-dependent manner upon sorafenib treatment (Figures 2d and e).

### Bioinformatics analysis of NUPR1 gene expression in liver cancer

To explore the clinical relevance of NUPR1 in liver cancer development, we analyzed the HCC microarray data sets available on the Oncomine software. Only six out of the nine data sets showed a statistical significance <0.05 (by Student's *t*-test) when expression levels were analyzed in the normal liver (NL) tissues, precancerous lesions and HCC samples (Supplementary Table S1) and were then considered for further analyses.^23, 24, 25, 26, 27, 28, 29, 30^ The data summarized in Supplementary Table S2 show that NUPR1 transcript levels were significantly lower in cirrhosis and liver cell dysplasia than in NL tissue, while higher *NUPR1* gene expression levels were detected in HCC samples than in normal and cancer precursor tissues. An opposite trend was observed in the Archer *et al.*^23^ data set. Interestingly, HCC harboring *TP53* mutations showed higher *NUPR1* transcript levels than in the HCC wild-type subset, and *NUPR1* gene expression levels were higher in early-stage than in advanced-stage HCC samples (Supplementary Table S2).

### Expression of NUPR1 in human HCC tissues

**Figure 3:**
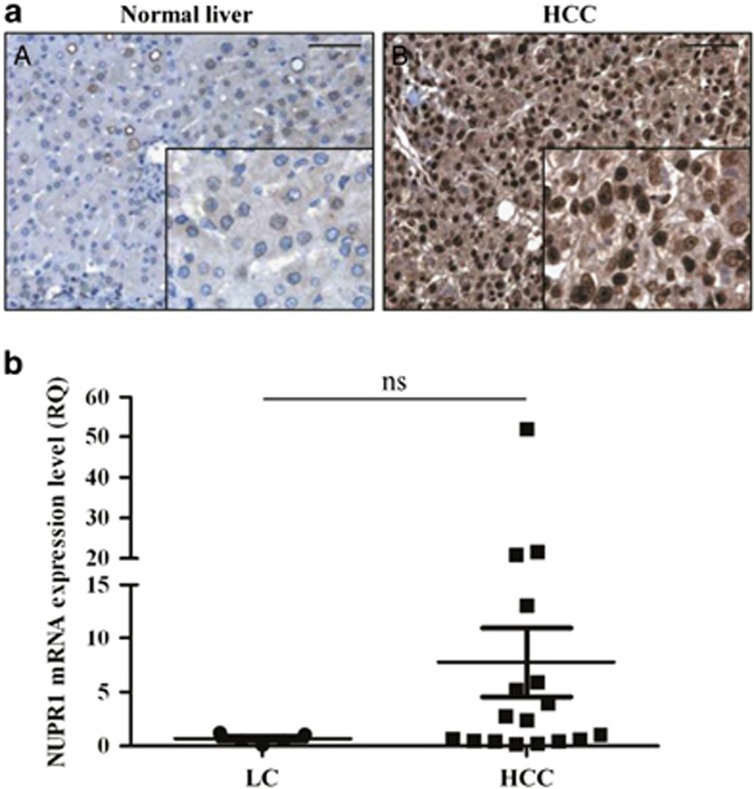
NUPR1 expression in human HCC samples. (**a**) NUPR1 protein expression levels were examined by immunohistochemistry in the NL (**A**) and HCC (**B**) tissues. Magnification= × 20, insert magnification= × 40. Scale bar=100 *μ*m. (**b**) *NUPR1* gene expression analysis in 17 HCC tissues and 5 surrounding non-tumor cirrhotic tissues (LC) performed by qPCR. Data are indicated as *NUPR1* fold change (relative quantitation, RQ) compared with control (RQ=1 calculated as the mean of NUPR1 Ct in NL tissues). Data are expressed as mean±S.D. NS=non-significant

In view of the results above, we investigated NUPR1 expression in HCC (*n*=21), in liver cirrhosis (*n*=3) and in NL tissues (*n*=3) by immunohistochemistry (Figure 3a) and by quantitative-PCR (qPCR) in 17 patients with liver cirrhosis-associated HCC, in 5 surrounding non-tumor regions (cirrhotic tissues) and in 4 NL tissues (Figure 3b). Immunohistochemical analysis showed high NUPR1 expression in all the HCC patients studied; moreover, NUPR1 displayed a nuclear localization in the tumor hepatocytes but not in NL hepatocytes (Figure 3a). The sum of the scores (intensity+percentage of positive nuclei) for NUPR1 was significantly higher in tumors (*P*<0.05) than in NL tissues (Supplementary Table S3A). In addition, the percentage of NUPR1-positive nuclei was significantly different in HCC tissues with different differentiation grades (*P*<0.05), whereas no correlation was observed between NUPR1 expression and TNM classification (Supplementary Table S3B).

In 9 of the 17 (53%) patients analyzed, NUPR1 mRNA expression was higher in the tumor samples than in NL tissues (Figure 3b). Of note, both HCV-associated (7 out of 14) and HBV-associated (1 out of 3) HCC showed high NUPR1 expression levels. In the cirrhotic tissues, three out of the five patients displayed a lower *NUPR1* expression than in the NL. These results are therefore in agreement with the bioinformatics analyses. However, possibly owing to the limited number of patients in both cases results did not reach statistical significance (NL *versus* LC *P*=0.7600; NL *versus* HCC *P*=0.6715; LC *versus* HCC *P*=0.1829).

### Small interference RNA (siRNA)-mediated NUPR1 gene silencing inhibits cell growth and increases cell response to sorafenib treatment

**Figure 4:**
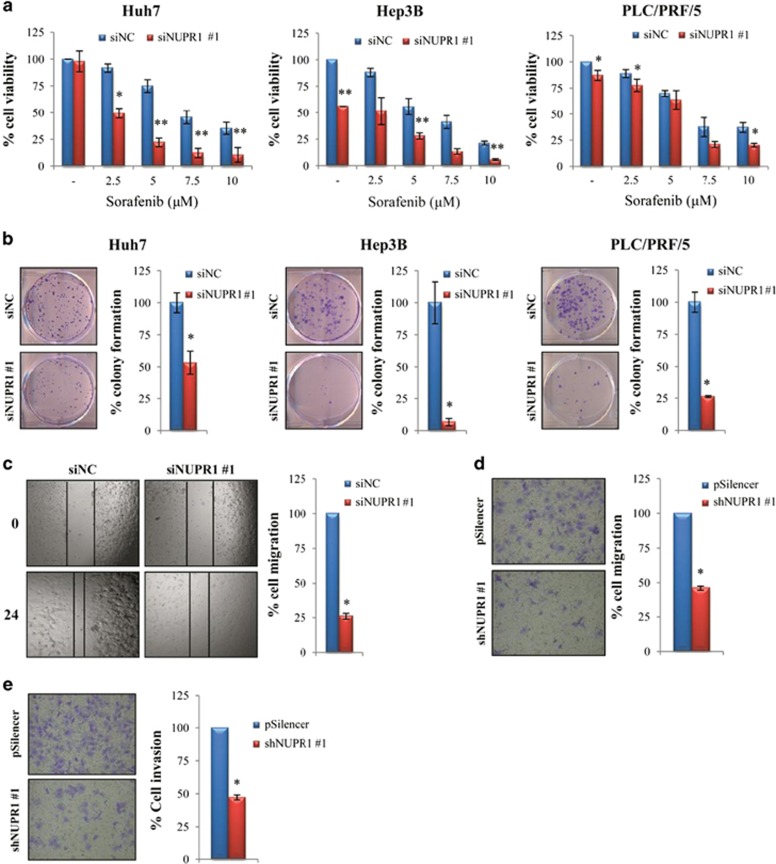
NUPR1 regulates cell viability, growth, migration and invasion of HCC cells. (**a**) Cell viability of HCC cells transfected with siNUPR1 (siNUPR1 #1) and siNC was assessed by MTS assay after treatment with the indicated concentrations of sorafenib for 48 h. Data are expressed as the percentage of control cells and are the means±S.D. of three separate experiments, each performed in triplicate. *P<0.05, **P<0.01. (**b**) Representative images of clonogenic assay of HCC cells transfected with siNUPR1 (siNUPR1 #1) and siNC. The experiment continued for 14 days. Surviving colonies were stained and counted. Data are expressed as the percentage of colonies and are the means±S.D. of three separate experiments, each performed in duplicate. (**c**) Representative images of wound-healing assay after *NUPR1* siRNA-mediated gene silencing (shNUPR1 #1) in PLC/PRF/5. The experiment was conducted for 24 h. Data are reported as the percentage of cell migration and represent the average±S.D. of three experiments, each performed in duplicate. *P<0.05. (**d**) Representative images of transwell migration assay of Hep3B shNUPR1 (shNUPR1 #1) or Hep3B pSilencer cells. Data are reported as the percentage of migrated cells compared with control (siNC) and are the means±S.D. of three separate experiments, each performed in duplicate (*P<0.05). (**e**) Matrigel invasion assay in Hep3B shNUPR1 cells (shNUPR #1) compared with pSilencer as control. Data are reported as the percentage of invaded cells compared with control (pSilencer) and are the means±S.D. of three separate experiments, each performed in duplicate. *P<0.05

To investigate the functional role of *NUPR1* in the regulation of tumor cell growth and chemoresistance, HCC cells were transfected with specific human *NUPR1* siRNA and the effects on cell viability and chemoresistance were evaluated after sorafenib treatment. We found that *NUPR1* knockdown decreased cell viability and colony formation and increased tumor cell sensitivity to sorafenib treatment (Figures 4a and b and Supplementary Figures S1A and B). These data demonstrate that *NUPR1* may have an oncogenic potential in HCC and it may be involved in drug resistance. This was further confirmed after generation of *NUPR1* knockdown stable clones using *NUPR1*-specific short hairpin RNA (shRNA), in which *NUPR1* gene silencing significantly decreased Hep3B cell viability and proliferation (Supplementary Figures S1C and D) and increased sensitivity to sorafenib treatment (Supplementary Figure S1C).

### Downregulation of NUPR1 decreases HCC cell migration and invasion

Next we assessed whether downregulation of *NUPR1* could affect HCC cell migration and invasiveness. We found that *NUPR1* gene silencing significantly reduced PLC/PRF/5 cell migration in wound-healing assays (Figure 4c and Supplementary Figure S1E). Transwell migration and Matrigel invasion assays confirmed these findings, as knockdown of *NUPR1* by shRNA significantly reduced Hep3B cell migration and invasion (Figures 4d and e and Supplementary Figure S1F). These results are in agreement with data reported by Lee *et al.*^21^ and indicate that NUPR1 has a role in regulating the metastatic potential of human HCC cells.

### NUPR1 regulates RELB and IER3 gene expression

**Figure 5:**
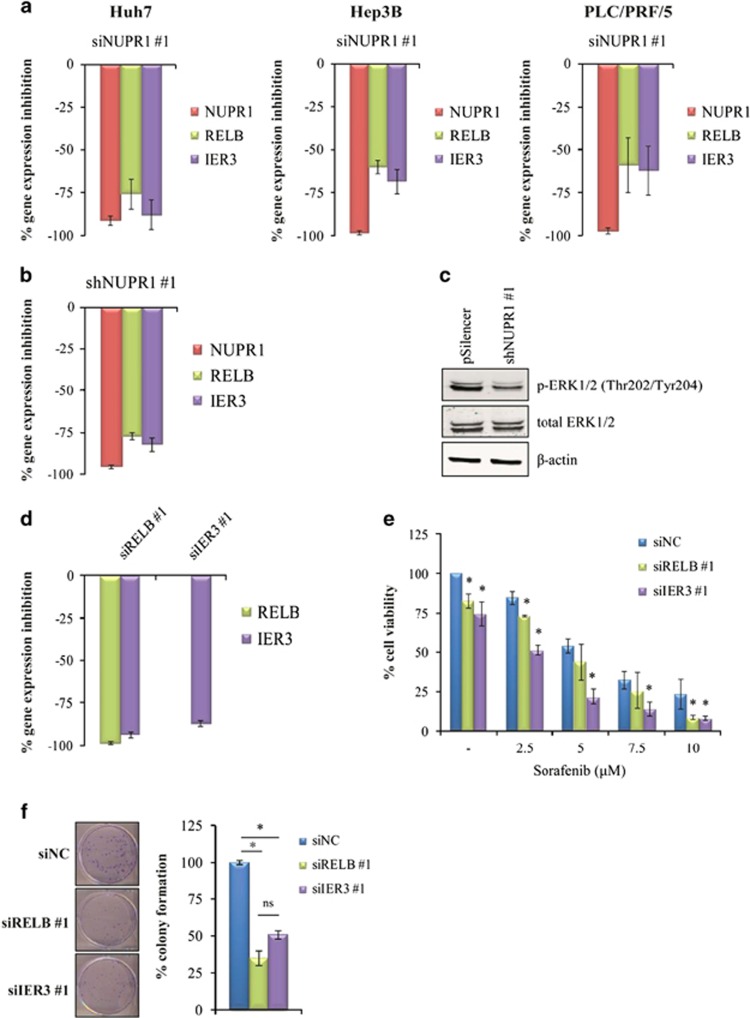
NUPR1 regulates expression of *RELB* and *IER3* genes, and RELB and IER3 regulate cell viability and the colony-formation capacity of HCC cells. (**a**) Gene expression analysis by qPCR in HCC cells after *NUPR1* gene silencing (siNUPR1 #1). Data are reported as the percentage of gene expression inhibition of each gene and are the means±S.D. of three separate experiments, each performed in triplicate. (**b**) Gene expression analysis, by qPCR, in Hep3B shNUPR1 (shNUPR1 #1) cells compared with pSilencer, as control. Data are expressed as reported in panel (**a**). (**c**) Western blotting analysis of *P*-ERK1/2 (Thr202/Tyr204) and total ERK1/2 in Hep3B shNUPR1 (shNUPR #1) and in control cells (pSilencer). (**d**) Gene expression analyses by qPCR after *RELB* and *IER3* gene silencing in Hep3B cells. Data are expressed as reported in panel (**a**). (**e**) Cell viability of Hep3B cells transfected with siRELB, siIER3 and siNC was assessed by MTS assay after treatment with the indicated sorafenib concentrations for 48 h. Data are expressed as reported in Figure 4. *P<0.05. (**f**) Representative images of the clonogenic assay of Hep3B cells transfected with siRELB, siIER3 and siNC. The experiment continued for 14 days. Surviving colonies were stained and counted. Data are expressed as reported in Figure 4. *P<0.05; ns=non-significant

To further explore the functional role of *NUPR1* in HCC, we focused on *NUPR1*-regulated genes, *RELB* and *IER3*.^16^ *NUPR1* gene silencing caused downregulation of both RELB and IER3 mRNAs in HCC cell lines (Figure 5a and Supplementary Figure S2A). Gene expression analyses using the stable Hep3B shNUPR1 knockdown clones confirmed that *NUPR1* silencing caused a significant downregulation of *RELB* and *IER3* genes (Figure 5b and Supplementary Figure S2B).

IER3 has been shown to induce sustained activation of extracellular signal-regulated kinase (ERK) by inhibition of the serine–threonine protein phosphatases-2A (PP2A) activity.^31, 32^ To verify whether *NUPR1* gene silencing may also influence ERK1/2 phosphorylation, western blotting analysis was performed in stable Hep3B shNUPR1 clones. As shown in Figure 5c and Supplementary Figure S2C, there was a sharp decrease in ERK1/2 phosphorylation (Thr202/Tyr204) status in Hep3B shNUPR1 cells compared with control cells. These data suggest that NUPR1 may act through the RELB/IER3 pathway by regulating the activation of ERK kinase, which is involved in a pro-survival signaling pathway, and could be responsible for the improved sorafenib effects on cell viability observed in previous experiments.

### Silencing of RELB and IER3 by siRNA decreases HCC cell growth and chemoresistance

We have shown that *NUPR1* gene knockdown in HCC cells produces a significant downregulation of the pro-survival genes *RELB* and *IER3*. Specific siRNA was therefore used for *RELB* and *IER3* silencing in Hep3B cells (Figure 5d and Supplementary Figure S2D), which led to a significant decrease in cell viability in basal conditions (Figure 5e and Supplementary Figure S2E, no treatment), and also significantly diminished clonogenic capacity (Figure 5f) and significantly increased cell sensitivity to sorafenib treatment (Figure 5e and Supplementary Figure S2E). As expected, *IER3* expression was also downregulated after *RELB* silencing (Figure 5d and Supplementary Figure S2D), confirming that *IER3* is a downstream *RELB*-regulated gene in HCC cells. These results confirm the existence of a NUPR1/RELB/IER3 pathway involved in the regulation of HCC cell growth and drug resistance.

### NUPR1 gene silencing inhibits tumor growth *in vivo*

**Figure 6:**
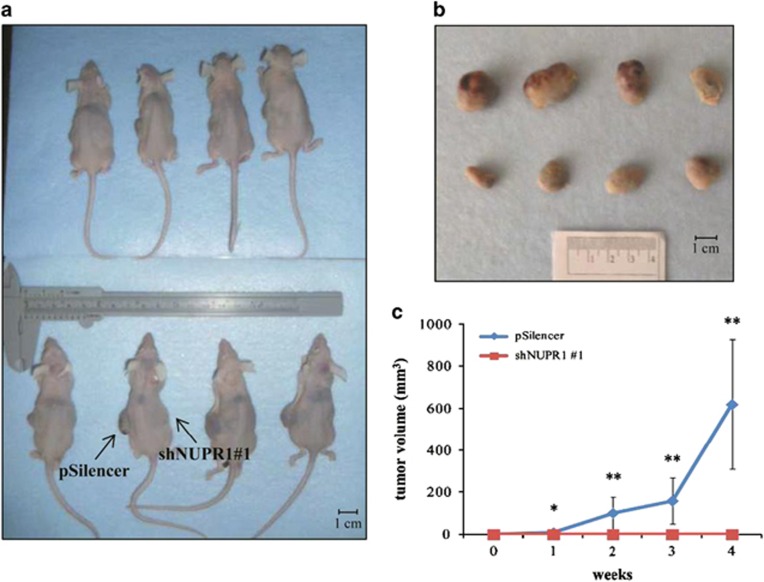
*NUPR1* knockdown inhibited tumor growth of Hep3B cells in nude mice. (**a**) Microphotography of 8 out of the 12 mice inoculated with stable Hep3B cells harboring *NUPR1* shRNA (shNUPR1) (right flank) or non-specific shRNA (pSilencer) (left flank). (**b**) Microphotographs of tumors collected after 4 weeks of injection with Hpe3B pSilencer cells. (**c**) Tumor growth of Hep3B cells harboring pSilencer or shNUPR1. *P<0.05; **P<0.01

To assess the effect of *NUPR1* gene silencing on tumorigenicity in HCC cells, Hep3B shNUPR1 cells were injected into nude mice. Four weeks after injection, we found that Hep3B pSilencer cells gave rise to tumor in all cases (*n*=12), while Hep3B shNUPR1 cells inoculated into the same animals were unable to form tumors (Figures 6a–c). These data demonstrate that NUPR1 is essential for tumor cell establishment and growth *in vivo*.

### Transcriptomic analysis identifies gene expression changes following NUPR1 knockdown

**Figure 7:**
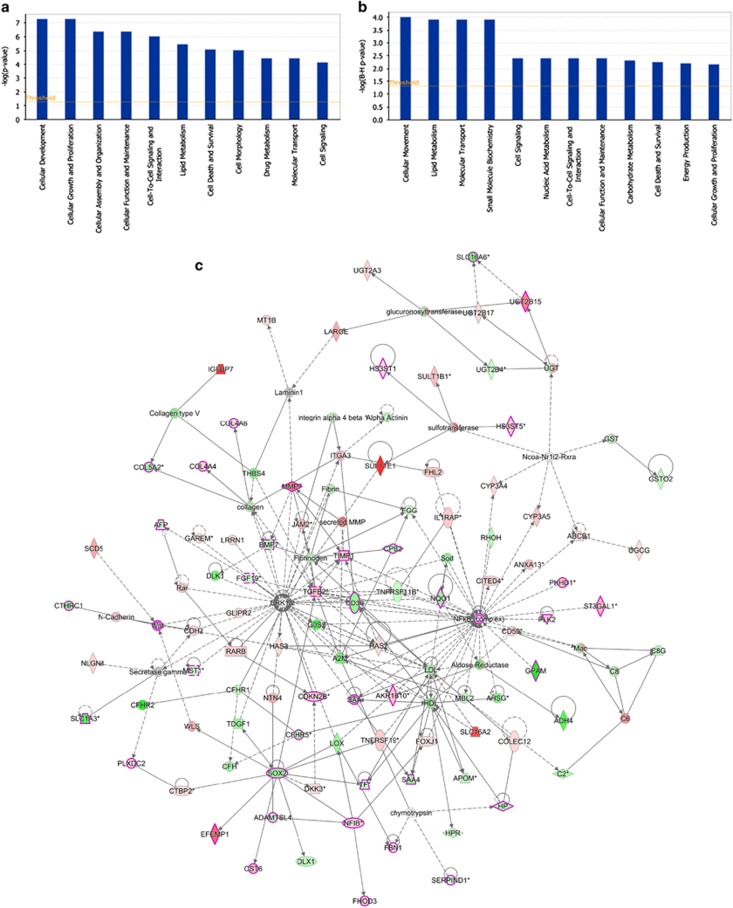
Functional analysis for the data set of differentially expressed genes and network analysis of dynamic gene expression obtained following shRNA-mediated *NUPR*1 knockdown in Hep3B cells. (**a** and **b**) IPA functional pathway analyses of genes differentially expressed (≥ 3-fold) in Hep3B cells upon *NUPR1* suppression. Top functions that meet a *P*-value cutoff of 0.05 are displayed. The orange line represents the cutoff value for significance. (**a**) Genes that were upregulated and (**b**) genes downregulated. (**c**) The five top-scoring networks were merged and are displayed graphically as nodes (genes/gene products) and edges (the biological relationships between the nodes). Intensity of the node color indicates the degree of up regulation (red) or downregulation (green). Nodes are displayed using various shapes that represent the functional class of the gene product (rhomboid=transporter; square=cytokine; diamond=enzyme; vertical oval=transmembrane receptor; horizontal oval=transcription factor; rectangle=nuclear receptor; hexagon=translation factor; circle=other). Edges are displayed with various labels that describe the nature of the relationship between the nodes:→acts on; -— binding only. The length of an edge reflects the evidence supporting the specific node-to-node relationship, as edges supported by articles from the literature are shorter. Dotted edges represent indirect interaction

To better understand the molecular mechanism(s) of NUPR1 action in HCC cells, we compared the global gene expression of the Hep3B shNUPR1 clone against the corresponding control clone (pSilencer). For each comparison, a subset of common differentially expressed genes was selected by initial filtering on confidence at *P*-value ≤0.05, followed by filtering on expression level (≥ 3 fold). Using these stringent selection criteria, we found 273 genes upregulated and 153 genes downregulated in Hep3B cells following *NUPR1* knockdown (Supplementary Table S4). Pathway and network analyses generated using the Ingenuity Pathway Analysis (IPA) software showed the major functionally related gene groups, which were differentially expressed in the shNUPR1 clone compared with control cells (Supplementary Table S5 and Figure 7). Pathways implicated in cellular development, cell growth and proliferation and cell assembly and organization, as well as cellular function and maintenance were mostly upregulated in the stable Hep3B shNUPR1 knockdown clone (Figure 7a), whereas pathways implicated in cellular movement, lipid metabolism, molecular transport and cellular response to therapies were mostly suppressed (Figure 7b).

Common networks, generated by merging the five top-scoring networks, including both downregulated and upregulated genes (≥ 3-fold), recognized some functionally related gene nodes upon *NUPR1* knockdown, such as nuclear factor kappa from B cells (NF-κB), ERK, p38MAPK and transforming growth factor *β*2 (TGF*β*2) (Figure 7c). In addition, several genes known to be involved in hepatocarcinogenesis were also suppressed, such as *fibroblast growth factor 19* (*FGF19*), *Dickkopf 3* (*DKK3*), *cyclin-dependent kinase inhibitor 2B* (*CDKN2B*) and *TGFβ2*.

To validate the microarray results, we arbitrarily selected seven differentially expressed genes and quantified their expression by semiquantitative PCR or qPCR. We found that in all cases both methods (microarray analysis and PCR) detected similar patterns for selected genes (Supplementary Table S6).

**Figure 8:**
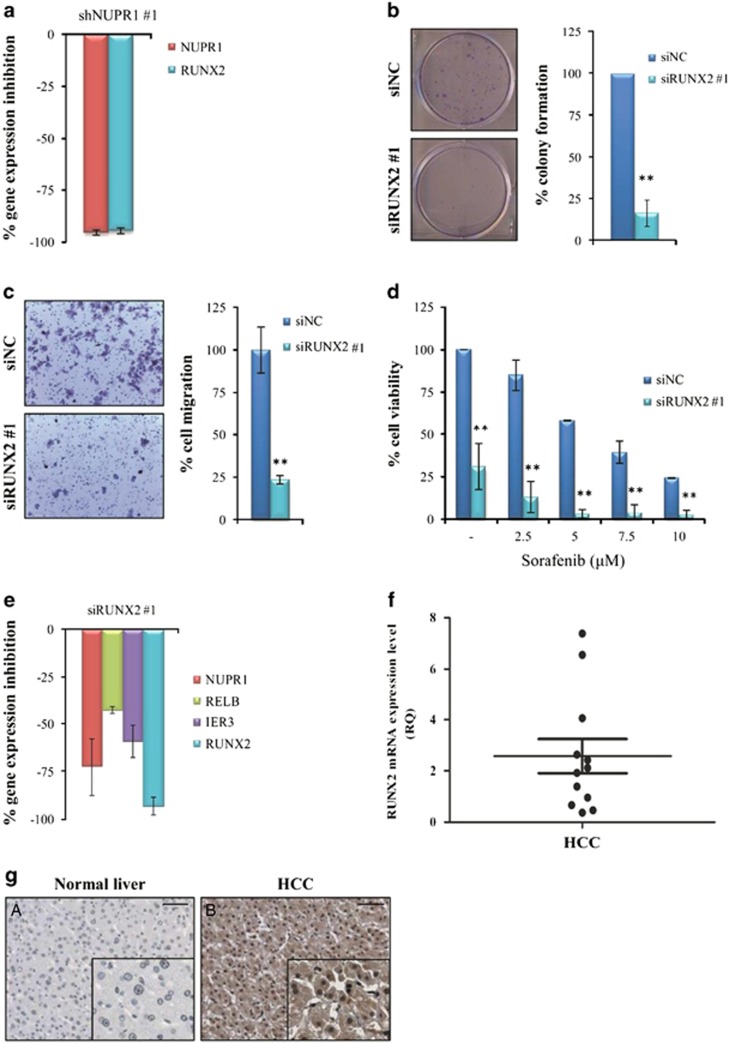
RUNX2 regulates cell viability, growth, migration and the expression of *NUPR1*, *RELB* and *IER3* genes in Hep3B cells and is expressed in human HCC samples. (**a**) Gene expression analysis by qPCR after *NUPR1* gene silencing (shNUPR1 #1) in Hep3B cells. (**b**) Colony assay after *RUNX2* siRNA-mediated gene knockdown. Data are expressed as reported in Figure 4. (**c**) Representative images of transwell migration assay after *RUNX2* gene silencing in Hep3B cells. Data are expressed as reported in Figure 4.**P<0.01. (**d**) Cell viability of Hep3B cells transfected with siRUNX2 (siRUNX2 #1) and siNC was assessed by MTS assay after treatment with the indicated concentrations of sorafenib for 48 h. Data are expressed as reported in Figure 4. (**e**) Gene expression analysis after *RUNX2* gene silencing (siRUNX2 #1) in Hep3B cells performed by qPCR. Data are expressed as reported in Figure 5. (**f**) *RUNX2* gene expression analysis in 12 HCC tissues performed by qPCR. Data are indicated as reported in Figure 3. (**g**) RUNX2 protein expression levels were examined by immunohistochemistry in the NL (**A**) and HCC tissues (**B**). Magnification= × 20, insert magnification= × 40. Scale bar=100 *μ*m

The observation that bone morphogenetic protein 7 (*BMP7*), a member of the TGF*β* superfamily, is downregulated in Hep3B cells upon *NUPR1* knockdown appears interesting, as *BMP7* has been demonstrated to be overexpressed in HCC and may be a potential biomarker for poor prognosis in HCC patients.^33^ Several reports have demonstrated that BMP7 transcriptionally induces Runt-related transcription factor 2 (*RUNX2*) expression.^34, 35^ Intriguingly, Wang *et al.*^36^ reported that in ovarian cancer cells *RUNX2* gene silencing provides a sharp downregulation of *NUPR1*, suggesting the existence of an interaction between these two transcription factors. Indeed, in our preliminary microarray analyses conducted using ≥1.5-fold selection criteria, we found that *RUNX2* was downregulated in Hep3B cells upon *NUPR1* suppression. Inhibition of RUNX2 mRNA expression upon *NUPR1* silencing was further confirmed by qPCR (Figure 8a and Supplementary Figure S3A).

### Silencing of RUNX2 decreases HCC cell growth migration and chemoresistance

To ascertain whether *NUPR1* biological effects on regulation of cell growth, migration and chemoresistance were due to regulation of the RUNX2 transcript, we performed *RUNX2* gene silencing in Hep3B cells using specific human siRNA. *RUNX2* gene silencing significantly decreased Hep3B cell growth (Figure 8b) and migration (Figure 8c) and increased cell sensitivity to sorafenib treatment (Figure 8d and Supplementary Figure S3B). Interestingly, in accordance with data reported by Wang *et al.*,^36^ gene expression analysis showed that *RUNX2* suppression strongly downregulates *NUPR1*, as well as *NUPR1*-regulated genes *RELB* and *IER3* (Figure 8e and Supplementary Figure S3C). This result suggests that RUNX2 might regulate *NUPR1* gene expression. Therefore, we performed an *in silico* analysis by using the open-source Jasper database (http://jaspar.genereg.net/) to predict the binding sites of RUNX2 in the promoter region of *NUPR1* gene. This analysis allowed us to identify eight putative RUNX2-binding sites in the *NUPR1* promoter region (Supplementary Table S7 and Supplementary Figure S4). Further studies are needed to clarify whether these binding sites are functional and are involved in the control of *NUPR1* expression by RUNX2. However, these data strongly suggest the existence of a *NUPR1/RELB/IER3/RUNX2* pathway that may act as an auto-regulatory loop.

### Expression of RUNX2 in human HCC samples

Finally, we examined RUNX2 mRNA and protein expression in HCC tissues compared with NL tissues (Figures 8f and g). Real-time PCR analysis showed that RUNX2 mRNA was overexpressed in 8 out of the 12 (66.6%) HCC tissues (Figure 8f), suggesting that *RUNX2*, as well as *NUPR1*, may have an important role in hepatocarcinogenesis. Of note, from the eight samples with high *RUNX2* expression, four cases (50%) also displayed increased *NUPR1* expression. In addition, RUNX2 expression was analyzed by immunohistochemistry. As shown in Figure 8g, high RUNX2 expression was observed in HCC tissues, with clear nuclear localization in the tumor hepatocytes but not in NL hepatocytes. All scores analyzed for RUNX2 were significantly higher in HCC than in NL tissues (Supplementary Table S8A). In addition, the percentage of positive nuclei and the sum of the scores were significantly higher in HCC than in LC tissues (Supplementary Table S8A). Furthermore, the percentage of positive nuclei and the sum scores were significantly different in HCC tissues with different differentiation grades, whereas no correlation was observed between RUNX2 expression and TNM classification (Supplementary Table S8B).

Finally, we found a significant correlation between the percentage of NUPR1- and RUNX2-postive nuclei (*P*<0.05) and the sum of the scores (*P*<0.05) in HCC tissues (Supplementary Table S9).

## Discussion

HCC is a complex disease and currently sorafenib represents the only systemic therapy available for the treatment of advanced disease,^1^ although its benefits are modest. On the other hand, the molecular mechanism of action of sorafenib has not yet been fully clarified. Therefore, a better understanding of the molecular mechanism(s) responsible for its antitumor effect could be critical for improving treatment effectiveness, while minimizing side effects. Several studies have shown that sorafenib blocks tumor cell proliferation and induces cell death through mitogen-extracellular activated protein kinase kinase (MEK)/ERK-dependent and -independent mechanisms.^2, 3, 4, 5, 6, 7^ In particular, ER stress has been demonstrated to be part of the MEK/ERK-independent program of cell death induced by this drug.^8, 9, 10^

In this study, we present potential molecular mechanisms to explain sorafenib resistance in HCC cells. First, confirming and extending previous data,^11, 12^ we found that sorafenib induces, in a time- and dose-dependent manner, the expression of several genes involved in ER stress, such as *GRP78*, *ATF4*, *CHOP* and *TRB3* as well as *NUPR1*, a stress-inducible gene, and the splicing of XBP1 mRNA.

Recently, it has been reported that NUPR1 is overexpressed in several human cancers.^15, 16, 17, 37^ However, studies have demonstrated that NUPR1 can act either as an inducer or suppressor of tumor growth. In the liver, NUPR1 has been reported to be involved in cellular stress response to hepatic injury by CCl_4_.^20^ Recently, Lee *et al.*^21^ identified *NUPR1* as one of the 10 common mitochondrial defect-related genes that may induce a retrograde signaling from cancer cell mitochondria to intracellular transcriptome, having a critical role in liver cancer progression.

In this study, we examined NUPR1 expression status (both protein and mRNA) in HCC patient tissues and in healthy liver tissues. Immunohistochemical analysis revealed that NUPR1 expression level was higher in HCC tissues than in healthy liver tissues, with a nuclear localization only in malignant hepatocytes. In >50% of HCC tissues, we found higher levels of NUPR1 mRNA than in normal and in cirrhotic liver tissues, suggesting its involvement in hepatocarcinogenesis. Of note, we found that NUPR1 mRNA expression was high in both HBV- and HCV-associated HCC, which suggests that not only HBV^22^ but also HCV might regulate *NUPR1* expression during hepatocarcinogenesis.

Furthermore, both our *in vitro* and *in vivo* data confirmed that NUPR1 has a critical role in the regulation of tumor cell growth, tumor migration and invasive capacity and survival of HCC cells, suggesting its oncogenic role in HCC.

Chemoresistance represents a major challenge for HCC treatment. It has been reported that *NUPR1* is involved in chemoresistance in breast and pancreatic cancers.^38, 39^ Here we demonstrated that *NUPR1* depletion led to a significant increase in HCC cell sensitivity to sorafenib treatment, suggesting that it may also have a pivotal role in HCC chemoresistance.

Moreover, and as already reported,^16^ we found that *NUPR1* gene silencing resulted in *RELB* and *IER3* downregulation. The role of IER3 in regulating tumor cell growth is highly controversial. Several studies have demonstrated that *IER3* (also known as IEX-1), similar to *NUPR1*, may act as an oncogenic or pro-apoptotic factor in different human malignancies.^40^ Recently, Kwon *et al.*^41^ demonstrated that IER3 may be considered as a functional biomarker for an aggressive HCC subtype. To better understand whether the biological effects observed in HCC cells after *NUPR1* gene silencing were determined through regulation of these transcripts, we used specific siRNA to silence *RELB* and *IER3* genes and demonstrated that *RELB* and *IER3* knockdown inhibits cell growth and migration and increases cell sensitivity to sorafenib in HCC cells.

To clarify the molecular mechanisms and biological pathways involved in NUPR1-mediated action on HCC cells, we performed a comparative analysis of global gene expression using DNA microarray technology in Hep3B cells upon *NUPR1* knockdown. Microarray data showed that *NUPR1* gene silencing resulted in a strong downregulation of several genes involved in cellular movement, lipid metabolism, molecular transport and cellular response to therapies. IPA pathway and network analyses highlighted some important gene nodes related to *NUPR1* depletion in Hep3B cells that confirmed its functional role observed in HCC cells.

In particular, we found that *NUPR1* knockdown results in upregulation of some important factors known to be involved in the regulation of cell growth and proliferation in HCC. Among these, we observed a strong upregulation of *TGFβ2*, shown to induce cell death in Hep3B cells with low endogenous TGF*β* levels.^42^ In addition, *DKK3*, which acts as a Wnt-antagonist and tumor suppressor, and the *CDKN2B* (coding for p15INK4B protein), which acts as a negative regulator of cell cycle progression,^43, 44^ were found to be upregulated in Hep3B cells upon *NUPR1* suppression. These data confirm the effect of *NUPR1* knockdown in reducing cell growth.

On *NUPR1* suppression, we also observed a strong and significant downregulation of some important genes involved in the regulation of cell proliferation and movement. Among these, *FGF19* has been implicated in HCC development in humans^45, 46^ and in HCC cell proliferation and migration,^46^ as its expression has been suggested to be an independent prognostic factor for overall and disease-free survival.^46^ Of interest, we also found that *NUPR1* gene silencing sharply downregulates *BMP7*. This gene has been reported to be overexpressed in HCC and may be considered as a biomarker for poor prognosis in HCC.^33^ Moreover, in another study Lu *et al.*^47^ demonstrated that overexpression of *BMP7* gene increases HCC cell viability and migration.

Interestingly, our microarray data analysis also provided evidence of the involvement of *NUPR1* in lipid metabolism regulation. We found that several genes, such as *APOM*, *ADH4*, *ACOX2*, *ACSL1* and *SLC1A3*, were downregulated in Hep3B cells upon *NUPR1* suppression.

Finally, we found that *NUPR1* suppression also resulted in *RUNX2* downregulation. Many authors have reported that *RUNX2* has an oncogenic role in different human malignancies,^36, 48^ although its role in HCC still remains unclear. We found that RUNX2 protein was significantly higher in HCC tissues than in LC and NL tissues and that RUNX2 mRNA was overexpressed in 8 out of the 12 (66.6%) HCC patient tissues, with a co-expression of RUNX2 mRNA and NUPR1 mRNA observed in 50% of RUNX2-positive HCC samples. This result was also supported by immunohistochemical analysis, which showed that expression of NUPR1 was significantly correlated with expression of RUNX2 in HCC tissues. Moreover, we demonstrated that *RUNX2* gene silencing significantly decreases cell growth and migration and increases Hep3B cell response to sorafenib treatment. In addition, and as previously reported,^36^ we observed that *RUNX2* suppression results in a strong downregulation of *NUPR1* and of the *NUPR1*-regulated genes *RELB* and *IER3*. These data imply the existence of a *NUPR1/RELB/IER3/RUNX2* pathway that may act as an auto-regulatory loop, which we demonstrated as having a key role in HCC cell growth, migration, invasion and chemoresistance. The identification of a NUPR1/RELB/IER3/RUNX2 pathway as a potential therapeutic target may contribute to the development of new treatment strategies for HCC management.

## Materials and Methods

### Tissue specimens and immunohistochemistry

The study included 17 primary HCC patients with hepatitis virus B (HBV)-associated (*n*=3) and HCV-associated (*n*=14) chronic liver disease (13 male, 4 female; mean age 63 years, range 50–82 years). Fresh tumor and non-tumor samples were collected during surgical resection and used for real-time PCR studies of *NUPR1* and *RUNX2* gene expression. All samples were snap-frozen and stored at −80 °C until RNA extraction. In five cases, paired tumor and surrounding non-tumor regions (cirrhotic tissues) were analyzed. All patients had in fact undergone surgery or liver transplantation at the Division of Surgery of the University Medical School of Palermo, Italy or at the Istituto Mediterraneo Trapianti e Terapie (ISMETT), Palermo, Italy. Informed consent was obtained from all patients. The study protocol is conformed to the ethical guidelines of the 1975 Declaration of Helsinki.

For immunohistochemical analyses, liver cancer tissue microarray (TMA) slides were purchased from Abcam (Abcam, Cambridge, UK). Each TMA slide contains 27 cases in duplicates: 3 NL, 3 premalignant, and 21 cancer tissues with progressive grades (5 Grade I, 12 Grade II and 4 Grade III) and TNM stages (17 T2N0M0, 3 T3N0M0 and 1 T4N0M0). Tissue sections in TMA were deparafinized and rehydrated and the antigen unmasking technique was performed using Novocastra Epitope Retrieval Solutions (Leica Microsystems Srl, Milan, Italy) at pH 6 or pH 9 in PT Link Dako (Dako Italia SRL, Milan, Italy) at 98 °C for 30 min. Subsequently, the sections were brought to room temperature and rinsed in PBS. After neutralization of the endogenous peroxidase with 3% H_2_O_2_ and Fc blocking by a specific protein block, the samples were incubated overnight at 4 °C with the primary antibodies against human NUPR1 (produced by Dr JL Iovanna) and RUNX2 (1:200, pH 6) purchased from Santa Cruz (Santa Cruz Biotechnologies Inc., Dallas, TX, USA). Staining was revealed by Polymer Detection Kit (Novocastra Leica Biosystems, Newcastle, UK) and 3,3′diaminobenzidine (DAB) substrate chromogen. The slides were counterstained with hematoxylin (Novocastra, Leica Biosystems). Negative control staining was performed by using rabbit immune sera instead of the primary antibodies. All the sections were analyzed under a Leica DM 2000 optical microscope (Leica Microsystems, Wetzlar, Germany) and microphotographs were collected using a Leica DFC320 digital camera (Leica).

The relative percentages of positive nuclei for NUPR1 or RUNX2, as well as staining intensity, were evaluated by two independent observers (AG and VC). Staining intensity was classified as follows: 0 (no staining), 1 (weak), 2 (moderate), and 3 (strong). Percentages of positive nuclei were classified as follows: 0 (no positive tumor nuclei), 1 (1–25% positive), 2 (26–50% positive), 3 (51–75% positive), and 4 (76–100% positive). The scores for staining intensity percentages and for positive nuclei were added together to give a single immunohistochemical staining score from 0 to 7.

### Reagents and cell culture

Sorafenib was purchased from Alexis Biochemical (Lausen, CH, Switzerland) and dissolved in dimethyl sulfoxide (DMSO). The human HCC cell lines HepG2, Hep3B, PLC/PRF/5 and Huh7 used in this study had a low passage number and were maintained as previously reported.^49^ HepG2 and Hep3B cells were obtained from the American Type Culture Collection (ATCC, Rockville, MD, USA). PLC/PRF/5 cells used in this study were a gift from Professor O Bussolati (University of Parma, Parma, Italy) and Huh7 were a gift from Professor M Levrero (Sapienza University of Rome, Rome, Italy). All cell lines were authenticated by short tandem repeat profiling (BMR Genomics, Padua, Italy), and used within 6 months of receipt. All cell cultures were routinely tested and found to be free of mycoplasma contamination.

### Immunofluorescence

HCC cells were seeded in chamber slides at 1.0 × 10^4^ cells/well in RPMI 10% FBS. After 24 h, cells were treated with 7.5 and 10 *μ*M sorafenib for 3 h. Cells treated with DMSO were used as controls. After treatment, cells were fixed, permeabilized and stained with primary anti-NUPR1 antibody. After staining, slides were mounted using Vectashield mounting medium with DAPI (Vector Laboratories Inc., Peterborough, UK) to visualize cell nuclei. Images were acquired with a Leica microscope.

### SiRNA transfection

Cells were seeded into 60-mm plates using medium without antibiotics and 24 h later transfected with 75 nmol/l siRNA targeting human-specific genes. Transfections were performed using the Lipofectamine RNAi Max reagent (Invitrogen, Carlsbad, CA, USA), according to the manufacturer's instructions. The following specific siRNAs were purchased from QIAGEN (Germantown, MD, USA): siNUPR1 #1 (SI02664326); siRELB #2 (SI00089117); siIER3 #2 (SI00057540); siRUNX2 #2 (SI00062993) and from Santa Cruz: siNUPR1 #2 (sc-40792); siRELB #1 (sc-36402); siIER3 #1 (sc-43859); and siRUNX2 #1 (sc-37145). Negative control siRNA (siNC) was purchased from QIAGEN (1027281).

### Extraction of cellular RNA and quantitative real-time PCR (qPCR)

Total RNA was extracted using TRIzol reagent according to the manufacturer's instructions. In all, 1.5 *μ*g of total RNA were subjected to reverse transcription to generate cDNA and qPCR was performed as previously reported.^49^ mRNA level was evaluated using specific QuantiTect Primer Assays (QIAGEN) as previously reported.^11, 49^ All samples were analyzed in triplicate.

### Cell viability and clonogenic assays

Twenty-four hours after transfection, cells were detached and plated in 96-well plates for MTS assay or 60-mm plates for colony assay and mRNA expression analysis. Transfected cells were exposed to sorafenib for an additional period of 48 h and then analyzed for cell viability. MTS assays were performed as previously reported.^49^ Each experiment was performed in triplicate and repeated three times. Cell viability was expressed as a percentage of the absorbance measured in the control cells. Values were expressed as means±S.D.

The effect of *NUPR1*, *RELB*, *IER3* and *RUNX2* gene silencing on cell viability was also assessed using a clonogenic assay, as previously described.^11, 12^ Relative colony formation was determined by the ratio of the average number of colonies in cells transfected with human-specific siRNA for *NUPR1*, *RELB*, *IER3* and *RUNX2* to the average number of colonies in siNC-transfected cells. All experiments were performed in duplicate and repeated three times.

### Wound healing, cell migration and invasion assays

For wound-healing assay 24 h after transfection, 5.0 × 10^5^ cells transfected with human *NUPR1* siRNA and with siNC were plated on 35-mm plates until confluent. Cells were scratched using 200-*μ*l tips, washed and cultured in fresh media. Transwell cell migration and invasion assays were performed as previously described.^36^

### Stable shRNA-mediated NUPR1 transfection

For stable clone generation, Hep3B cells were seeded in six-well plates in RPMI medium without antibiotics. After 24 h, cells were transfected with 2 *μ*g of *NUPR1*-specific shRNA or scramble shRNA (pSilencer), containing a region for resistance to puromycin, and Lipofectamine 2000 (Invitrogen), according to the manufacturer's instructions. Stable clones were selected in 2 *μ*g/ml puromycin for 8 weeks, after which single clones were selected using a limited dilution technique. *NUPR1* gene silencing was tested by qPCR.

### NUPR1 shRNA stable clone cell viability

Cell proliferation (cell index) was analyzed using the xCELLigence Real-Time Cell Analyzer instrument, as previously reported.^36^ In addition, to evaluate the effect of *NUPR1* shRNA-mediated knockdown on cell response to sorafenib treatment, cells were plated in six-well plates at 3.0 × 10^5^/well and treated with different concentrations of sorafenib. Cells were trypsinized 48 h after treatment and counted after staining with 0.4% (w/v) trypan blue (Sigma-Aldrich Srl, Milan, Italy).

### Western blotting analyses

Whole-cell lysates were obtained using RIPA buffer (Cell Signaling Technologies Inc., Danvers, MA, USA) and western blotting analyses were performed as previously described,^49^ with primary antibodies raised against ERK1/2, phospho-ERK1/2 (Cell Signaling) and *β*-actin (Sigma-Aldrich).

### *In vivo* studies

A total of 10 × 10^6^ Hep3B cells stably expressing shRNA against *NUPR1* or control shRNA (pSilencer) were implanted subcutaneously in female nude athymic mice (Fox1 nu/nu) (6 weeks old, male; *n*=12) obtained from Envigo (Udine, Italy). The mice were examined for tumor formation for 4 weeks and were killed in conformity with institutional guidelines, which are in compliance with national (D.L., 116 G.U., Suppl.40; 18 February 1992) and international laws and policies (ECC Council Directive 86/609, OJ L358.1, 12 December 1987). This study was authorized by the Italian Ministry of Health.

### Bioinformatics, gene expression profiling and data analysis

Nine HCC public data sets were analyzed for the pattern of *NUPR1* gene mRNA expression by the Oncomine software (https://www.oncomine.com). Clinical and pathological features were available in four data sets (Supplementary Table S1). Expression fold change, data normalization and the statistical significance of the differential analysis data were obtained using Oncomine algorithms.

Open-source Jasper database (http://jaspar.genereg.net/) was used to predict the binding sites of RUNX2 in the promoter region of the *NUPR1* gene. This bioinformatics tool includes a collection of transcription factor DNA-binding preferences, modeled as matrices. To this purpose, the sequence Chr16:28535905-28541804 (GeneBank: Chromosome 16, NC_000016.10 – GRCh38.p2 Primary Assembly), including 3003 bp downstream and 2897 bp upstream ATG codon, here considered as *NUPR1* promoter region, was selected for the Jaspar prediction analysis. In this analysis, only the predicted sites of RUNX2 factor with a relative profile score threshold >80% was considered.

Gene expression was analyzed using Agilent 44K Human Whole Genome Oligonucleotide Microarrays (containing ~44 000 genes) (Agilent, Palo Alto, CA, USA), as previously described.^11, 12, 36^ All microarray experiments were performed in duplicate, using dye-swap during labeling. The GeneSpring software (Agilent) was used to generate lists of selected genes for the different statistical and visualization methods. Network and pathway analyses of the microarray data were completed using the IPA software (http://www.Ingenuity.com). The microarray data were deposited in the GEO database with accession number GSE73521.

### Statistical analysis

Statistical analysis was performed using Student's *t*-test (two-tailed), with *P*<0.05. The Mann–Whitney *U*-test and Spearman's rank correlation test were used when appropriate.

## Abbreviations

ATF4: activating transcription factor 4
BMP7: bone morphogenetic protein 7
CDKN2B: cyclin-dependent kinase inhibitor 2B
CHOP: C/EBP-homologous protein
DKK3: Dickkopf 3
ER: endoplasmic reticulum
ERK: extracellular signal-regulated kinase
FGF19: fibroblast growth factor 19
HCC: hepatocellular carcinoma
IER3: immediate early response 3
MEK: mitogen-extracellular activated protein kinase kinase
NUPR1: nuclear protein-1
qPCR: quantitative real-time PCR
RELB: V-rel avian reticuloendotheliosis viral oncogene homolog B
RUNX2: Runt-related transcription factor 2
shRNA: short hairpin RNA
siRNA: small interference RNA
TGF*β*2: transforming growth factor *β*2
TRB3: Tribbles homolog 3
XBP1: X-box-binding protein 1.

## References

[bib1] Cervello M, McCubrey JA, Cusimano A, Lampiasi N, Azzolina A, Montalto G. Targeted therapy for hepatocellular carcinoma: novel agents on the horizon. Oncotarget 2012; 3: 236–260. 22470194 10.18632/oncotarget.466 PMC3359882

[bib2] Wilhelm SM, Carter C, Tang L, Wilkie D, McNabola A, Rong H et al. Bay 43-9006 exhibits broad spectrum oral antitumor activity and targets the Raf/MEK/ERK pathway and receptor tyrosine kinases involved in tumor progression and angiogenesis. Cancer Res 2004; 64: 7099–7109. 15466206 10.1158/0008-5472.CAN-04-1443

[bib3] Liu L, Cao Y, Chen C, Zhang X, McNabola A, Wilkie D et al. Sorafenib blocks the RAF/MEK/ERK pathway, inhibits tumor angiogenesis, and induces tumor cell apoptosis in hepatocellular carcinoma model PLC/PRF/5. Cancer Res 2006; 66: 11851–11858. 17178882 10.1158/0008-5472.CAN-06-1377

[bib4] Tai WT, Cheng AL, Shiau CW, Huang HP, Huang JW, Chen PJ et al. Signal transducer and activator of transcription 3 is a major kinase-independent target of sorafenib in hepatocellular carcinoma. J Hepatol 2011; 55: 1041–1048. 21354226 10.1016/j.jhep.2011.01.047

[bib5] Yi P, Higa A, Taouji S, Bexiga MG, Marza E, Arma D et al. Sorafenib-mediated targeting of the AAA+ ATPase p97/VCP leads to disruption of the secretory pathway, endoplasmic reticulum stress, and hepatocellular cancer cell death. Mol Cancer Ther 2012; 11: 2610–2620. 23041544 10.1158/1535-7163.MCT-12-0516

[bib6] Fiume L, Manerba M, Vettraino M, Di Stefano G. Effect of sorafenib on the energy metabolism of hepatocellular carcinoma cells. Eur J Pharmacol 2011; 670: 39–43. 21924262 10.1016/j.ejphar.2011.08.038

[bib7] Tesori V, Piscaglia AC, Samengo D, Barba M, Bernardini C, Scatena R et al. The multikinase inhibitor sorafenib enhances glycolysis and synergizes with glycolysis blockade for cancer cell killing. Sci Rep 2015; 5: 9149. 25779766 10.1038/srep09149 PMC4361992

[bib8] Inamoto T, Azuma H. Sorafenib increases endoplasmic reticulum (ER) stress in concert with vorinostat. Cancer Biol Ther 2011; 12: 1018. 22095133 10.4161/cbt.12.12.18135

[bib9] Shi YH, Ding ZB, Zhou J, Hui B, Shi GM, Ke AW et al. Targeting autophagy enhances sorafenib lethality for hepatocellular carcinoma via ER stress-related apoptosis. Autophagy 2011; 7: 1159–1172. 21691147 10.4161/auto.7.10.16818

[bib10] Holz MS, Janning A, Renné C, Gattenlöhner S, Spieker T, Bräuninger A. Induction of endoplasmic reticulum stress by sorafenib and activation of NF-κB by lestaurtinib as a novel resistance mechanism in Hodgkin lymphoma cell lines. Mol Cancer Ther 2013; 12: 173–183. 23243060 10.1158/1535-7163.MCT-12-0532

[bib11] Cervello M, Bachvarov D, Lampiasi N, Cusimano A, Azzolina A, McCubrey JA et al. Molecular mechanisms of sorafenib action in liver cancer cells. Cell Cycle 2012; 11: 2843–2855. 22801548 10.4161/cc.21193

[bib12] Cervello M, Bachvarov D, Lampiasi N, Cusimano A, Azzolina A, McCubrey JA et al. Novel combination of sorafenib and celecoxib provides synergistic anti-proliferative and pro-apoptotic effects in human liver cancer cells. PLoS One 2013; 8: e65569. 23776502 10.1371/journal.pone.0065569 PMC3680460

[bib13] Mallo GV, Fiedler F, Calvo EL, Ortiz EM, Vasseur S, Keim V et al. Cloning and expression of the rat p8 cDNA, a new gene activated in pancreas during the acute phase of pancreatitis, pancreatic development, and regeneration, and which promotes cellular growth. J Biol Chem 1997; 272: 32360–32369. 9405444 10.1074/jbc.272.51.32360

[bib14] Cano CE, Hamidi T, Sandi MJ, Iovanna JL. Nupr1: the Swiss-knife of cancer. J Cell Physiol 2011; 226: 1439–1443. 20658514 10.1002/jcp.22324

[bib15] Carracedo A, Lorente M, Egia A, Blázquez C, García S, Malicet C et al. The stress regulated protein p8 mediates cannabinoid-induced apoptosis of tumor cells. Cancer Cell 2006; 9: 301–312. 16616335 10.1016/j.ccr.2006.03.005

[bib16] Hamidi T, Algül H, Cano CE, Sandi MJ, Molejon MI, Riemann M et al. Nuclear protein 1 promotes pancreatic development and protects cell from stress by inhibiting apoptosis. J Clin Invest 2012; 122: 2092–2103. 22565310 10.1172/JCI60144 PMC3366404

[bib17] Sandi MJ, Hamidi T, Malicet C, Cano C, Loncle C, Pierres A et al. p8 controls pancreatic cancer cells migration, invasion, adhesion and tumorigenesis. J Cell Physiol 2011; 226: 3442–3451. 21344397 10.1002/jcp.22702

[bib18] Giroux V, Malicet C, Barthet M, Gironella M, Archange C, Dagorn JC et al. p8 is a new target of gemcitabine in pancreatic cancer cells. Clin Cancer Res 2006; 12: 235–241. 16397047 10.1158/1078-0432.CCR-05-1700

[bib19] Vincent AJ, Ren S, Harris LG, Devine DJ, Samant RS, Fodstad O et al. Cytoplasmic translocation of p21 mediates NUPR1-induced chemoresistance: NUPR1 and p21 in chemoresistance. FEBS Lett 2012; 586: 3429–3434. 22858377 10.1016/j.febslet.2012.07.063

[bib20] Taïeb D, Malicet C, Garcia S, Rocchi P, Arnaud C, Dagorn JC et al. Inactivation of stress protein p8 increases murine carbon tetrachloride hepatotoxicity via preserved CYP2E1 activity. Hepatology 2005; 42: 176–182. 15962327 10.1002/hep.20759

[bib21] Lee YK, Jee BA, Kwon SM, Yoon YS, Xu WG, Wang HJ et al. Identification of a mitochondrial defect gene signature reveals NUPR1 as a key regulator of liver cancer progression. Hepatology 2015; 62: 1174–1189. 26173068 10.1002/hep.27976 PMC6312643

[bib22] Bak Y, Shin HJ, Bak IS, Yoon DY, Yu DY. Hepatitis B virus X promotes hepatocellular carcinoma development via nuclear protein 1 pathway. Biochem Biophys Res Commun 2015; 466: 676–681. 26392315 10.1016/j.bbrc.2015.09.082

[bib23] Archer KJ, Mas VR, David K, Maluf DG, Bornstein K, Fisher RA. Identifying genes for establishing a multigenic test for hepatocellular carcinoma surveillance in hepatitis C virus-positive cirrhotic patients. Cancer Epidemiol Biomarkers Prev 2009; 18: 2929–2932. 19861515 10.1158/1055-9965.EPI-09-0767 PMC2783293

[bib24] Chen X, Cheung ST, So S, Fan ST, Barry C, Higgins J et al. Gene expression patterns in human liver cancers. Mol Biol Cell 2002; 13: 1929–1939. 12058060 10.1091/mbc.02-02-0023. PMC117615

[bib25] Chiang DY, Villanueva A, Hoshida Y, Peix J, Newell P, Minguez B et al. Focal gains of VEGFA and molecular classification of hepatocellular carcinoma. Cancer Res 2008; 68: 6779–6788. 18701503 10.1158/0008-5472.CAN-08-0742 PMC2587454

[bib26] Jia HL, Ye QH, Qin LX, Budhu A, Forgues M, Chen Y et al. Gene expression profiling reveals potential biomarkers of human hepatocellular carcinoma. Clin Cancer Res 2007; 13: 1133–1139. 17317821 10.1158/1078-0432.CCR-06-1025

[bib27] Liao YL, Sun YM, Chau GY, Chau YP, Lai TC, Wang JL et al. Identification of SOX4 target genes using phylogenetic footprinting-based prediction from expression microarrays suggests that overexpression of SOX4 potentiates metastasis in hepatocellular carcinoma. Oncogene 2008; 27: 5578–5589. 18504433 10.1038/onc.2008.168

[bib28] Mas VR, Maluf DG, Archer KJ, Yanek K, Kong X, Kulik L et al. Genes involved in viral carcinogenesis and tumor initiation in hepatitis C virus-induced hepatocellular carcinoma. Mol Med 2009; 15: 85–94. 19098997 10.2119/molmed.2008.00110 PMC2605622

[bib29] Roessler S, Jia HL, Budhu A, Forgues M, Ye QH, Lee JS et al. A unique metastasis gene signature enables prediction of tumor relapse in early-stage hepatocellular carcinoma patients. Cancer Res 2010; 70: 10202–10212. 21159642 10.1158/0008-5472.CAN-10-2607 PMC3064515

[bib30] Wurmbach E, Chen YB, Khitrov G, Zhang W, Roayaie S, Schwartz M et al. Genome-wide molecular profiles of HCV-induced dysplasia and hepatocellular carcinoma. Hepatology 2007; 45: 938–947. 17393520 10.1002/hep.21622

[bib31] Letourneux C, Rocher G, Porteu F. B56-containing PP2A dephosphorylate ERK and their activity is controlled by the early gene IEX-1 and ERK. EMBO J 2006; 25: 727–738. 16456541 10.1038/sj.emboj.7600980 PMC1383561

[bib32] Garcia MN, Grasso D, Lopez-Millan MB, Hamidi T, Loncle C, Tomasini R et al. IER3 supports KRASG12D-dependent pancreatic cancer development by sustaining ERK1/2 phosphorylation. J Clin Invest 2014; 124: 4709–4722. 25250570 10.1172/JCI76037 PMC4347237

[bib33] Li W, Cai HX, Ge XM, Li K, Xu WD, Shi WH. Prognostic significance of BMP7 as an oncogene in hepatocellular carcinoma. Tumor Biol 2013; 34: 669–674. 10.1007/s13277-012-0594-x 23179403

[bib34] Tou L, Quibria N, Alexander JM. Transcriptional regulation of the human Runx2/Cbfa1 gene promoter by bone morphogenetic protein-7. Mol Cell Endocrinol 2003; 205: 121–129. 12890574 10.1016/s0303-7207(03)00151-5

[bib35] Gu K, Zhang L, Jin T, Rutherford RB. Identification of potential modifiers of Runx2/Cbfa1 activity in C2C12 cells in response to bone morphogenetic protein-7.Cells. Tissues Organs 2004; 176: 28–40. 10.1159/000075025 14745233

[bib36] Wang ZQ, Keita M, Bachvarova M, Gobeil S, Morin C, Plante M et al. Inhibition of RUNX2 transcriptional activity blocks the proliferation, migration and invasion of epithelial ovarian carcinoma cells. PLoS One 2013; 8: e74384. 24124450 10.1371/journal.pone.0074384 PMC3790792

[bib37] Ree AH, Pacheco MM, Tvermyr M, Fodstad O, Brentani MM. Expression of a novel factor, com1, in early tumor progression of breast cancer. Clin Cancer Res 2000; 6: 1778–1783. 10815897

[bib38] Cano CE, Iovanna JL. Stress proteins and pancreatic cancer metastasis. Scientific World Journal 2010; 10: 1958–1966. 20890585 10.1100/tsw.2010.186 PMC5763959

[bib39] Ree AH, Tvermyr M, Engebraaten O, Rooman M, Røsok O, Hovig E et al. Expression of a novel factor in human breast cancer cells with metastatic potential. Cancer Res 1999; 59: 4675–4680. 10493524

[bib40] Arlt A, Schäfer H. Role of the immediate early response 3 (IER3) gene in cellular stress response, inflammation and tumorigenesis. Eur J Cell Biol 2011; 90: 545–552. 21112119 10.1016/j.ejcb.2010.10.002

[bib41] Kwon SM, Kim DS, Won NH, Park SJ, Chwae YJ, Kang HC et al. Genomic copy number alterations with transcriptional deregulation at 6p identify an aggressive HCC phenotype. Carcinogenesis 2013; 34: 1543–1550. 23508637 10.1093/carcin/bgt095

[bib42] Dzieran J, Fabian J, Feng T, Coulouarn C, Ilkavets I, Kyselova A et al. Comparative analysis of TGF-β/Smad signaling dependent cytostasis in human hepatocellular carcinoma cell lines. PLoS One 2013; 8: e72252. 23991075 10.1371/journal.pone.0072252 PMC3750029

[bib43] Ding Z, Qian YB, Zhu LX, Xiong QR. Promoter methylation and mRNA expression of DKK-3 and WIF-1 in hepatocellular carcinoma. World J Gastroenterol 2009; 15: 2595–2601. 19496188 10.3748/wjg.15.2595 PMC2691489

[bib44] Ren WH, Li YW, Li R, Feng HB, Wu JL, Wang HR. P15 gene methylation in hepatocellular carcinomas: a systematic review and meta-analysis. Int J Clin Exp Med 2015; 8: 4762–4768. 26131050 PMC4483910

[bib45] Miura S, Mitsuhashi N, Shimizu H, Kimura F, Yoshidome H, Otsuka M et al. Fibroblast growth factor 19 expression correlates with tumor progression and poorer prognosis of hepatocellular carcinoma. BMC Cancer 2012; 12: 56. 22309595 10.1186/1471-2407-12-56 PMC3293719

[bib46] Sawey ET, Chanrion M, Cai C, Wu G, Zhang J, Zender L et al. Identification of a therapeutic strategy targeting amplified FGF19 in liver cancer by oncogenomic screening. Cancer Cell 2011; 19: 347–358. 21397858 10.1016/j.ccr.2011.01.040 PMC3061399

[bib47] Lu JW, Hsia Y, Yang WY, Lin YI, Li CC, Tsai TF et al. Identification of the common regulators for hepatocellular carcinoma induced by hepatitis B virus X antigen in a mouse model. Carcinogenesis 2012; 33: 209–219. 22021908 10.1093/carcin/bgr224

[bib48] Owens TW, Rogers RL, Best SA, Ledger A, Mooney AM, Ferguson A et al. Runx2 is a novel regulator of mammary epithelial cell fate in development and breast cancer. Cancer Res 2014; 74: 5277–5286. 25056120 10.1158/0008-5472.CAN-14-0053 PMC4178131

[bib49] Cusimano A, Azzolina A, Iovanna JL, Bachvarov D, McCubrey JA, D'Alessandro N et al. Novel combination of celecoxib and proteasome inhibitor MG132 provides synergistic antiproliferative and proapoptotic effects in human liver tumor cells. Cell Cycle 2010; 9: 1399–1410. 20305374 10.4161/cc.9.7.11254

