# Supplementary material for: NUPR1, a new target in liver cancer: implication in controlling cell growth, migration, invasion and sorafenib resistance
Source: Cell Death Dis. 2016 Jun 23;7(6):e2269–. doi: 10.1038/cddis.2016.175 (PMC5143401; doi:10.1038/cddis.2016.175)
Supplement: Supplementary Table S1 [file cddis2016175x1.doc]

**Supplementary Table 9.** Spearman’s rank correlation between NUPR1 and RUNX2 expression in HCC tissues.

|  | HCC  (n=21) | |
| --- | --- | --- |
|  | ρ | *p* |
| intensity NUPR1 vs intensity RUNX2 | 0.12 | ns |
| % of positive nuclei NUPR1 vs % of positive nuclei RUNX2 | 0.47 | 0.05 |
| sum NUPR1 vs sum RUNX2 | 0.41 | 0.05 |
