# Supplementary material for: NUPR1, a new target in liver cancer: implication in controlling cell growth, migration, invasion and sorafenib resistance
Source: Cell Death Dis. 2016 Jun 23;7(6):e2269–. doi: 10.1038/cddis.2016.175 (PMC5143401; doi:10.1038/cddis.2016.175)
Supplement: Supplementary Table S2 [file cddis2016175x2.doc]

| **Supplementary Table 2.** Differential analysis of NUPR1 gene expression in liver cancer (www.oncomine.org) | | | | | | |
| --- | --- | --- | --- | --- | --- | --- |
|  | **Analysis Type** | | **p value (T test)** | **Fold change** | **Dataset Name** |  |
|  | **Cancer Precursor versus Normal** | | | | |  |
|  |  | Cirrhosis (13) vs. Normal (10) | 2.84E-05 | -1.727 | Wurmbach E *et al*. (30) |  |
|  |  | Liver Cell Dysplasia (17) vs. Normal (10) | 1.61E-04 | -1.531 | Wurmbach E *et al*. (30) |  |
|  |  | Cirrhosis (58) vs. Normal (19) | 6.00E-03 | -1.278 | Mas VR E *et al*. (28) |  |
|  | **Cancer versus Normal** | | | | |  |
|  |  | HCC (104) vs. Normal (76) | 4.18E-08 | 2.323 | Chen X *et al*. (24) |  |
|  |  | HCC (225) vs. Normal (220) | 1.60E-16 | 1.824 | Roessler S *et al*. (29) |  |
|  | **Cancer versus Cancer Precursor** | | | | |  |
|  |  | HCC (104) versus Cancer Precursor (7) | 1.90E-02 | 2.323 | Chen X *et al* (24) |  |
|  |  | HCC (38) versus Cancer Precursor (58) | 6.66E-04 | 1.383 | Mas VR E *et al* (28) |  |
|  |  | HCC (35) versus Cancer Precursor (30) | 1.00E-03 | 1.620 | Wurmbach E *et al* (30) |  |
|  |  | HCC (16) versus Cancer Precursor (47) | 6.00E-03 | -1.303 | Archer KJ *et al* (23) |  |
|  | **Cancer subtype analysis** | | | | |  |
|  |  | TP53 Mutation (11) versus TP53 WT (74) | 0.026 | 1.638 | Chiang DY *et al*. (25) |  |
|  |  | Advanced Stage (39) vs. Early Stage (199) | 0.010 | -1.486 | Jia HL *et al* (26) |  |
|  |  |  |  |  |  |  |
