# Supplementary material for: NUPR1, a new target in liver cancer: implication in controlling cell growth, migration, invasion and sorafenib resistance
Source: Cell Death Dis. 2016 Jun 23;7(6):e2269–. doi: 10.1038/cddis.2016.175 (PMC5143401; doi:10.1038/cddis.2016.175)
Supplement: Supplementary Table S3 [file cddis2016175x3.doc]

**Supplementary Table 3A.** Scores representing immunoreactivity for NUPR1

|  | NL  (n=3) | LC  (n=3) | HCC  (n=21) |
| --- | --- | --- | --- |
| intensity of staining | 1  (1-2) | 2.66  (2-3) | 3  (1-3) |
| % of positive nuclei | 3  (2-3) | 3  (3-4) | 4  (2-4) |
| sum of the scores | 4  (3-5) | 5  (5-7) | 7^a^  (3-7) |

Data are expressed as median (min-max) of the scores.

a, *p* < 0.05 compared with scores of NL

**Supplementary Table 3B.** Correlation of NURP1 expression with TNM and Grade

|  | TNM  (n=21) | *p* | Grade  (n=21) | *p* |
| --- | --- | --- | --- | --- |
| intensity | -0.004 | ns | 0.35 | ns |
| % positive nuclei | -0.024 | ns | 0.47 | 0.05 |
| sum | 0.021 | ns | 0.38 | ns |
