# Supplementary material for: NUPR1, a new target in liver cancer: implication in controlling cell growth, migration, invasion and sorafenib resistance
Source: Cell Death Dis. 2016 Jun 23;7(6):e2269–. doi: 10.1038/cddis.2016.175 (PMC5143401; doi:10.1038/cddis.2016.175)
Supplement: Supplementary Table S4 [file cddis2016175x4.doc]

| **Supplementary Table 4.** Genes, differentially expressed in HCC cells (≥ 3.0 fold, P≤0.05) following NUPR/p81 knockdown | | | |
| --- | --- | --- | --- |
| **GeneName** | **Accessions** | **Description** | **Fold-Change** |
| **A. Upregulated genes** | | | |
| IGFBP7 | ref|NM_001553|ens|ENST00000295666|gb|CR612946|gb|CR607474 | ref|Homo sapiens insulin-like growth factor binding protein 7 (IGFBP7), mRNA [NM_001553 | 25,6228 |
| MAGEA1 | ref|NM_004988|ens|ENST00000356661|gb|BC017555|thc|THC2476756 | ref|Homo sapiens melanoma antigen family A, 1 (directs expression of antigen MZ2-E) (MA | 16,2996 |
| LIMCH1 | ref|NM_014988|ref|NM_001112717|ref|NM_001112718|ref|NM_001112719 | ref|Homo sapiens LIM and calponin homology domains 1 (LIMCH1), transcript variant 1, mR | 15,9881 |
| LYPD1 | ref|NM_144586|ref|NM_001077427|gb|AK123029|gb|BC040046 | ref|Homo sapiens LY6/PLAUR domain containing 1 (LYPD1), transcript variant 1, mRNA [NM_ | 15,7919 |
| SULT1E1 | ref|NM_005420|ens|ENST00000226444|gb|CR407621|gb|AK312236 | ref|Homo sapiens sulfotransferase family 1E, estrogen-preferring, member 1 (SULT1E1), m | 15,781 |
| SLC16A12 | ref|NM_213606|gb|AK124901|ens|ENST00000341233|gb|BC086873 | ref|Homo sapiens solute carrier family 16, member 12 (monocarboxylic acid transporter 1 | 14,6811 |
| CLIC6 | ref|NM_053277|gb|AF448439|ens|ENST00000360731|ens|ENST00000349499 | ref|Homo sapiens chloride intracellular channel 6 (CLIC6), mRNA [NM_053277] | 14,6115 |
| GJA1 | ref|NM_000165|ens|ENST00000282561|gb|CR593006|gb|X52947 | ref|Homo sapiens gap junction protein, alpha 1, 43kDa (GJA1), mRNA [NM_000165] | 13,7642 |
| THC2545097 | thc|THC2545097 | thc|IBP7_HUMAN (Q16270) Insulin-like growth factor-binding protein 7 precursor (IGFBP-7 | 13,5182 |
| SLC16A2 | ref|NM_006517|gb|AB085789|ens|ENST00000276033|gb|U05315 | ref|Homo sapiens solute carrier family 16, member 2 (monocarboxylic acid transporter 8) | 13,4491 |
| NPY1R | ref|NM_000909|ens|ENST00000296533|gb|BC071720|gb|A26481 | ref|Homo sapiens neuropeptide Y receptor Y1 (NPY1R), mRNA [NM_000909] | 13,3851 |
| AK022971 | gb|AK022971|thc|THC2661715 | gb|Homo sapiens cDNA FLJ12909 fis, clone NT2RP2004400. [AK022971] | 12,5488 |
| PAK7 | ref|NM_020341|ref|NM_177990|ens|ENST00000353224|ens|ENST00000378429 | ref|Homo sapiens p21 protein (Cdc42/Rac)-activated kinase 7 (PAK7), transcript variant | 12,4521 |
| EFEMP1 | ref|NM_004105|ref|NM_001039348|ref|NM_001039349|gb|BC098561 | ref|Homo sapiens EGF-containing fibulin-like extracellular matrix protein 1 (EFEMP1), t | 11,6409 |
| GPX8 | ref|NM_001008397|ens|ENST00000296734|gb|AK298545|gb|CR620005 | ref|Homo sapiens glutathione peroxidase 8 (putative) (GPX8), mRNA [NM_001008397] | 11,4669 |
| OXTR | ref|NM_000916|ens|ENST00000316793|gb|X64878|thc|THC2602535 | ref|Homo sapiens oxytocin receptor (OXTR), mRNA [NM_000916] | 11,2277 |
| MMP7 | ref|NM_002423|ens|ENST00000260227|gb|DQ893132|gb|EU176770 | ref|Homo sapiens matrix metallopeptidase 7 (matrilysin, uterine) (MMP7), mRNA [NM_00242 | 10,5474 |
| UGT2B15 | ref|NM_001076|ref|NM_001077|ens|ENST00000338206|ens|ENST00000309229 | ref|Homo sapiens UDP glucuronosyltransferase 2 family, polypeptide B15 (UGT2B15), mRNA | 9,55165 |
| LYPD1 | ref|NM_144586|ref|NM_001077427|gb|BC017318|gb|AK130005 | ref|Homo sapiens LY6/PLAUR domain containing 1 (LYPD1), transcript variant 1, mRNA [NM_ | 9,04865 |
| SLC1A1 | ref|NM_004170|ens|ENST00000262352|ens|ENST00000381910|gb|AB008536 | ref|Homo sapiens solute carrier family 1 (neuronal/epithelial high affinity glutamate t | 8,77086 |
| C6 | ref|NM_000065|ref|NM_001115131|ens|ENST00000263413|ens|ENST00000337836 | ref|Homo sapiens complement component 6 (C6), transcript variant 1, mRNA [NM_000065] | 8,57045 |
| SLC4A4 | ref|NM_003759|ref|NM_001098484|ref|NM_001134742|ens|ENST00000264485 | ref|Homo sapiens solute carrier family 4, sodium bicarbonate cotransporter, member 4 (S | 8,49677 |
| CXCR4 | ref|NM_001008540|ref|NM_003467|ens|ENST00000241393|ens|ENST00000409817 | ref|Homo sapiens chemokine (C-X-C motif) receptor 4 (CXCR4), transcript variant 1, mRNA | 8,47716 |
| BAAT | ref|NM_001701|ref|NM_001127610|ens|ENST00000259407|ens|ENST00000395051 | ref|Homo sapiens bile acid Coenzyme A: amino acid N-acyltransferase (glycine N-choloylt | 8,30714 |
| SUCNR1 | ref|NM_033050|gb|BC030948|gb|AK290420|gb|AF348078 | ref|Homo sapiens succinate receptor 1 (SUCNR1), mRNA [NM_033050] | 8,16803 |
| TMEM156 | ref|NM_024943|ens|ENST00000381938|ens|ENST00000344606|gb|AK026888 | ref|Homo sapiens transmembrane protein 156 (TMEM156), mRNA [NM_024943] | 8,15836 |
| THC2637568 | thc|THC2637568 | Unknown | 8,01147 |
| TM4SF1 | ref|NM_014220|ens|ENST00000383054|ens|ENST00000305366|gb|CR456953 | ref|Homo sapiens transmembrane 4 L six family member 1 (TM4SF1), mRNA [NM_014220] | 7,97581 |
| MAGEA2B | ref|NM_153488|ref|NM_175743|ref|NM_005361|ref|NM_175742 | ref|Homo sapiens melanoma antigen family A, 2B (MAGEA2B), mRNA [NM_153488] | 7,93932 |
| CTTNBP2 | ref|NM_033427|gb|BC106000|ens|ENST00000160373|gb|AB051545 | ref|Homo sapiens cortactin binding protein 2 (CTTNBP2), mRNA [NM_033427] | 7,81571 |
| PSG11 | ref|NM_002785|ref|NM_203287|ref|NM_001113410|ens|ENST00000401740 | ref|Homo sapiens pregnancy specific beta-1-glycoprotein 11 (PSG11), transcript variant | 7,78848 |
| TCEAL3 | ref|NM_001006933|ref|NM_032926|ref|NM_001006938|gb|BC071675 | ref|Homo sapiens transcription elongation factor A (SII)-like 3 (TCEAL3), transcript va | 7,74703 |
| TCEAL6 | ref|NM_001006938|ref|NM_001006933|ref|NM_032926|gb|BC071675 | ref|Homo sapiens transcription elongation factor A (SII)-like 6 (TCEAL6), mRNA [NM_0010 | 7,62043 |
| NPFFR2 | ref|NM_053036|ref|NM_004885|ens|ENST00000308744|ens|ENST00000358749 | ref|Homo sapiens neuropeptide FF receptor 2 (NPFFR2), transcript variant 2, mRNA [NM_05 | 7,60307 |
| AL049227 | gb|AL049227|thc|THC2505815 | gb|Homo sapiens mRNA; cDNA DKFZp564N1116 (from clone DKFZp564N1116). [AL049227] | 7,57824 |
| TMEM185A | ref|NM_032508|ens|ENST00000316916|gb|AK128688|gb|AK096308 | ref|Homo sapiens transmembrane protein 185A (TMEM185A), mRNA [NM_032508] | 7,44089 |
| THSD7A | ref|NM_015204|ens|ENST00000262042|ens|ENST00000408005|gb|AB023177 | ref|Homo sapiens thrombospondin, type I, domain containing 7A (THSD7A), mRNA [NM_015204 | 7,36635 |
| PLCXD3 | ref|NM_001005473|ens|ENST00000328457|ens|ENST00000377801|gb|AK124638 | ref|Homo sapiens phosphatidylinositol-specific phospholipase C, X domain containing 3 ( | 7,20937 |
| S100A6 | ref|NM_014624|ens|ENST00000368719|ens|ENST00000368720|gb|BT006965 | ref|Homo sapiens S100 calcium binding protein A6 (S100A6), mRNA [NM_014624] | 7,18559 |
| FRMD5 | ref|NM_032892|gb|AK090572|ens|ENST00000351529|gb|AK290013 | ref|Homo sapiens FERM domain containing 5 (FRMD5), transcript variant 2, mRNA [NM_03289 | 7,0911 |
| PRSS23 | ref|NM_007173|ens|ENST00000280258|gb|AK075362|thc|THC2469793 | ref|Homo sapiens protease, serine, 23 (PRSS23), mRNA [NM_007173] | 6,99328 |
| SEMA3E | ref|NM_012431|ens|ENST00000307792|gb|AK297209|gb|AK303925 | ref|Homo sapiens sema domain, immunoglobulin domain (Ig), short basic domain, secreted, | 6,96339 |
| DCBLD2 | ref|NM_080927|ens|ENST00000326840|gb|AK001362|gb|AB073146 | ref|Homo sapiens discoidin, CUB and LCCL domain containing 2 (DCBLD2), mRNA [NM_080927] | 6,80716 |
| SCD5 | ref|NM_001037582|ens|ENST00000319540|gb|CR601926|gb|CR621321 | ref|Homo sapiens stearoyl-CoA desaturase 5 (SCD5), transcript variant 1, mRNA [NM_00103 | 6,80386 |
| CSAG2 | ref|NM_004909|ref|NM_001080848|ref|NM_001129828|ref|NM_001129826 | ref|Homo sapiens CSAG family, member 2 (CSAG2), transcript variant 2, mRNA [NM_004909] | 6,77076 |
| SYNE1 | ref|NM_033071|ref|NM_182961|gb|AF535142|ens|ENST00000356820 | ref|Homo sapiens spectrin repeat containing, nuclear envelope 1 (SYNE1), transcript var | 6,59474 |
| CSAG2 | ref|NM_001080848|ref|NM_001129828|ref|NM_004909|ref|NM_001129826 | ref|Homo sapiens CSAG family, member 2 (CSAG2), transcript variant 1, mRNA [NM_00108084 | 6,53347 |
| GPR177 | ref|NM_024911|gb|AK074583|gb|BX648748|gb|BC110826 | ref|Homo sapiens G protein-coupled receptor 177 (GPR177), transcript variant 1, mRNA [N | 6,51223 |
| LARGE | ref|NM_004737|ref|NM_133642|gb|AB011181|ens|ENST00000402320 | ref|Homo sapiens like-glycosyltransferase (LARGE), transcript variant 1, mRNA [NM_00473 | 6,49921 |
| PSG8 | ref|NM_182707|ref|NM_001130167|ref|NM_001130168|ref|NM_021016 | ref|Homo sapiens pregnancy specific beta-1-glycoprotein 8 (PSG8), transcript variant 1, | 6,44797 |
| NTN4 | ref|NM_021229|gb|AF297711|ens|ENST00000343702|ens|ENST00000344911 | ref|Homo sapiens netrin 4 (NTN4), mRNA [NM_021229] | 6,31585 |
| PSG3 | ref|NM_021016|ref|NM_002781|ens|ENST00000327495|ens|ENST00000270059 | ref|Homo sapiens pregnancy specific beta-1-glycoprotein 3 (PSG3), mRNA [NM_021016] | 6,26831 |
| LPHN3 | ref|NM_015236|ens|ENST00000280009|ens|ENST00000295349|gb|AB018311 | ref|Homo sapiens latrophilin 3 (LPHN3), mRNA [NM_015236] | 6,2607 |
| GJA1 | ref|NM_000165|ens|ENST00000282561|gb|AK297761|gb|AK294670 | ref|Homo sapiens gap junction protein, alpha 1, 43kDa (GJA1), mRNA [NM_000165] | 6,23996 |
| TCEAL5 | ref|NM_001012979|ens|ENST00000372680|gb|CR596252|gb|CR622925 | ref|Homo sapiens transcription elongation factor A (SII)-like 5 (TCEAL5), mRNA [NM_0010 | 6,08793 |
| BC063022 | gb|BC063022|thc|THC2620648 | gb|Homo sapiens cDNA clone IMAGE:5246259, partial cds. [BC063022] | 6,0851 |
| JAM2 | ref|NM_021219|gb|AX772824|ens|ENST00000400532|gb|AK312708 | ref|Homo sapiens junctional adhesion molecule 2 (JAM2), mRNA [NM_021219] | 6,06832 |
| MAGEA6 | ref|NM_175868|ref|NM_005363|ref|NM_005362|ens|ENST00000393900 | ref|Homo sapiens melanoma antigen family A, 6 (MAGEA6), transcript variant 2, mRNA [NM_ | 6,06301 |
| AXL | ref|NM_021913|ref|NM_001699|ens|ENST00000301178|ens|ENST00000359092 | ref|Homo sapiens AXL receptor tyrosine kinase (AXL), transcript variant 1, mRNA [NM_021 | 6,04686 |
| TMEM185A | ref|NM_032508|ens|ENST00000316916|gb|AK128688|gb|AK096308 | ref|Homo sapiens transmembrane protein 185A (TMEM185A), mRNA [NM_032508] | 5,97337 |
| SEMA3E | ref|NM_012431|ens|ENST00000307792|gb|AB002329|gb|BC152458 | ref|Homo sapiens sema domain, immunoglobulin domain (Ig), short basic domain, secreted, | 5,92258 |
| GPX8 | ref|NM_001008397|ens|ENST00000296734|gb|AK298545|gb|AK074216 | ref|Homo sapiens glutathione peroxidase 8 (putative) (GPX8), mRNA [NM_001008397] | 5,91189 |
| LRRC8C | ref|NM_032270|ens|ENST00000370454|gb|AB081509|gb|BC113973 | ref|Homo sapiens leucine rich repeat containing 8 family, member C (LRRC8C), mRNA [NM_0 | 5,85422 |
| AK023096 | gb|AK023096|thc|THC2669129 | gb|Homo sapiens cDNA FLJ13034 fis, clone NT2RP3001232. [AK023096] | 5,70231 |
| ST3GAL1 | ref|NM_003033|ref|NM_173344|ens|ENST00000319914|ens|ENST00000395320 | ref|Homo sapiens ST3 beta-galactoside alpha-2,3-sialyltransferase 1 (ST3GAL1), transcri | 5,67077 |
| KCNK6 | ref|NM_004823|gb|BC004367|ens|ENST00000263372|gb|CR614493 | ref|Homo sapiens potassium channel, subfamily K, member 6 (KCNK6), mRNA [NM_004823] | 5,5405 |
| CXCL12 | ref|NM_199168|gb|DQ345517|ens|ENST00000343575|gb|CR615181 | ref|Homo sapiens chemokine (C-X-C motif) ligand 12 (stromal cell-derived factor 1) (CXC | 5,53741 |
| DCBLD2 | ref|NM_080927|ens|ENST00000404023|ens|ENST00000326857|ens|ENST00000326840 | ref|Homo sapiens discoidin, CUB and LCCL domain containing 2 (DCBLD2), mRNA [NM_080927] | 5,53349 |
| MAGEA4 | ref|NM_002362|ref|NM_001011548|ref|NM_001011549|ref|NM_001011550 | ref|Homo sapiens melanoma antigen family A, 4 (MAGEA4), transcript variant 2, mRNA [NM_ | 5,50742 |
| MAGEA12 | ref|NM_005367|ens|ENST00000357916|gb|CR541775|gb|AK314168 | ref|Homo sapiens melanoma antigen family A, 12 (MAGEA12), mRNA [NM_005367] | 5,47039 |
| MLLT3 | ref|NM_004529|gb|BC036089|ens|ENST00000380321|ens|ENST00000380323 | ref|Homo sapiens myeloid/lymphoid or mixed-lineage leukemia (trithorax homolog, Drosoph | 5,43936 |
| GPX8 | ref|NM_001008397|gb|AK022110|gb|AF086192|thc|THC2552685 | ref|Homo sapiens glutathione peroxidase 8 (putative) (GPX8), mRNA [NM_001008397] | 5,41835 |
| THC2667891 | thc|THC2667891 | Unknown | 5,36977 |
| EDN1 | ref|NM_001955|gb|CR605456|gb|AK226096|gb|BC009720 | ref|Homo sapiens endothelin 1 (EDN1), mRNA [NM_001955] | 5,35924 |
| TM4SF4 | ref|NM_004617|gb|AK055577|gb|U31449|gb|BE869034 | ref|Homo sapiens transmembrane 4 L six family member 4 (TM4SF4), mRNA [NM_004617] | 5,35051 |
| HKDC1 | ref|NM_025130|ens|ENST00000354624|gb|AX721041|gb|AK291024 | ref|Homo sapiens hexokinase domain containing 1 (HKDC1), mRNA [NM_025130] | 5,30417 |
| CST6 | ref|NM_001323|ens|ENST00000312134|gb|AK092391|gb|AX747517 | ref|Homo sapiens cystatin E/M (CST6), mRNA [NM_001323] | 5,24997 |
| SULT1B1 | gb|D89479 | gb|Homo sapiens mRNA for ST1B2, complete cds. [D89479] | 5,24138 |
| APBB1IP | ref|NM_019043|ens|ENST00000376236|gb|AK303597|gb|AB085852 | ref|Homo sapiens amyloid beta (A4) precursor protein-binding, family B, member 1 intera | 5,24097 |
| IGFALS | ref|NM_004970|ens|ENST00000215539|gb|AK315808|gb|AK292182 | ref|Homo sapiens insulin-like growth factor binding protein, acid labile subunit (IGFAL | 5,23611 |
| ITGA3 | ref|NM_002204|ref|NM_005501|gb|BC043917|ens|ENST00000320031 | ref|Homo sapiens integrin, alpha 3 (antigen CD49C, alpha 3 subunit of VLA-3 receptor) ( | 5,2081 |
| PCDH7 | ref|NM_002589|ens|ENST00000361762|gb|AB006755|thc|THC2480318 | ref|Homo sapiens protocadherin 7 (PCDH7), transcript variant a, mRNA [NM_002589] | 5,19398 |
| TGFB2 | ref|NM_001135599|ref|NM_003238|gb|AK021874|thc|THC2513197 | ref|Homo sapiens transforming growth factor, beta 2 (TGFB2), transcript variant 1, mRNA | 5,18153 |
| IL18 | ref|NM_001562|gb|AY266351|ens|ENST00000280357|gb|CR542001 | ref|Homo sapiens interleukin 18 (interferon-gamma-inducing factor) (IL18), mRNA [NM_001 | 5,11743 |
| MYOF | ref|NM_013451|ref|NM_133337|gb|BC052617|ens|ENST00000359263 | ref|Homo sapiens myoferlin (MYOF), transcript variant 1, mRNA [NM_013451] | 5,10836 |
| LOC284344 | gb|AK097672|thc|THC2515875 | gb|Homo sapiens cDNA FLJ40353 fis, clone TESTI2033520, weakly similar to BILIARY GLYCOP | 5,09482 |
| AREG | ref|NM_001657|ens|ENST00000395748|ens|ENST00000380846|gb|CR617114 | ref|Homo sapiens amphiregulin (AREG), mRNA [NM_001657] | 5,08162 |
| ANGPTL4 | ref|NM_139314|ref|NM_001039667|ens|ENST00000301455|ens|ENST00000393962 | ref|Homo sapiens angiopoietin-like 4 (ANGPTL4), transcript variant 1, mRNA [NM_139314] | 5,07963 |
| DNAH5 | ref|NM_001369|gb|AK026756|thc|THC2631581|thc|THC2478966 | ref|Homo sapiens dynein, axonemal, heavy chain 5 (DNAH5), mRNA [NM_001369] | 5,06417 |
| GCNT3 | ens|ENST00000267857|ens|ENST00000396065|gb|BC017032|gb|EF152283 | ens|Beta-1,3-galactosyl-O-glycosyl-glycoprotein beta-1,6-N-acetylglucosaminyltransferas | 5,04899 |
| CSAG1 | ref|NM_153478|ref|NM_001102576|ens|ENST00000361211|ens|ENST00000362050 | ref|Homo sapiens chondrosarcoma associated gene 1 (CSAG1), transcript variant a, mRNA [ | 4,94992 |
| KRT80 | ref|NM_182507|ref|NM_001081492|gb|BC065180|ens|ENST00000313234 | ref|Homo sapiens keratin 80 (KRT80), transcript variant 1, mRNA [NM_182507] | 4,88891 |
| NEK11 | ref|NM_024800|gb|AL833472|ens|ENST00000383366|gb|AK297300 | ref|Homo sapiens NIMA (never in mitosis gene a)- related kinase 11 (NEK11), transcript | 4,88621 |
| PCSK5 | ref|NM_006200|ens|ENST00000376752|gb|U49114|gb|BC012064 | ref|Homo sapiens proprotein convertase subtilisin/kexin type 5 (PCSK5), mRNA [NM_006200 | 4,88593 |
| ANXA13 | ref|NM_001003954|ref|NM_004306|ens|ENST00000262219|ens|ENST00000395546 | ref|Homo sapiens annexin A13 (ANXA13), transcript variant 2, mRNA [NM_001003954] | 4,82956 |
| AI379175 | gb|AI379175|gb|N32883|gb|AI874051|gb|BF899112 | gb|AI379175 tc66c08.x1 Soares_NhHMPu_S1 Homo sapiens cDNA clone IMAGE:2069582 3', mRNA | 4,77191 |
| PKHD1 | ref|NM_138694|gb|AY074797|ens|ENST00000371117|gb|BX538137 | ref|Homo sapiens polycystic kidney and hepatic disease 1 (autosomal recessive) (PKHD1), | 4,76454 |
| C9orf135 | ref|NM_001010940|ens|ENST00000377197|gb|BC150564|gb|BC136709 | ref|Homo sapiens chromosome 9 open reading frame 135 (C9orf135), mRNA [NM_001010940] | 4,68139 |
| DCBLD2 | ref|NM_080927|ens|ENST00000326857|ens|ENST00000326840|gb|AF387547 | ref|Homo sapiens discoidin, CUB and LCCL domain containing 2 (DCBLD2), mRNA [NM_080927] | 4,68042 |
| NPFFR2 | ref|NM_053036|ref|NM_004885|ens|ENST00000308744|ens|ENST00000358749 | ref|Homo sapiens neuropeptide FF receptor 2 (NPFFR2), transcript variant 2, mRNA [NM_05 | 4,65488 |
| FHOD3 | ref|NM_025135|gb|AK026370|ens|ENST00000257209|ens|ENST00000359247 | ref|Homo sapiens formin homology 2 domain containing 3 (FHOD3), mRNA [NM_025135] | 4,59717 |
| MT2A | ref|NM_005953|ens|ENST00000245185|gb|V00594|gb|CR625648 | ref|Homo sapiens metallothionein 2A (MT2A), mRNA [NM_005953] | 4,58856 |
| FHL2 | ref|NM_001039492|ref|NM_201555|ref|NM_001450|ref|NM_201557 | ref|Homo sapiens four and a half LIM domains 2 (FHL2), transcript variant 5, mRNA [NM_0 | 4,58155 |
| PRSS23 | ref|NM_007173|ens|ENST00000280258|gb|AK315562|gb|AM392532 | ref|Homo sapiens protease, serine, 23 (PRSS23), mRNA [NM_007173] | 4,57897 |
| AKR1B10 | ref|NM_020299|ens|ENST00000359579|gb|AF044961|gb|U37100 | ref|Homo sapiens aldo-keto reductase family 1, member B10 (aldose reductase) (AKR1B10), | 4,57176 |
| CYP3A5 | ref|NM_000777|gb|BX537676|ens|ENST00000222982|gb|CR620792 | ref|Homo sapiens cytochrome P450, family 3, subfamily A, polypeptide 5 (CYP3A5), mRNA [ | 4,5656 |
| FXYD2 | ref|NM_021603|ref|NM_001680|gb|AK096069|ens|ENST00000260287 | ref|Homo sapiens FXYD domain containing ion transport regulator 2 (FXYD2), transcript v | 4,54747 |
| KAL1 | ref|NM_000216|ens|ENST00000262648|gb|M97252|gb|S60088 | ref|Homo sapiens Kallmann syndrome 1 sequence (KAL1), mRNA [NM_000216] | 4,54473 |
| TMEM27 | ref|NM_020665|ens|ENST00000380342|gb|AF229179|gb|AY359060 | ref|Homo sapiens transmembrane protein 27 (TMEM27), mRNA [NM_020665] | 4,51923 |
| C14orf105 | ref|NM_018168|ens|ENST00000216445|gb|AK001512 | ref|Homo sapiens chromosome 14 open reading frame 105 (C14orf105), mRNA [NM_018168] | 4,51576 |
| MAP1B | ref|NM_005909|gb|CR933720|thc|THC2469907|thc|THC2519658 | ref|Homo sapiens microtubule-associated protein 1B (MAP1B), mRNA [NM_005909] | 4,51536 |
| TIMP1 | ref|NM_003254|gb|BC000866|ens|ENST00000218388|ens|ENST00000377013 | ref|Homo sapiens TIMP metallopeptidase inhibitor 1 (TIMP1), mRNA [NM_003254] | 4,50821 |
| MORC4 | ref|NM_024657|ref|NM_001085354|ens|ENST00000255495|ens|ENST00000355610 | ref|Homo sapiens MORC family CW-type zinc finger 4 (MORC4), transcript variant 1, mRNA | 4,4638 |
| LINCR | gb|BC012317|ens|ENST00000310865|gb|AL389981|thc|NP1167385 | gb|Homo sapiens likely ortholog of mouse lung-inducible Neutralized-related C3HC4 RING | 4,46134 |
| CCDC46 | ref|NM_145036|ref|NM_001037325|gb|AF458591|ens|ENST00000284400 | ref|Homo sapiens coiled-coil domain containing 46 (CCDC46), transcript variant 1, mRNA | 4,46038 |
| EFNA5 | ref|NM_001962|gb|AK025909|thc|THC2605802|thc|THC2613541 | ref|Homo sapiens ephrin-A5 (EFNA5), mRNA [NM_001962] | 4,43813 |
| LOC283658 | gb|AK055370|gb|AK095540|gb|AL833463|thc|THC2491609 | gb|Homo sapiens cDNA FLJ30808 fis, clone FEBRA2001383. [AK055370] | 4,43225 |
| SRPX2 | ref|NM_014467|ens|ENST00000373004|gb|AK075462|gb|AF060567 | ref|Homo sapiens sushi-repeat-containing protein, X-linked 2 (SRPX2), mRNA [NM_014467] | 4,40347 |
| PLK2 | ref|NM_006622|ens|ENST00000274289|gb|U85755|gb|BC013879 | ref|Homo sapiens polo-like kinase 2 (Drosophila) (PLK2), mRNA [NM_006622] | 4,39271 |
| MT2A | ref|NM_005953|ens|ENST00000245185|ens|ENST00000396160|gb|V00594 | ref|Homo sapiens metallothionein 2A (MT2A), mRNA [NM_005953] | 4,34364 |
| HS3ST5 | ref|NM_153612|gb|BC093911|gb|AK091074|ens|ENST00000312719 | ref|Homo sapiens heparan sulfate (glucosamine) 3-O-sulfotransferase 5 (HS3ST5), mRNA [N | 4,34007 |
| SGCB | ref|NM_000232|ens|ENST00000381431|gb|BT019433|gb|CR456810 | ref|Homo sapiens sarcoglycan, beta (43kDa dystrophin-associated glycoprotein) (SGCB), m | 4,33574 |
| ARMC3 | ref|NM_173081|gb|BC039312|ens|ENST00000376528|ens|ENST00000298032 | ref|Homo sapiens armadillo repeat containing 3 (ARMC3), mRNA [NM_173081] | 4,31101 |
| MT2A | ref|NM_005953|ens|ENST00000245185|gb|CR625648|gb|X97260 | ref|Homo sapiens metallothionein 2A (MT2A), mRNA [NM_005953] | 4,30432 |
| LOC550643 | gb|BX537532|gb|BC048131|gb|BC053960|gb|BC053962 | gb|Homo sapiens mRNA; cDNA DKFZp686L07201 (from clone DKFZp686L07201). [BX537532] | 4,24325 |
| SLC4A4 | ref|NM_003759|ref|NM_001134742|ref|NM_001098484|gb|AF157492 | ref|Homo sapiens solute carrier family 4, sodium bicarbonate cotransporter, member 4 (S | 4,23988 |
| IL1RAP | ref|NM_002182|gb|AF538730|ens|ENST00000072516|ens|ENST00000342550 | ref|Homo sapiens interleukin 1 receptor accessory protein (IL1RAP), transcript variant | 4,22636 |
| UCHL1 | ref|NM_004181|ens|ENST00000381762|ens|ENST00000284440|ens|ENST00000381760 | ref|Homo sapiens ubiquitin carboxyl-terminal esterase L1 (ubiquitin thiolesterase) (UCH | 4,21096 |
| CD59 | ref|NM_203330|ref|NM_001127223|ref|NM_203329|ref|NM_001127225 | ref|Homo sapiens CD59 molecule, complement regulatory protein (CD59), transcript varian | 4,20077 |
| ANXA13 | ref|NM_001003954|ref|NM_004306|ens|ENST00000262219|ens|ENST00000395546 | ref|Homo sapiens annexin A13 (ANXA13), transcript variant 2, mRNA [NM_001003954] | 4,14943 |
| LIMCH1 | ref|NM_014988|ref|NM_001112717|ref|NM_001112718|ref|NM_001112719 | ref|Homo sapiens LIM and calponin homology domains 1 (LIMCH1), transcript variant 1, mR | 4,14632 |
| RARB | ref|NM_000965|ref|NM_016152|gb|BC050415|ens|ENST00000330688 | ref|Homo sapiens retinoic acid receptor, beta (RARB), transcript variant 1, mRNA [NM_00 | 4,13321 |
| TMEM159 | ref|NM_020422|ens|ENST00000261388|ens|ENST00000233047|gb|AF070596 | ref|Homo sapiens transmembrane protein 159 (TMEM159), mRNA [NM_020422] | 4,11216 |
| TNFRSF19 | ref|NM_148957|ens|ENST00000248484|ens|ENST00000382263|gb|AF216077 | ref|Homo sapiens tumor necrosis factor receptor superfamily, member 19 (TNFRSF19), tran | 4,10925 |
| SYTL2 | ref|NM_206927|ref|NM_206928|ref|NM_032943|ref|NM_206930 | ref|Homo sapiens synaptotagmin-like 2 (SYTL2), transcript variant c, mRNA [NM_206927] | 4,09716 |
| THC2676797 | thc|THC2676797 | Unknown | 4,08933 |
| B3GNT3 | ref|NM_014256|ens|ENST00000318683|gb|AK075466|gb|AB049585 | ref|Homo sapiens UDP-GlcNAc:betaGal beta-1,3-N-acetylglucosaminyltransferase 3 (B3GNT3) | 4,08853 |
| LARP6 | ref|NM_018357|ens|ENST00000299213|gb|AK130092|gb|AF443828 | ref|Homo sapiens La ribonucleoprotein domain family, member 6 (LARP6), transcript varia | 4,06275 |
| MT1E | ref|NM_175617|gb|AF495759|ens|ENST00000306061|gb|BC131609 | ref|Homo sapiens metallothionein 1E (MT1E), mRNA [NM_175617] | 4,05386 |
| LIPH | ref|NM_139248|gb|BC064941|ens|ENST00000296252|gb|AK122651 | ref|Homo sapiens lipase, member H (LIPH), mRNA [NM_139248] | 4,02551 |
| CR616003 | gb|CR616003|gb|BC082758|thc|THC2511505|gb|BM687575 | gb|full-length cDNA clone CS0DM012YB14 of Fetal liver of Homo sapiens (human). [CR61600 | 4,01275 |
| UGT2A3 | ref|NM_024743|ref|NR_024010|gb|AY358727|ens|ENST00000251566 | ref|Homo sapiens UDP glucuronosyltransferase 2 family, polypeptide A3 (UGT2A3), mRNA [N | 3,99959 |
| CDH2 | ref|NM_001792|ens|ENST00000399380|ens|ENST00000269141|gb|S42303 | ref|Homo sapiens cadherin 2, type 1, N-cadherin (neuronal) (CDH2), mRNA [NM_001792] | 3,98037 |
| ARHGAP24 | ref|NM_001025616|ref|NM_031305|ref|NM_001042669|ens|ENST00000395184 | ref|Homo sapiens Rho GTPase activating protein 24 (ARHGAP24), transcript variant 1, mRN | 3,96348 |
| NCF2 | ref|NM_000433|ref|NM_001127651|ens|ENST00000392014|ens|ENST00000367535 | ref|Homo sapiens neutrophil cytosolic factor 2 (NCF2), transcript variant 1, mRNA [NM_0 | 3,96006 |
| CSTF2T | ref|NM_015235|ens|ENST00000331173|gb|AY130299|gb|AK222574 | ref|Homo sapiens cleavage stimulation factor, 3' pre-RNA, subunit 2, 64kDa, tau variant | 3,95191 |
| CDH6 | ref|NM_004932|ens|ENST00000265071|gb|D31784|gb|CA314809 | ref|Homo sapiens cadherin 6, type 2, K-cadherin (fetal kidney) (CDH6), mRNA [NM_004932] | 3,93648 |
| STAC | ref|NM_003149|ens|ENST00000273183|gb|BC020221|gb|AK056893 | ref|Homo sapiens SH3 and cysteine rich domain (STAC), mRNA [NM_003149] | 3,89894 |
| DYRK3 | ref|NM_001004023|ref|NM_003582|ens|ENST00000367108|ens|ENST00000367109 | ref|Homo sapiens dual-specificity tyrosine-(Y)-phosphorylation regulated kinase 3 (DYRK | 3,88961 |
| BM768581 | gb|BM768581|thc|THC2742482 | gb|K-EST0051582 S14K402 Homo sapiens cDNA clone S14K402-6-G10 5', mRNA sequence [BM7685 | 3,88932 |
| MT1X | ref|NM_005952|ref|NM_005951|ref|NM_005946|gb|BC032131 | ref|Homo sapiens metallothionein 1X (MT1X), mRNA [NM_005952] | 3,83711 |
| MT1L | ref|NR_001447|gb|X97261|gb|BC070351|gb|AF348998 | ref|Homo sapiens metallothionein 1L (gene/pseudogene) (MT1L), non-coding RNA [NR_001447 | 3,83652 |
| MT1G | ref|NM_005950|gb|BC020757|ens|ENST00000379811|ens|ENST00000245183 | ref|Homo sapiens metallothionein 1G (MT1G), mRNA [NM_005950] | 3,81021 |
| CDKN2B | ref|NM_078487|ref|NM_004936|ens|ENST00000276925|gb|BC014469 | ref|Homo sapiens cyclin-dependent kinase inhibitor 2B (p15, inhibits CDK4) (CDKN2B), tr | 3,80888 |
| CITED4 | ref|NM_133467|ens|ENST00000372638|gb|BC035496|gb|BC052559 | ref|Homo sapiens Cbp/p300-interacting transactivator, with Glu/Asp-rich carboxy-termina | 3,80126 |
| DSEL | ref|NM_032160|ens|ENST00000397964|ens|ENST00000310045|gb|AF480435 | ref|Homo sapiens dermatan sulfate epimerase-like (DSEL), mRNA [NM_032160] | 3,79443 |
| TGFB2 | ref|NM_003238|ref|NM_001135599|gb|M19154|ens|ENST00000366929 | ref|Homo sapiens transforming growth factor, beta 2 (TGFB2), transcript variant 2, mRNA | 3,79391 |
| CXCL12 | ref|NM_000609|gb|BX647204|ens|ENST00000374429|gb|FB357022 | ref|Homo sapiens chemokine (C-X-C motif) ligand 12 (stromal cell-derived factor 1) (CXC | 3,79255 |
| CACNG6 | ref|NM_145814|ref|NM_145815|ref|NM_031897|ens|ENST00000252729 | ref|Homo sapiens calcium channel, voltage-dependent, gamma subunit 6 (CACNG6), transcri | 3,7912 |
| ANKRD18A | gb|AB095935|ens|ENST00000399703|ens|ENST00000313339|ens|ENST00000357072 | gb|Homo sapiens mRNA for KIAA2015 protein. [AB095935] | 3,78453 |
| NLGN1 | ref|NM_014932|ens|ENST00000361589|gb|AB028993|thc|THC2473172 | ref|Homo sapiens neuroligin 1 (NLGN1), mRNA [NM_014932] | 3,77921 |
| SPEG | gb|AK055387|gb|BC006346|ens|ENST00000396698|ens|ENST00000396695 | gb|Homo sapiens cDNA FLJ30825 fis, clone FEBRA2001706, highly similar to Human APEG-1 m | 3,77845 |
| ARSJ | ref|NM_024590|ens|ENST00000315366|gb|BC032010|gb|BC151213 | ref|Homo sapiens arylsulfatase family, member J (ARSJ), mRNA [NM_024590] | 3,76793 |
| ARSI | ref|NM_001012301|gb|BC111002|ens|ENST00000328668|gb|CR621346 | ref|Homo sapiens arylsulfatase family, member I (ARSI), mRNA [NM_001012301] | 3,72167 |
| MAF | gb|AF055376 | gb|Homo sapiens short form transcription factor C-MAF (c-maf) mRNA, complete cds. [AF05 | 3,71483 |
| SPNS2 | ref|NM_001124758|gb|BC041772|ens|ENST00000329078|gb|BC065221 | ref|Homo sapiens spinster homolog 2 (Drosophila) (SPNS2), mRNA [NM_001124758] | 3,71451 |
| COL4A4 | ref|NM_000092|ens|ENST00000329662|ens|ENST00000396625|ens|ENST00000329438 | ref|Homo sapiens collagen, type IV, alpha 4 (COL4A4), mRNA [NM_000092] | 3,71263 |
| BC013171 | gb|BC013171|ens|ENST00000361201|thc|THC2489728 | gb|Homo sapiens cDNA clone IMAGE:4344401, **** WARNING: chimeric clone ****. [BC013171] | 3,69979 |
| LEPREL1 | ref|NM_018192|ref|NM_001134418|gb|BC005029|ens|ENST00000319332 | ref|Homo sapiens leprecan-like 1 (LEPREL1), transcript variant 1, mRNA [NM_018192] | 3,68415 |
| MT1X | ref|NM_005952|gb|BC032131|ens|ENST00000290704|ens|ENST00000394485 | ref|Homo sapiens metallothionein 1X (MT1X), mRNA [NM_005952] | 3,67567 |
| LOC124220 | ref|NM_145252|gb|BC009722|ens|ENST00000293983|ens|ENST00000382280 | ref|Homo sapiens similar to common salivary protein 1 (LOC124220), mRNA [NM_145252] | 3,64587 |
| CITED4 | ref|NM_133467|ens|ENST00000372638|gb|BC035496|gb|BC052559 | ref|Homo sapiens Cbp/p300-interacting transactivator, with Glu/Asp-rich carboxy-termina | 3,62275 |
| MGC2848 | gb|BC007984|thc|THC2685241 | gb|Homo sapiens hypothetical protein MGC2848, mRNA (cDNA clone IMAGE:2967248), partial | 3,61888 |
| MT1H | ref|NM_005951|ref|NM_005952|ref|NR_001447|gb|BC032131 | ref|Homo sapiens metallothionein 1H (MT1H), mRNA [NM_005951] | 3,61141 |
| TMEM17 | ref|NM_198276|ens|ENST00000335390|gb|AK091902|gb|AX747245 | ref|Homo sapiens transmembrane protein 17 (TMEM17), mRNA [NM_198276] | 3,59739 |
| THC2537219 | thc|THC2537219|thc|THC2560008 | thc|Q6ATL9_ORYSA (Q6ATL9) Expressed protein, partial (13%) [THC2537219] | 3,58684 |
| APBB1IP | ref|NM_019043|gb|AK000812|ens|ENST00000335513|ens|ENST00000376236 | ref|Homo sapiens amyloid beta (A4) precursor protein-binding, family B, member 1 intera | 3,57528 |
| GULP1 | ref|NM_016315|ens|ENST00000359135|ens|ENST00000409580|gb|BC068525 | ref|Homo sapiens GULP, engulfment adaptor PTB domain containing 1 (GULP1), mRNA [NM_016 | 3,55594 |
| GCNT3 | ref|NM_004751|ens|ENST00000267857|gb|AK312852|gb|AF038650 | ref|Homo sapiens glucosaminyl (N-acetyl) transferase 3, mucin type (GCNT3), mRNA [NM_00 | 3,54068 |
| VTCN1 | ref|NM_024626|gb|BX648021|ens|ENST00000256654|ens|ENST00000369458 | ref|Homo sapiens V-set domain containing T cell activation inhibitor 1 (VTCN1), mRNA [N | 3,53721 |
| BCAS1 | ref|NM_003657|ens|ENST00000371440|ens|ENST00000395961|ens|ENST00000371435 | ref|Homo sapiens breast carcinoma amplified sequence 1 (BCAS1), mRNA [NM_003657] | 3,532 |
| LRRN1 | ref|NM_020873|ens|ENST00000319331|gb|AB040930|gb|BC034947 | ref|Homo sapiens leucine rich repeat neuronal 1 (LRRN1), mRNA [NM_020873] | 3,5237 |
| PLXDC2 | ref|NM_032812|gb|BC012885|gb|AF378757|ens|ENST00000377252 | ref|Homo sapiens plexin domain containing 2 (PLXDC2), mRNA [NM_032812] | 3,50653 |
| EIF4E3 | ref|NM_173359|ref|NM_001134649|ref|NM_001134650|ref|NM_001134651 | ref|Homo sapiens eukaryotic translation initiation factor 4E family member 3 (EIF4E3), | 3,48368 |
| TAF15 | ref|NM_139215|ref|NM_003487|gb|BC046099|ens|ENST00000394533 | ref|Homo sapiens TAF15 RNA polymerase II, TATA box binding protein (TBP)-associated fac | 3,46978 |
| ABCB1 | ref|NM_000927|gb|AF016535|ens|ENST00000265724|gb|EU852583 | ref|Homo sapiens ATP-binding cassette, sub-family B (MDR/TAP), member 1 (ABCB1), mRNA [ | 3,46205 |
| EHD2 | ref|NM_014601|gb|AK074701|ens|ENST00000263277|gb|CR609202 | ref|Homo sapiens EH-domain containing 2 (EHD2), mRNA [NM_014601] | 3,45997 |
| BDNF | ref|NM_170735|ref|NM_170731|ref|NM_170732|ref|NM_170733 | ref|Homo sapiens brain-derived neurotrophic factor (BDNF), transcript variant 1, mRNA [ | 3,44355 |
| THC2659853 | thc|THC2659853 | Unknown | 3,42858 |
| CRYBG3 | ref|NM_153605|gb|AK098569|gb|AF451988|gb|BX647079 | ref|Homo sapiens beta-gamma crystallin domain containing 3 (CRYBG3), mRNA [NM_153605] | 3,42775 |
| PKHD1 | ref|NM_138694|gb|AY074797|ens|ENST00000371117|gb|AK091971 | ref|Homo sapiens polycystic kidney and hepatic disease 1 (autosomal recessive) (PKHD1), | 3,42207 |
| AL359052 | gb|AL359052|gb|AK075136|thc|THC2602031 | gb|Homo sapiens mRNA full length insert cDNA clone EUROIMAGE 1968422. [AL359052] | 3,41719 |
| IGFBP1 | ref|NM_000596|gb|CR595377|ens|ENST00000275525|gb|CR622795 | ref|Homo sapiens insulin-like growth factor binding protein 1 (IGFBP1), mRNA [NM_000596 | 3,39993 |
| PRSS2 | ref|NM_002770|ref|NM_007343|ref|NM_002771|ref|NR_001296 | ref|Homo sapiens protease, serine, 2 (trypsin 2) (PRSS2), transcript variant 1, mRNA [N | 3,39838 |
| HS3ST1 | ref|NM_005114|ens|ENST00000002596|gb|AF019386|gb|BC057803 | ref|Homo sapiens heparan sulfate (glucosamine) 3-O-sulfotransferase 1 (HS3ST1), mRNA [N | 3,39476 |
| SMOC1 | ref|NM_001034852|ref|NM_022137|ens|ENST00000361956|gb|CR610837 | ref|Homo sapiens SPARC related modular calcium binding 1 (SMOC1), transcript variant 1, | 3,38869 |
| MT1H | ref|NM_005951|ref|NM_176870|ens|ENST00000294916|ens|ENST00000332374 | ref|Homo sapiens metallothionein 1H (MT1H), mRNA [NM_005951] | 3,38074 |
| GLIPR2 | ref|NM_022343|ens|ENST00000377960|ens|ENST00000396613|ens|ENST00000396614 | ref|Homo sapiens GLI pathogenesis-related 2 (GLIPR2), mRNA [NM_022343] | 3,3693 |
| LOC129293 | ref|NM_001080824|ens|ENST00000335459|ens|ENST00000409520|gb|AK128325 | ref|Homo sapiens hypothetical protein LOC129293 (LOC129293), mRNA [NM_001080824] | 3,35867 |
| C8orf47 | ref|NM_173549|ens|ENST00000396066|ens|ENST00000318528|gb|CR621279 | ref|Homo sapiens chromosome 8 open reading frame 47 (C8orf47), mRNA [NM_173549] | 3,35159 |
| MYOF | ref|NM_013451|ref|NM_133337|gb|BC052617|ens|ENST00000359263 | ref|Homo sapiens myoferlin (MYOF), transcript variant 1, mRNA [NM_013451] | 3,34843 |
| OLFML2A | ref|NM_182487|gb|BC054001|ens|ENST00000373580|ens|ENST00000331715 | ref|Homo sapiens olfactomedin-like 2A (OLFML2A), mRNA [NM_182487] | 3,33548 |
| MARK1 | ref|NM_018650|gb|AF154845|ens|ENST00000366917|ens|ENST00000402574 | ref|Homo sapiens MAP/microtubule affinity-regulating kinase 1 (MARK1), mRNA [NM_018650] | 3,33211 |
| MAP1B | ref|NM_005909|gb|CR595522|gb|CR613305|gb|AK055112 | ref|Homo sapiens microtubule-associated protein 1B (MAP1B), mRNA [NM_005909] | 3,33165 |
| HKDC1 | ref|NM_025130|gb|BC110504|ens|ENST00000395086|ens|ENST00000354624 | ref|Homo sapiens hexokinase domain containing 1 (HKDC1), mRNA [NM_025130] | 3,31564 |
| SPP1 | ref|NM_001040058|ref|NM_001040060|ref|NM_000582|gb|AK075463 | ref|Homo sapiens secreted phosphoprotein 1 (SPP1), transcript variant 1, mRNA [NM_00104 | 3,30016 |
| AP1S1 | ref|NM_001283|gb|CR599373|ens|ENST00000337619|gb|BT006779 | ref|Homo sapiens adaptor-related protein complex 1, sigma 1 subunit (AP1S1), mRNA [NM_0 | 3,29862 |
| M31157 | gb|M31157|thc|THC2659026 | gb|Human parathyroid hormone-like peptide mRNA, 3' end. [M31157] | 3,29294 |
| NAP5 | ref|NM_207363|ref|NM_207481|gb|AK124659|ens|ENST00000317721 | ref|Homo sapiens Nck-associated protein 5 (NAP5), transcript variant 1, mRNA [NM_207363 | 3,28357 |
| FLJ22167 | ref|NM_001077419|ref|NM_001077416|ref|NM_001077418|gb|BC010609 | ref|Homo sapiens hypothetical protein FLJ22167 (FLJ22167), transcript variant 3, mRNA [ | 3,27769 |
| ARHGEF18 | ref|NM_015318|ref|NM_001130955|gb|AK127045|gb|BC014994 | ref|Homo sapiens rho/rac guanine nucleotide exchange factor (GEF) 18 (ARHGEF18), transc | 3,27119 |
| PRSS2 | ref|NM_002770|ref|NR_001296|gb|BC103998|ens|ENST00000361585 | ref|Homo sapiens protease, serine, 2 (trypsin 2) (PRSS2), transcript variant 1, mRNA [N | 3,26918 |
| CD109 | ref|NM_133493|gb|AK095888|ens|ENST00000287097|gb|AY374442 | ref|Homo sapiens CD109 molecule (CD109), mRNA [NM_133493] | 3,26853 |
| MT1B | ref|NM_005947|ens|ENST00000334346|gb|BC069421|gb|AF349000 | ref|Homo sapiens metallothionein 1B (MT1B), mRNA [NM_005947] | 3,26849 |
| BCL2A1 | ref|NM_004049|ref|NM_001114735|gb|AY234180|ens|ENST00000267953 | ref|Homo sapiens BCL2-related protein A1 (BCL2A1), transcript variant 1, mRNA [NM_00404 | 3,26847 |
| CLDN1 | ref|NM_021101|ens|ENST00000295522|thc|THC2509366 | ref|Homo sapiens claudin 1 (CLDN1), mRNA [NM_021101] | 3,26141 |
| IL1RAP | ref|NM_002182|gb|AF538730|ens|ENST00000072516|ens|ENST00000342550 | ref|Homo sapiens interleukin 1 receptor accessory protein (IL1RAP), transcript variant | 3,26104 |
| PTHLH | ref|NM_198965|ref|NM_198966|ref|NM_002820|ref|NM_198964 | ref|Homo sapiens parathyroid hormone-like hormone (PTHLH), transcript variant 1, mRNA [ | 3,2566 |
| CHST9 | ref|NM_031422|gb|BC025764|gb|AY358488|ens|ENST00000284224 | ref|Homo sapiens carbohydrate (N-acetylgalactosamine 4-0) sulfotransferase 9 (CHST9), m | 3,2462 |
| INPP4B | ref|NM_003866|ref|NM_001101669|ens|ENST00000262992|gb|BC072447 | ref|Homo sapiens inositol polyphosphate-4-phosphatase, type II, 105kDa (INPP4B), transc | 3,24446 |
| NDRG1 | ref|NM_006096|ref|NM_001135242|gb|AK096562|gb|AK095649 | ref|Homo sapiens N-myc downstream regulated 1 (NDRG1), transcript variant 2, mRNA [NM_0 | 3,23778 |
| THC2507433 | thc|THC2507433|thc|THC2487497 | thc|Q5TD55_HUMAN (Q5TD55) OTTHUMP00000031270 (Fragment), partial (71%) [THC2507433] | 3,218 |
| PGM2L1 | ref|NM_173582|ens|ENST00000298198|gb|CR606675|gb|AK056591 | ref|Homo sapiens phosphoglucomutase 2-like 1 (PGM2L1), mRNA [NM_173582] | 3,21648 |
| SLC22A3 | ref|NM_021977|ens|ENST00000392145|ens|ENST00000275300|gb|CR619195 | ref|Homo sapiens solute carrier family 22 (extraneuronal monoamine transporter), member | 3,20402 |
| DYRK3 | ref|NM_001004023|ref|NM_003582|ens|ENST00000367106|ens|ENST00000367108 | ref|Homo sapiens dual-specificity tyrosine-(Y)-phosphorylation regulated kinase 3 (DYRK | 3,20338 |
| FEZ1 | ref|NM_005103|ens|ENST00000392709|ens|ENST00000278919|gb|CR541954 | ref|Homo sapiens fasciculation and elongation protein zeta 1 (zygin I) (FEZ1), transcri | 3,20101 |
| ACAA2 | ref|NM_006111|ens|ENST00000285093|ens|ENST00000398549|gb|CR599578 | ref|Homo sapiens acetyl-Coenzyme A acyltransferase 2 (ACAA2), nuclear gene encoding mit | 3,18895 |
| LOC284422 | ref|NM_001136503|ens|ENST00000211092|ens|ENST00000215531|gb|AF161441 | ref|Homo sapiens similar to HSPC323 (LOC284422), mRNA [NM_001136503] | 3,18423 |
| CYP3A4 | ref|NM_017460|ens|ENST00000336411|ens|ENST00000354593|gb|AY606313 | ref|Homo sapiens cytochrome P450, family 3, subfamily A, polypeptide 4 (CYP3A4), mRNA [ | 3,18219 |
| BC013657 | gb|BC013657 | gb|Homo sapiens cDNA clone IMAGE:4152983, partial cds. [BC013657] | 3,18162 |
| LYPD6B | ref|NM_177964|gb|BC018203|ens|ENST00000409876|ens|ENST00000280115 | ref|Homo sapiens LY6/PLAUR domain containing 6B (LYPD6B), mRNA [NM_177964] | 3,17977 |
| C4orf19 | ref|NM_001104629|ref|NM_018302|thc|THC2668561 | ref|Homo sapiens chromosome 4 open reading frame 19 (C4orf19), transcript variant 1, mR | 3,17775 |
| NFIB | ref|NM_005596|ens|ENST00000397581|ens|ENST00000397579|ens|ENST00000380959 | ref|Homo sapiens nuclear factor I/B (NFIB), mRNA [NM_005596] | 3,16164 |
| HS3ST5 | ref|NM_153612|gb|AK091074|gb|AK126930|gb|AX746766 | ref|Homo sapiens heparan sulfate (glucosamine) 3-O-sulfotransferase 5 (HS3ST5), mRNA [N | 3,16124 |
| UGCG | ref|NM_003358|ens|ENST00000374279|gb|AK314847|gb|BC038711 | ref|Homo sapiens UDP-glucose ceramide glucosyltransferase (UGCG), mRNA [NM_003358] | 3,15911 |
| THC2640099 | thc|THC2640099 | Unknown | 3,15445 |
| UGT2B17 | ref|NM_001077|ens|ENST00000317746|gb|U59209|thc|THC2483859 | ref|Homo sapiens UDP glucuronosyltransferase 2 family, polypeptide B17 (UGT2B17), mRNA | 3,15158 |
| KLRC3 | ref|NM_007333|ref|NM_002261|ref|NM_002260|ref|NM_002259 | ref|Homo sapiens killer cell lectin-like receptor subfamily C, member 3 (KLRC3), transc | 3,14823 |
| FAM148C | ref|NM_001136263|gb|CR616939|gb|AB075837|gb|AL834341 | ref|Homo sapiens family with sequence similarity 148, member C (FAM148C), mRNA [NM_0011 | 3,14083 |
| AI824599 | gb|AI824599|gb|AI580412|gb|AI401657|gb|AA975420 | gb|AI824599 wc18g09.x1 NCI_CGAP_Pr28 Homo sapiens cDNA clone IMAGE:2315584 3', mRNA seq | 3,13742 |
| THC2684461 | thc|THC2684461|gb|AI261357 | thc|Q7Z2R6_HUMAN (Q7Z2R6) MSTP132, partial (34%) [THC2684461] | 3,13154 |
| COLEC12 | ref|NM_130386|ens|ENST00000400256|ens|ENST00000344226|gb|AB052103 | ref|Homo sapiens collectin sub-family member 12 (COLEC12), mRNA [NM_130386] | 3,12354 |
| AF086529 | gb|AF086529 | gb|Homo sapiens full length insert cDNA clone ZE05E03. [AF086529] | 3,12346 |
| TRIM7 | ref|NM_033342|ens|ENST00000334421|gb|AF220032|gb|BC080553 | ref|Homo sapiens tripartite motif-containing 7 (TRIM7), transcript variant 6, mRNA [NM_ | 3,11537 |
| LONRF2 | ref|NM_198461|ens|ENST00000393435|ens|ENST00000393437|gb|CR590043 | ref|Homo sapiens LON peptidase N-terminal domain and ring finger 2 (LONRF2), mRNA [NM_1 | 3,11016 |
| EMP2 | ref|NM_001424|ens|ENST00000320378|gb|AK096403|gb|AK127600 | ref|Homo sapiens epithelial membrane protein 2 (EMP2), mRNA [NM_001424] | 3,10873 |
| CTBP2 | ref|NM_022802|ref|NM_001083914|ref|NM_001329|ens|ENST00000337195 | ref|Homo sapiens C-terminal binding protein 2 (CTBP2), transcript variant 2, mRNA [NM_0 | 3,09509 |
| HAS3 | ref|NM_005329|ens|ENST00000306560|gb|AF232772|thc|THC2472843 | ref|Homo sapiens hyaluronan synthase 3 (HAS3), transcript variant 1, mRNA [NM_005329] | 3,0914 |
| CCDC103 | ens|ENST00000357776|ens|ENST00000331733|gb|AK023156|gb|AK091530 | ens|Coiled-coil domain-containing protein 103 [Source:UniProtKB/Swiss-Prot;Acc:Q8IW40] | 3,09046 |
| FOXJ1 | ref|NM_001454|ens|ENST00000322957|gb|X99349|gb|BC046460 | ref|Homo sapiens forkhead box J1 (FOXJ1), mRNA [NM_001454] | 3,08777 |
| HAS2 | ref|NM_005328|ens|ENST00000303924|gb|BC109071|gb|BC109072 | ref|Homo sapiens hyaluronan synthase 2 (HAS2), mRNA [NM_005328] | 3,08436 |
| IRS2 | ref|NM_003749|ens|ENST00000375856|gb|CR615148|gb|AF073310 | ref|Homo sapiens insulin receptor substrate 2 (IRS2), mRNA [NM_003749] | 3,08294 |
| TMSL3 | ref|NM_183049|ref|NM_021109|ens|ENST00000380633|ens|ENST00000380638 | ref|Homo sapiens thymosin-like 3 (TMSL3), mRNA [NM_183049] | 3,07804 |
| MLLT3 | ref|NM_004529|ens|ENST00000380323|ens|ENST00000380338|ens|ENST00000355930 | ref|Homo sapiens myeloid/lymphoid or mixed-lineage leukemia (trithorax homolog, Drosoph | 3,07724 |
| RASSF8 | ref|NM_007211|ens|ENST00000282884|ens|ENST00000381352|gb|U82396 | ref|Homo sapiens Ras association (RalGDS/AF-6) domain family (N-terminal) member 8 (RAS | 3,07331 |
| ZNF618 | ref|NM_133374|gb|BC036039|ens|ENST00000374126|ens|ENST00000374124 | ref|Homo sapiens zinc finger protein 618 (ZNF618), mRNA [NM_133374] | 3,06641 |
| BP398053 | gb|BP398053|thc|THC2640652 | gb|BP398053 Homo sapiens pancreatic islet Homo sapiens cDNA clone htp-09-88 3', mRNA se | 3,06396 |
| DKK3 | ref|NM_015881|ref|NM_013253|ref|NM_001018057|ens|ENST00000396505 | ref|Homo sapiens dickkopf homolog 3 (Xenopus laevis) (DKK3), transcript variant 1, mRNA | 3,06266 |
| NBEA | ref|NM_015678|gb|AL137748|gb|AF467288|ens|ENST00000402346 | ref|Homo sapiens neurobeachin (NBEA), mRNA [NM_015678] | 3,05925 |
| COL4A6 | ref|NM_033641|ref|NM_001847|ens|ENST00000394872|ens|ENST00000334504 | ref|Homo sapiens collagen, type IV, alpha 6 (COL4A6), transcript variant B, mRNA [NM_03 | 3,05767 |
| IL11 | ref|NM_000641|ens|ENST00000264563|gb|E04896|gb|E08229 | ref|Homo sapiens interleukin 11 (IL11), mRNA [NM_000641] | 3,05728 |
| FBN1 | ref|NM_000138|ens|ENST00000389087|ens|ENST00000316623|gb|BC146854 | ref|Homo sapiens fibrillin 1 (FBN1), mRNA [NM_000138] | 3,05625 |
| SLC39A7 | ref|NM_006979|ref|NM_001077516|gb|BC000645|ens|ENST00000404800 | ref|Homo sapiens solute carrier family 39 (zinc transporter), member 7 (SLC39A7), trans | 3,054 |
| KRT17P3 | ref|XR_019109|ref|XR_015626|ref|XR_038024 | ref|PREDICTED: Homo sapiens misc_RNA (KRT17P3), miscRNA [XR_019109] | 3,05291 |
| FAM59A | ref|NM_022751|ens|ENST00000269209|ens|ENST00000399218|gb|AK297430 | ref|Homo sapiens family with sequence similarity 59, member A (FAM59A), mRNA [NM_022751 | 3,04525 |
| BM968605 | gb|BM968605|gb|BU678091 | gb|BM968605 UI-CF-DU1-aap-l-19-0-UI.s1 UI-CF-DU1 Homo sapiens cDNA clone UI-CF-DU1-aap- | 3,04013 |
| ARL14 | ref|NM_025047|ens|ENST00000320767|gb|AK026248|gb|BC034354 | ref|Homo sapiens ADP-ribosylation factor-like 14 (ARL14), mRNA [NM_025047] | 3,039 |
| AA554330 | gb|AA554330|gb|AA516273|gb|AI262132|gb|AI522121 | gb|nl03d08.s1 NCI_CGAP_Co3 Homo sapiens cDNA clone IMAGE:1029231 3', mRNA sequence [AA5 | 3,03244 |
| MITF | ref|NM_198159|ref|NM_006722|ref|NM_198177|ref|NM_198178 | ref|Homo sapiens microphthalmia-associated transcription factor (MITF), transcript vari | 3,02966 |
| KIAA0802 | ref|NM_015210|gb|BC040542|ens|ENST00000306329|gb|AB018345 | ref|Homo sapiens KIAA0802 (KIAA0802), mRNA [NM_015210] | 3,0265 |
| STYK1 | ref|NM_018423|ens|ENST00000075503|gb|AF251059|gb|AL353940 | ref|Homo sapiens serine/threonine/tyrosine kinase 1 (STYK1), mRNA [NM_018423] | 3,02386 |
| LRRIQ1 | ref|NM_032165|ref|NM_001079910|gb|BC005399|ens|ENST00000256007 | ref|Homo sapiens leucine-rich repeats and IQ motif containing 1 (LRRIQ1), transcript va | 3,01989 |
| CBLN2 | ref|NM_182511|ens|ENST00000269503|gb|AK125422|gb|AY359074 | ref|Homo sapiens cerebellin 2 precursor (CBLN2), mRNA [NM_182511] | 3,00169 |
|  |  |  |  |
| **B. Downregulated genes** | | | |
| RNF144B | ref|NM_182757|ens|ENST00000259939|gb|AB076367|gb|BC063311 | ref|Homo sapiens ring finger protein 144B (RNF144B), mRNA [NM_182757] | -3,02742 |
| CYP39A1 | ref|NM_016593|ens|ENST00000275016|gb|DQ892889|gb|EU176719 | ref|Homo sapiens cytochrome P450, family 39, subfamily A, polypeptide 1 (CYP39A1), mRNA | -3,03289 |
| AW386276 | gb|AW386276|gb|BE708730|gb|AA705426|gb|AA678022 | gb|AW386276 CM2-PT0015-141299-055-d11 PT0015 Homo sapiens cDNA, mRNA sequence [AW386276 | -3,04713 |
| FGF19 | ref|NM_005117|ens|ENST00000294312|gb|CR610453|gb|AY358302 | ref|Homo sapiens fibroblast growth factor 19 (FGF19), mRNA [NM_005117] | -3,06913 |
| MBL2 | ref|NM_000242|ens|ENST00000373968|gb|X15422|thc|THC2615144 | ref|Homo sapiens mannose-binding lectin (protein C) 2, soluble (opsonic defect) (MBL2), | -3,07421 |
| GATA3 | ref|NM_001002295|ref|NM_002051|gb|BC006793|ens|ENST00000379328 | ref|Homo sapiens GATA binding protein 3 (GATA3), transcript variant 1, mRNA [NM_0010022 | -3,10974 |
| SLC25A27 | ref|NM_004277|ens|ENST00000355073|ens|ENST00000371347|gb|AY358711 | ref|Homo sapiens solute carrier family 25, member 27 (SLC25A27), nuclear gene encoding | -3,11214 |
| PAQR9 | ref|NM_198504|gb|AK123932|gb|BC030124|thc|THC2613466 | ref|Homo sapiens progestin and adipoQ receptor family member IX (PAQR9), mRNA [NM_19850 | -3,11484 |
| BE904671 | gb|BE904671 | gb|601498784F1 NIH_MGC_70 Homo sapiens cDNA clone IMAGE:3900717 5', mRNA sequence [BE90 | -3,1335 |
| MCTP1 | ref|NM_024717|ref|NM_001002796|gb|AK025997|gb|AK091330 | ref|Homo sapiens multiple C2 domains, transmembrane 1 (MCTP1), transcript variant L, mR | -3,14609 |
| AFP | ref|NM_001134|ens|ENST00000395792|ens|ENST00000226359|gb|AK314817 | ref|Homo sapiens alpha-fetoprotein (AFP), mRNA [NM_001134] | -3,14909 |
| KNG1 | ref|NM_000893|ref|NM_001102416|ens|ENST00000287611|ens|ENST00000265023 | ref|Homo sapiens kininogen 1 (KNG1), transcript variant 2, mRNA [NM_000893] | -3,16037 |
| PRG4 | ref|NM_005807|ref|NM_001127708|ref|NM_001127709|ref|NM_001127710 | ref|Homo sapiens proteoglycan 4 (PRG4), transcript variant A, mRNA [NM_005807] | -3,17868 |
| UGT2B4 | ref|NM_021139|ens|ENST00000305107|gb|AK292748|gb|AJ005162 | ref|Homo sapiens UDP glucuronosyltransferase 2 family, polypeptide B4 (UGT2B4), mRNA [N | -3,18081 |
| KCNS3 | ref|NM_002252|ens|ENST00000403915|ens|ENST00000304101|gb|AK225833 | ref|Homo sapiens potassium voltage-gated channel, delayed-rectifier, subfamily S, membe | -3,19444 |
| FGG | ref|NM_000509|ref|NM_021870|gb|CR597461|gb|CR606086 | ref|Homo sapiens fibrinogen gamma chain (FGG), transcript variant gamma-A, mRNA [NM_000 | -3,19718 |
| THC2730628 | thc|THC2730628 | thc|Q9X6G7_STRPY (Q9X6G7) Serum opacity factor precursor (Fragment), partial (6%) [THC2 | -3,19747 |
| TNFRSF11B | ref|NM_002546|ens|ENST00000297350|gb|BC030155|gb|AK223155 | ref|Homo sapiens tumor necrosis factor receptor superfamily, member 11b (TNFRSF11B), mR | -3,20252 |
| SYCP2 | ref|NM_014258|gb|Y08982|ens|ENST00000357552|ens|ENST00000371001 | ref|Homo sapiens synaptonemal complex protein 2 (SYCP2), mRNA [NM_014258] | -3,2258 |
| ACOX2 | ref|NM_003500|ens|ENST00000302819|gb|X95190|gb|AK298402 | ref|Homo sapiens acyl-Coenzyme A oxidase 2, branched chain (ACOX2), mRNA [NM_003500] | -3,22658 |
| KNG1 | ref|NM_000893|ref|NM_001102416|ens|ENST00000287611|ens|ENST00000265023 | ref|Homo sapiens kininogen 1 (KNG1), transcript variant 2, mRNA [NM_000893] | -3,22854 |
| ACTN2 | ref|NM_001103|ens|ENST00000366578|gb|BC047901|gb|CR593118 | ref|Homo sapiens actinin, alpha 2 (ACTN2), mRNA [NM_001103] | -3,23128 |
| ITGAL | ref|NM_002209|ref|NM_001114380|gb|BC008777|ens|ENST00000315171 | ref|Homo sapiens integrin, alpha L (antigen CD11A (p180), lymphocyte function-associate | -3,23354 |
| AF113674 | gb|AF113674|thc|THC2485549 | gb|Homo sapiens clone FLB1727 PRO0398 mRNA, complete cds. [AF113674] | -3,26214 |
| FLJ34651 | gb|AK091970|ens|ENST00000326341|gb|AX747284|gb|DB515233 | gb|Homo sapiens cDNA FLJ34651 fis, clone KIDNE2018167. [AK091970] | -3,28016 |
| GSTO2 | ref|NM_183239|gb|BC056918|ens|ENST00000338595|ens|ENST00000369708 | ref|Homo sapiens glutathione S-transferase omega 2 (GSTO2), mRNA [NM_183239] | -3,29378 |
| APOM | ref|NM_019101|gb|BC020683|ens|ENST00000375916|ens|ENST00000375920 | ref|Homo sapiens apolipoprotein M (APOM), mRNA [NM_019101] | -3,30603 |
| SERPIND1 | ref|NM_000185|ens|ENST00000215727|gb|AK225292|gb|E00997 | ref|Homo sapiens serpin peptidase inhibitor, clade D (heparin cofactor), member 1 (SERP | -3,30769 |
| CPB2 | ref|NM_001872|ref|NM_016413|ens|ENST00000181383|gb|CR626525 | ref|Homo sapiens carboxypeptidase B2 (plasma) (CPB2), transcript variant 1, mRNA [NM_00 | -3,32094 |
| FKBP10 | ref|NM_021939|gb|AK092708|gb|AK025874|ens|ENST00000357255 | ref|Homo sapiens FK506 binding protein 10, 65 kDa (FKBP10), mRNA [NM_021939] | -3,32482 |
| C1orf105 | ref|NM_139240|gb|AL035295|ens|ENST00000367727|ens|ENST00000392074 | ref|Homo sapiens chromosome 1 open reading frame 105 (C1orf105), mRNA [NM_139240] | -3,33119 |
| SERPINA10 | ref|NM_016186|ref|NM_001100607|ens|ENST00000261994|ens|ENST00000393096 | ref|Homo sapiens serpin peptidase inhibitor, clade A (alpha-1 antiproteinase, antitryps | -3,33131 |
| LOC100144602 | gb|BC017721|thc|THC2694818 | gb|Homo sapiens hypothetical, mRNA (cDNA clone IMAGE:4429392), partial cds. [BC017721] | -3,3558 |
| TNFSF4 | ref|NM_003326|gb|BC041663|ens|ENST00000367718|ens|ENST00000281834 | ref|Homo sapiens tumor necrosis factor (ligand) superfamily, member 4 (TNFSF4), mRNA [N | -3,36871 |
| MST1 | gb|BC044862|gb|AK125231|gb|CR589990|gb|AK307907 | gb|Homo sapiens macrophage stimulating 1 (hepatocyte growth factor-like), mRNA (cDNA cl | -3,37016 |
| SP5 | ref|NM_001003845|ens|ENST00000375281|gb|AB096175|gb|BC069026 | ref|Homo sapiens Sp5 transcription factor (SP5), mRNA [NM_001003845] | -3,38691 |
| LOC643143 | ref|XM_931358|ref|XM_001714953|ref|XM_943181|thc|THC2691148 | ref|PREDICTED: Homo sapiens hypothetical LOC643143 (LOC643143), mRNA [XM_931358] | -3,38839 |
| TF | ref|NM_001063|gb|AK126941|ens|ENST00000264998|ens|ENST00000402696 | ref|Homo sapiens transferrin (TF), mRNA [NM_001063] | -3,39327 |
| HPR | ref|NM_020995|gb|X89214|ens|ENST00000228226|ens|ENST00000398131 | ref|Homo sapiens haptoglobin-related protein (HPR), mRNA [NM_020995] | -3,39553 |
| ADAMTSL4 | ref|NM_019032|gb|BC027478|gb|AF370411|ens|ENST00000271643 | ref|Homo sapiens ADAMTS-like 4 (ADAMTSL4), transcript variant 1, mRNA [NM_019032] | -3,40727 |
| THC2730628 | thc|THC2730628 | thc|Q9X6G7_STRPY (Q9X6G7) Serum opacity factor precursor (Fragment), partial (6%) [THC2 | -3,41281 |
| ADAM18 | ref|NM_014237|gb|AY358321|ens|ENST00000265707|ens|ENST00000379866 | ref|Homo sapiens ADAM metallopeptidase domain 18 (ADAM18), mRNA [NM_014237] | -3,41431 |
| SOX2OT | ref|NR_004053|gb|BC041898|gb|AK022826|gb|BC016393 | ref|Homo sapiens SOX2 overlapping transcript (non-protein coding) (SOX2OT), non-coding | -3,46724 |
| CCDC64B | gb|AL833749|gb|BC015446|gb|AL833717|thc|THC2606892 | gb|Homo sapiens mRNA; cDNA DKFZp666L166 (from clone DKFZp666L166). [AL833749] | -3,47735 |
| SGK1 | ref|NM_005627|ref|NM_001143676|ref|NM_001143678|ref|NM_001143677 | ref|Homo sapiens serum/glucocorticoid regulated kinase 1 (SGK1), transcript variant 1, | -3,4886 |
| TBX1 | ref|NM_080647|ens|ENST00000332710|gb|AF373867|thc|THC2477322 | ref|Homo sapiens T-box 1 (TBX1), transcript variant C, mRNA [NM_080647] | -3,49421 |
| CFHR1 | ref|NM_002113|ens|ENST00000367424|ens|ENST00000320493|gb|DQ892762 | ref|Homo sapiens complement factor H-related 1 (CFHR1), mRNA [NM_002113] | -3,52658 |
| ACSL3 | ref|NM_004457|ref|NM_203372|ens|ENST00000357430|ens|ENST00000392066 | ref|Homo sapiens acyl-CoA synthetase long-chain family member 3 (ACSL3), transcript var | -3,55402 |
| MAT1A | ref|NM_000429|ens|ENST00000372213|ens|ENST00000372206|gb|CR600407 | ref|Homo sapiens methionine adenosyltransferase I, alpha (MAT1A), mRNA [NM_000429] | -3,55885 |
| C4BPB | ref|NM_000716|ref|NM_001017364|ref|NM_001017365|ref|NM_001017367 | ref|Homo sapiens complement component 4 binding protein, beta (C4BPB), transcript varia | -3,56496 |
| DLX1 | ref|NM_178120|ref|NM_001038493|gb|BC036189|ens|ENST00000392581 | ref|Homo sapiens distal-less homeobox 1 (DLX1), transcript variant 1, mRNA [NM_178120] | -3,60833 |
| FAM19A4 | ref|NM_182522|ref|NM_001005527|ens|ENST00000295569|gb|AK057890 | ref|Homo sapiens family with sequence similarity 19 (chemokine (C-C motif)-like), membe | -3,61215 |
| AW389914 | gb|AW389914|gb|AW389924 | gb|AW389914 RC4-ST0173-191099-032-f06 ST0173 Homo sapiens cDNA, mRNA sequence [AW389914 | -3,63012 |
| LOC55908 | ref|NM_018687|gb|AY358356|ens|ENST00000252453|ens|ENST00000397785 | ref|Homo sapiens hepatocellular carcinoma-associated gene TD26 (LOC55908), mRNA [NM_018 | -3,63245 |
| THC2783023 | thc|THC2783023|thc|THC2467888 | thc|Q8IUM9_HUMAN (Q8IUM9) ACSL3 protein, complete [THC2467888] | -3,66777 |
| COL5A2 | ref|NM_000393|ens|ENST00000374866|gb|AB209045|gb|BC015705 | ref|Homo sapiens collagen, type V, alpha 2 (COL5A2), mRNA [NM_000393] | -3,67156 |
| CPVL | ref|NM_019029|ref|NM_031311|ens|ENST00000409850|ens|ENST00000265394 | ref|Homo sapiens carboxypeptidase, vitellogenic-like (CPVL), transcript variant 2, mRNA | -3,69441 |
| THC2624002 | thc|THC2624002|thc|THC2730601|gb|BM931568 | thc|Q9BXR7_HUMAN (Q9BXR7) Interleukin 10 (Fragment), partial (93%) [THC2730601] | -3,72652 |
| BM980974 | gb|BM980974 | gb|BM980974 UI-CF-EN1-ade-p-19-0-UI.s1 UI-CF-EN1 Homo sapiens cDNA clone UI-CF-EN1-ade- | -3,73383 |
| MCTP1 | ref|NM_024717|ref|NM_001002796|gb|BC030005|gb|AY656715 | ref|Homo sapiens multiple C2 domains, transmembrane 1 (MCTP1), transcript variant L, mR | -3,74205 |
| CGA | ref|NM_000735|ens|ENST00000369582|gb|V00518|gb|CR607107 | ref|Homo sapiens glycoprotein hormones, alpha polypeptide (CGA), mRNA [NM_000735] | -3,76003 |
| APOM | ref|NM_019101|gb|BC020683|ens|ENST00000375916|ens|ENST00000375920 | ref|Homo sapiens apolipoprotein M (APOM), mRNA [NM_019101] | -3,76233 |
| C8G | ref|NM_000606|ens|ENST00000224181|gb|M17263|gb|BC113624 | ref|Homo sapiens complement component 8, gamma polypeptide (C8G), mRNA [NM_000606] | -3,78542 |
| ACSL3 | ref|NM_004457|ref|NM_203372|gb|BC041692|ens|ENST00000357430 | ref|Homo sapiens acyl-CoA synthetase long-chain family member 3 (ACSL3), transcript var | -3,80459 |
| THC2603732 | thc|THC2603732 | Unknown | -3,82412 |
| ADCY10 | ref|NM_018417|gb|AF331033|gb|AL035122|gb|AF299350 | ref|Homo sapiens adenylate cyclase 10 (soluble) (ADCY10), mRNA [NM_018417] | -3,84077 |
| LOC283859 | gb|AK000276|thc|THC2605137 | gb|Homo sapiens cDNA FLJ20269 fis, clone HEP01293. [AK000276] | -3,86573 |
| GNG4 | ref|NM_004485|ref|NM_001098722|ref|NM_001098721|ens|ENST00000366597 | ref|Homo sapiens guanine nucleotide binding protein (G protein), gamma 4 (GNG4), transc | -3,92569 |
| GALC | ref|NM_000153|gb|BC036518|ens|ENST00000393568|ens|ENST00000393569 | ref|Homo sapiens galactosylceramidase (GALC), transcript variant 1, mRNA [NM_000153] | -3,98251 |
| RHOH | ref|NM_004310|ens|ENST00000381799|gb|BC014261|gb|CR619539 | ref|Homo sapiens ras homolog gene family, member H (RHOH), mRNA [NM_004310] | -4,00702 |
| KCNJ5 | ref|NM_000890|ens|ENST00000338350|gb|U52154|thc|THC2484418 | ref|Homo sapiens potassium inwardly-rectifying channel, subfamily J, member 5 (KCNJ5), | -4,01589 |
| KLHL34 | ref|NM_153270|ens|ENST00000379499|gb|AK092279|gb|AX747450 | ref|Homo sapiens kelch-like 34 (Drosophila) (KLHL34), mRNA [NM_153270] | -4,03817 |
| HP | ref|NM_005143|ref|NM_020995|ref|NM_001126102|gb|X89214 | ref|Homo sapiens haptoglobin (HP), transcript variant 1, mRNA [NM_005143] | -4,03982 |
| ST6GAL1 | ref|NM_173216|ref|NM_173217|ref|NM_003032|ens|ENST00000169298 | ref|Homo sapiens ST6 beta-galactosamide alpha-2,6-sialyltranferase 1 (ST6GAL1), transcr | -4,08348 |
| MAN1A1 | ref|NM_005907|ens|ENST00000368466|ens|ENST00000368468|gb|X74837 | ref|Homo sapiens mannosidase, alpha, class 1A, member 1 (MAN1A1), mRNA [NM_005907] | -4,16572 |
| C2 | ref|NM_000063|gb|AK096258|ens|ENST00000375510|ens|ENST00000375488 | ref|Homo sapiens complement component 2 (C2), mRNA [NM_000063] | -4,1778 |
| BEX1 | ref|NM_018476|ens|ENST00000372728|gb|AF237783|gb|CR613496 | ref|Homo sapiens brain expressed, X-linked 1 (BEX1), mRNA [NM_018476] | -4,20972 |
| CSTA | ref|NM_005213|ens|ENST00000264474|gb|X05978|gb|BC010379 | ref|Homo sapiens cystatin A (stefin A) (CSTA), mRNA [NM_005213] | -4,27647 |
| PCOLCE2 | ref|NM_013363|ens|ENST00000295992|gb|CR601643|gb|CR608532 | ref|Homo sapiens procollagen C-endopeptidase enhancer 2 (PCOLCE2), mRNA [NM_013363] | -4,30015 |
| MAN1A1 | ref|NM_005907|ens|ENST00000368466|ens|ENST00000368468|gb|X74837 | ref|Homo sapiens mannosidase, alpha, class 1A, member 1 (MAN1A1), mRNA [NM_005907] | -4,32165 |
| CSTA | ref|NM_005213|ens|ENST00000264474|gb|CR456914|gb|X05978 | ref|Homo sapiens cystatin A (stefin A) (CSTA), mRNA [NM_005213] | -4,33001 |
| GLT1D1 | ref|NM_144669|gb|BC043528|ens|ENST00000281703|gb|CR626112 | ref|Homo sapiens glycosyltransferase 1 domain containing 1 (GLT1D1), mRNA [NM_144669] | -4,35839 |
| FAM26F | ref|NM_001010919|ens|ENST00000368606|ens|ENST00000368605|ens|ENST00000368604 | ref|Homo sapiens family with sequence similarity 26, member F (FAM26F), mRNA [NM_001010 | -4,36423 |
| JCLN | ref|NM_152338|gb|BC029149|thc|THC2471936 | ref|Homo sapiens jacalin-like lectin domain containing (JCLN), mRNA [NM_152338] | -4,36427 |
| FAM101A | ref|NM_181709|ens|ENST00000324038|ens|ENST00000389727|gb|CR602224 | ref|Homo sapiens family with sequence similarity 101, member A (FAM101A), mRNA [NM_1817 | -4,39188 |
| C2 | ref|NM_000063|gb|AK096258|ens|ENST00000375493|ens|ENST00000375510 | ref|Homo sapiens complement component 2 (C2), mRNA [NM_000063] | -4,41442 |
| ACSL1 | ref|NM_001995|ens|ENST00000281455|gb|L09229|gb|CR598751 | ref|Homo sapiens acyl-CoA synthetase long-chain family member 1 (ACSL1), mRNA [NM_00199 | -4,4166 |
| SLC6A14 | ref|NM_007231|ens|ENST00000371900|gb|AK304243|gb|AK313390 | ref|Homo sapiens solute carrier family 6 (amino acid transporter), member 14 (SLC6A14), | -4,44199 |
| BMP7 | ref|NM_001719|ens|ENST00000371291|gb|X51801|gb|BC008584 | ref|Homo sapiens bone morphogenetic protein 7 (BMP7), mRNA [NM_001719] | -4,48808 |
| THC2606976 | thc|THC2606976|gb|T26612|gb|AI265938|gb|AW664748 | thc|1102236A protein,myelin basic. {Sus scrofa domestica} (exp=-1; wgp=0; cg=0), partia | -4,54084 |
| HOXD1 | ref|NM_024501|gb|BC014477|ens|ENST00000409847|ens|ENST00000331462 | ref|Homo sapiens homeobox D1 (HOXD1), mRNA [NM_024501] | -4,71153 |
| SEMA5A | ref|NM_003966|ens|ENST00000382496|gb|AF056434|thc|THC2619642 | ref|Homo sapiens sema domain, seven thrombospondin repeats (type 1 and type 1-like), tr | -4,72134 |
| AHSG | ref|NM_001622|ens|ENST00000273784|gb|CR601258|gb|CR592598 | ref|Homo sapiens alpha-2-HS-glycoprotein (AHSG), mRNA [NM_001622] | -4,74391 |
| C10orf116 | ref|NM_006829|ens|ENST00000372012|ens|ENST00000372013|gb|D45370 | ref|Homo sapiens chromosome 10 open reading frame 116 (C10orf116), mRNA [NM_006829] | -4,76628 |
| OSTbeta | ref|NM_178859|ens|ENST00000395712|ens|ENST00000334287|gb|BC103842 | ref|Homo sapiens organic solute transporter beta (OSTbeta), mRNA [NM_178859] | -4,8095 |
| SLC16A5 | ref|NM_004695|ens|ENST00000329783|gb|CR618619|gb|AK092512 | ref|Homo sapiens solute carrier family 16, member 5 (monocarboxylic acid transporter 6) | -4,82701 |
| SAA4 | ref|NM_006512|ens|ENST00000278222|gb|CR541758|gb|M81349 | ref|Homo sapiens serum amyloid A4, constitutive (SAA4), mRNA [NM_006512] | -4,85952 |
| KCNJ5 | ref|NM_000890|ens|ENST00000338350|gb|D50134|gb|U52154 | ref|Homo sapiens potassium inwardly-rectifying channel, subfamily J, member 5 (KCNJ5), | -4,87404 |
| GALC | ref|NM_000153|gb|BC036518|ens|ENST00000393568|ens|ENST00000393569 | ref|Homo sapiens galactosylceramidase (GALC), transcript variant 1, mRNA [NM_000153] | -4,89637 |
| GPR109B | ref|NM_006018|ref|NM_177551|gb|D10923|gb|BC038955 | ref|Homo sapiens G protein-coupled receptor 109B (GPR109B), mRNA [NM_006018] | -4,91691 |
| CFH | ref|NM_000186|ref|NM_002113|ens|ENST00000367429|ens|ENST00000320493 | ref|Homo sapiens complement factor H (CFH), transcript variant 1, mRNA [NM_000186] | -4,94892 |
| LOC146429 | gb|AL137382|gb|XM_370997|ref|XM_001714568|ref|XM_946336 | gb|Homo sapiens mRNA; cDNA DKFZp434L1226 (from clone DKFZp434L1226). [AL137382] | -4,96613 |
| CFHR5 | ref|NM_030787|gb|BC111773|ens|ENST00000256785|ens|ENST00000367414 | ref|Homo sapiens complement factor H-related 5 (CFHR5), mRNA [NM_030787] | -5,02319 |
| COL5A2 | ref|NM_000393|gb|BC043613|ens|ENST00000374866|gb|Y14690 | ref|Homo sapiens collagen, type V, alpha 2 (COL5A2), mRNA [NM_000393] | -5,11701 |
| NQO1 | ref|NM_000903|ref|NM_001025433|ref|NM_001025434|ens|ENST00000320623 | ref|Homo sapiens NAD(P)H dehydrogenase, quinone 1 (NQO1), transcript variant 1, mRNA [N | -5,16558 |
| HAL | ref|NM_002108|gb|BC096096|ens|ENST00000261208|gb|BC096097 | ref|Homo sapiens histidine ammonia-lyase (HAL), mRNA [NM_002108] | -5,2629 |
| THC2635964 | thc|THC2635964 | thc|Q4WTS6_ASPFU (Q4WTS6) Peptidyl-tRNA hydrolase, partial (6%) [THC2635964] | -5,28798 |
| PLAC8 | ref|NM_016619|ref|NM_001130716|ens|ENST00000311507|gb|EU570986 | ref|Homo sapiens placenta-specific 8 (PLAC8), transcript variant 2, mRNA [NM_016619] | -5,36511 |
| LOX | ref|NM_002317|ens|ENST00000395480|ens|ENST00000231004|gb|AK311354 | ref|Homo sapiens lysyl oxidase (LOX), mRNA [NM_002317] | -5,4667 |
| TMEM42 | ref|NM_144638|ens|ENST00000383743|ens|ENST00000302392|gb|CR591568 | ref|Homo sapiens transmembrane protein 42 (TMEM42), mRNA [NM_144638] | -5,48496 |
| AGXT2L1 | ref|NM_031279|gb|AJ298293|ens|ENST00000296486|gb|BC022526 | ref|Homo sapiens alanine-glyoxylate aminotransferase 2-like 1 (AGXT2L1), mRNA [NM_03127 | -5,49747 |
| ANGPTL3 | ref|NM_014495|ens|ENST00000371129|gb|BC007059|gb|AK315304 | ref|Homo sapiens angiopoietin-like 3 (ANGPTL3), mRNA [NM_014495] | -5,5157 |
| PLAC8 | ref|NM_016619|ref|NM_001130716|ens|ENST00000311507|gb|AJ422147 | ref|Homo sapiens placenta-specific 8 (PLAC8), transcript variant 2, mRNA [NM_016619] | -5,57075 |
| SLC6A14 | ref|NM_007231|ens|ENST00000371900|gb|AF151978|gb|BQ016954 | ref|Homo sapiens solute carrier family 6 (amino acid transporter), member 14 (SLC6A14), | -5,58799 |
| TDGF1 | ref|NM_003212|ref|NR_002718|gb|M96956|ens|ENST00000296145 | ref|Homo sapiens teratocarcinoma-derived growth factor 1 (TDGF1), mRNA [NM_003212] | -5,63002 |
| CTHRC1 | ref|NM_138455|ens|ENST00000297577|ens|ENST00000330295|gb|CR592899 | ref|Homo sapiens collagen triple helix repeat containing 1 (CTHRC1), mRNA [NM_138455] | -5,6642 |
| SOX2 | ref|NM_003106|ens|ENST00000325404|gb|CR592780|thc|THC2467017 | ref|Homo sapiens SRY (sex determining region Y)-box 2 (SOX2), mRNA [NM_003106] | -5,72338 |
| SPINK1 | ref|NM_003122|ens|ENST00000296695|gb|Y00705|gb|M11949 | ref|Homo sapiens serine peptidase inhibitor, Kazal type 1 (SPINK1), mRNA [NM_003122] | -5,76824 |
| CD36 | ref|NM_001001547|ref|NM_000072|ref|NM_001127444|ref|NM_001127443 | ref|Homo sapiens CD36 molecule (thrombospondin receptor) (CD36), transcript variant 2, | -5,76917 |
| COMP | ref|NM_000095|gb|AK074508|ens|ENST00000222271|gb|AB086984 | ref|Homo sapiens cartilage oligomeric matrix protein (COMP), mRNA [NM_000095] | -5,84911 |
| SSTR2 | ref|NM_001050|ens|ENST00000357585|gb|E11322|gb|BT019926 | ref|Homo sapiens somatostatin receptor 2 (SSTR2), mRNA [NM_001050] | -5,91018 |
| SYT17 | ref|NM_016524|ens|ENST00000355377|ens|ENST00000396244|gb|AK098747 | ref|Homo sapiens synaptotagmin XVII (SYT17), mRNA [NM_016524] | -6,22731 |
| SSTR2 | ref|NM_001050|ens|ENST00000357585|gb|CR593522|gb|BC000256 | ref|Homo sapiens somatostatin receptor 2 (SSTR2), mRNA [NM_001050] | -6,52162 |
| DQX1 | ref|NM_133637|ens|ENST00000272440|ens|ENST00000404568|ens|ENST00000393951 | ref|Homo sapiens DEAQ box polypeptide 1 (RNA-dependent ATPase) (DQX1), mRNA [NM_133637] | -6,53589 |
| AHSG | ref|NM_001622|gb|AF130057|ens|ENST00000273784|gb|D67012 | ref|Homo sapiens alpha-2-HS-glycoprotein (AHSG), mRNA [NM_001622] | -6,60168 |
| THBS4 | ref|NM_003248|gb|Z19585|ens|ENST00000350881|gb|AK313587 | ref|Homo sapiens thrombospondin 4 (THBS4), mRNA [NM_003248] | -6,70738 |
| F2 | ref|NM_000506|gb|BC051332|ens|ENST00000311907|gb|AJ972449 | ref|Homo sapiens coagulation factor II (thrombin) (F2), mRNA [NM_000506] | -6,73714 |
| CLDN5 | ref|NM_003277|ref|NM_001130861|ens|ENST00000403084|ens|ENST00000406028 | ref|Homo sapiens claudin 5 (CLDN5), transcript variant 2, mRNA [NM_003277] | -7,00337 |
| BQ417071 | gb|BQ417071|thc|THC2662387|thc|THC2675378 | gb|ik41d04.y1 HR85 islet Homo sapiens cDNA clone IMAGE: 5783478 5', mRNA sequence [BQ41 | -7,15509 |
| NOSTRIN | ref|NM_052946|ref|NM_001039724|gb|AK093444|gb|BC093072 | ref|Homo sapiens nitric oxide synthase trafficker (NOSTRIN), transcript variant 1, mRNA | -7,25283 |
| AK023515 | gb|AK023515|thc|THC2604696 | gb|Homo sapiens cDNA FLJ13453 fis, clone PLACE1003205. [AK023515] | -7,28572 |
| DLK1 | ref|NM_003836|ens|ENST00000331224|ens|ENST00000341267|gb|CR608554 | ref|Homo sapiens delta-like 1 homolog (Drosophila) (DLK1), mRNA [NM_003836] | -7,35382 |
| SLC16A6 | ref|NM_004694|ens|ENST00000327268|ens|ENST00000344066|gb|AX750577 | ref|Homo sapiens solute carrier family 16, member 6 (monocarboxylic acid transporter 7) | -7,40281 |
| TDGF3 | gb|M96956|thc|THC2490284 | gb|Human (clone CR-3) teratocarcinoma-derived growth factor 3 (TDGF3) mRNA, complete cd | -7,65386 |
| ZNF257 | ref|NM_033468|gb|BC036446|gb|AK056694|gb|AF070651 | ref|Homo sapiens zinc finger protein 257 (ZNF257), mRNA [NM_033468] | -7,68306 |
| SLC16A6 | ref|NM_004694|ens|ENST00000327268|ens|ENST00000344066|gb|AX750577 | ref|Homo sapiens solute carrier family 16, member 6 (monocarboxylic acid transporter 7) | -7,68506 |
| A2M | ref|NM_000014|ens|ENST00000318602|ens|ENST00000404455|gb|AK312482 | ref|Homo sapiens alpha-2-macroglobulin (A2M), mRNA [NM_000014] | -7,7021 |
| AIM1 | ref|NM_001624|ens|ENST00000369066|ens|ENST00000285105|gb|CR600498 | ref|Homo sapiens absent in melanoma 1 (AIM1), mRNA [NM_001624] | -7,7274 |
| MUM1L1 | ref|NM_152423|ens|ENST00000337685|ens|ENST00000372552|ens|ENST00000357175 | ref|Homo sapiens melanoma associated antigen (mutated) 1-like 1 (MUM1L1), mRNA [NM_1524 | -7,91497 |
| CKM | ref|NM_001824|ens|ENST00000221476|gb|AK129878|gb|AK300730 | ref|Homo sapiens creatine kinase, muscle (CKM), mRNA [NM_001824] | -8,02011 |
| TRIM59 | ref|NM_173084|ens|ENST00000309784|thc|THC2525484 | ref|Homo sapiens tripartite motif-containing 59 (TRIM59), mRNA [NM_173084] | -8,28234 |
| C1orf64 | ref|NM_178840|gb|BC017946|thc|THC2660236|thc|THC2475960 | ref|Homo sapiens chromosome 1 open reading frame 64 (C1orf64), mRNA [NM_178840] | -8,32523 |
| SLC1A3 | ref|NM_004172|ens|ENST00000265113|ens|ENST00000381918|gb|CR601776 | ref|Homo sapiens solute carrier family 1 (glial high affinity glutamate transporter), m | -8,36572 |
| ADH4 | ref|NM_000670|ens|ENST00000265512|gb|BC022319|gb|AK295556 | ref|Homo sapiens alcohol dehydrogenase 4 (class II), pi polypeptide (ADH4), mRNA [NM_00 | -8,55656 |
| SLC1A3 | ref|NM_004172|gb|AY954110|ens|ENST00000265113|ens|ENST00000381918 | ref|Homo sapiens solute carrier family 1 (glial high affinity glutamate transporter), m | -9,00412 |
| G0S2 | ref|NM_015714|ens|ENST00000367029|gb|BC009694|thc|THC2463882 | ref|Homo sapiens G0/G1switch 2 (G0S2), mRNA [NM_015714] | -9,1195 |
| NTS | ref|NM_006183|ens|ENST00000256010|gb|AK312066|gb|CR541936 | ref|Homo sapiens neurotensin (NTS), mRNA [NM_006183] | -10,4636 |
| PDGFRB | ref|NM_002609|ens|ENST00000261799|gb|J03278|gb|M21616 | ref|Homo sapiens platelet-derived growth factor receptor, beta polypeptide (PDGFRB), mR | -10,5706 |
| PRR15 | ref|NM_175887|ens|ENST00000319694|gb|BC029131|gb|BC018144 | ref|Homo sapiens proline rich 15 (PRR15), mRNA [NM_175887] | -11,4339 |
| GPAM | ref|NM_020918|ens|ENST00000348367|gb|AB046780|gb|AK025752 | ref|Homo sapiens glycerol-3-phosphate acyltransferase, mitochondrial (GPAM), nuclear ge | -12,0615 |
| CHODL | ref|NM_024944|ens|ENST00000299295|ens|ENST00000338326|ens|ENST00000400128 | ref|Homo sapiens chondrolectin (CHODL), mRNA [NM_024944] | -13,5285 |
| CFHR2 | ref|NM_005666|ens|ENST00000367415|gb|X64877|gb|AX772812 | ref|Homo sapiens complement factor H-related 2 (CFHR2), mRNA [NM_005666] | -14,2516 |
| ARG1 | ref|NM_000045|gb|AY074488|gb|BC005321|ens|ENST00000356962 | ref|Homo sapiens arginase, liver (ARG1), mRNA [NM_000045] | -27,5678 |
