# Supplementary material for: NUPR1, a new target in liver cancer: implication in controlling cell growth, migration, invasion and sorafenib resistance
Source: Cell Death Dis. 2016 Jun 23;7(6):e2269–. doi: 10.1038/cddis.2016.175 (PMC5143401; doi:10.1038/cddis.2016.175)
Supplement: Supplementary Table S5 [file cddis2016175x5.doc]

**Supplementary Figure 1. NUPR1 regulates cell viability, growth, proliferation, migration and invasion of HCC cells.** (A) Cell viability of HCC cells transfected with siNUPR1 #2 and siNC was assessed by MTS assay after treatment with the indicated concentrations of sorafenib for 48 hours. Data are expressed as reported in Figure 4. *p < 0.05, **p < 0.01. (B) Representative images of clonogenic assay of HCC cells transfected with siNUPR1 #2 and siNC. The experiment continued for 14 days. Surviving colonies were stained and counted. Data are expressed as reported in Figure 4. (C) Cell viability of stable Hep3B cells harboring *NUPR1* shRNA (shNUPR1 #1) or pSilencer was assessed by counting the trypan blue-negative cells after treatment with the indicated concentrations of sorafenib for 48 hours. Data are expressed as s the percentage of control cells and are the means ± SD of three separate experiments, each performed in triplicate. (D) Cell proliferation (cell index) of stable Hep3B cells harboring *NUPR1* shRNA (shNUPR1 #1) or pSilencer was analyzed using the xCELLigence RTCA instrument. *p < 0.005. (E) Representative images of wound-healing assay after *NUPR1* siRNA-mediated gene silencing (shNUPR1 #2) in PLC/PRF/5. The experiment was conducted for 48 hours. Data are expressed as reported in Figure 4. *p < 0.05. (F) Matrigel invasion assay in shNUPR1 cells (shNUPR #2) compared to pSilencer as control. Data are expressed as reported in Figure 4. *p < 0.005.

**Supplementary Figure 2. NUPR1 regulates expression of *RELB* and *IER3* genes, and RELB and IER3 regulate cell viability of HCC cells.** (A) Gene expression analysis by qPCR in HCC cells after *NUPR1* gene silencing (siNUPR1 #2). Data are expressed as reported in Figure 5. (B) Gene expression analysis, by qPCR, in Hep3B shNUPR1 (shNUPR1 #2) cells compared to pSilencer, as control. Data are expressed as reported in Figure 5. (C) Western blot analysis of p-ERK1/2 (Thr202/Tyr204) and total ERK1/2 in Hep3B shNUPR1 (shNUPR #2) and in control cells (pSilencer). (D) Gene expression analyses by quantitative-PCR after *RELB* (siRELB #2) and *IER3* (siIER3 #2) gene silencing in Hep3B cells. Data are expressed as reported in Figure 5. (E) Cell viability of Hep3B cells transfected with siRELB (siRELB #2), siIER3 (siIER3 #2) and siNC was assessed by MTS assay after treatment with the indicated sorafenib concentrations for 48 hours. Data are expressed as reported in Figure 4. *p < 0.05.

**Supplementary Figure 3. RUNX2 regulates cell viability and the expression of *NUPR1,* *RELB* and *IER3* genes in Hep3B cells.** (A) Gene expression analysis by qPCR after *NUPR1* gene silencing (shNUPR1 #2) in Hep3B cells. (B) Cell viability of Hep3B cells transfected with siRUNX2 (siRUNX2 #2) and siNC was assessed by MTS assay after treatment with the indicated concentrations of sorafenib for 48 hours. Data are expressed as reported in Figure 4. (C) Gene expression analysis after *RUNX2* gene silencing (siRUNX2 #2) in Hep3B cells performed by qPCR. Data are expressed as reported in Figure 5.

**Supplemenatry Figure 4. *In silico* analysis of the NUPR1 promoter.** Human genomic sequence of the *NUPR1* promoter located between 3003 bp downstream and 2897 bp upstream ATG codon is shown. Red box: CAT codon; green boxes: consensus sequences of RUNX2.
