# Supplementary material for: NUPR1, a new target in liver cancer: implication in controlling cell growth, migration, invasion and sorafenib resistance
Source: Cell Death Dis. 2016 Jun 23;7(6):e2269–. doi: 10.1038/cddis.2016.175 (PMC5143401; doi:10.1038/cddis.2016.175)
Supplement: Supplementary Table S6 [file cddis2016175x6.doc]

**Supplementary Table 5.** Selected differentially expressed functional gene groups (≥ 3-fold; p-value cutoff of 0.05) in HCC cells upon NUPR1 knock-down.

| **Category** | **B-H p-value** | **Molecules** |
| --- | --- | --- |
| **A. Functional analysis of up-regulated genes** | | |
| Cellular Development | 3.51E-05-3.44E-02 | *LRRN1, CDH2, FOXJ1, DKK3, EFNA5, MITF, ANGPTL4, NCF2, CXCR4, ARHGAP24, MYOF, BCL2A1, EDN1, MAP1B, SPP1, BDNF, CBLN2, UGCG, HAS3, NFIB, TGFB2, DYRK3, SULT1E1, MAF, IL18, UGT2B15, CDKN2B, INPP4B, GJA1, ABCB1, CXCL12, CD59, EFEMP1, MT2A, SEMA3E, ITGA3, NDRG1, NPY1R, OXTR, AREG, HAS2, IL11, NLGN1, SCD5, VTCN1, WLS, IL1RAP, CST6, MLLT3, IRS2, UCHL1, LRRC8C, UGT2B17, S100A6, SMOC1, FEZ1, MMP7, IGFBP1, IGFBP7, CTBP2, AKR1B10, SYNE1, CD109, FHL2, TIMP1, AXL, SRPX2, PTHLH, RARB* |
| Cellular Growth and Proliferation | 3.51E-05-3.44E-02 | *LRRN1, MAGEA4, PKHD1, CDH2, FOXJ1, DKK3, MITF, ANGPTL4, PAK7, NCF2, CXCR4, ARHGAP24, MYOF, BCL2A1, EDN1, MAP1B, PRSS2, EMP2, SPEG, SPP1, BDNF, TNFRSF19, FBN1, DCBLD2, HAS3, UGCG, NFIB, NTN4, TGFB2, B3GNT3, SULT1E1, IL18, UGT2B15, FXYD2, CDKN2B, TM4SF4, INPP4B, PLK2, GJA1, EIF4E3, ABCB1, CXCL12, CD59, EFEMP1, MAGEA3/MAGEA6, MT2A, ITGA3, NDRG1, NPY1R, CLDN1, OXTR, HAS2, AREG, IL11, SCD5, NLGN1, GAREM, VTCN1, WLS, CST6, MLLT3, P3H2, IRS2, UCHL1, UGT2B17, S100A6, FEZ1, MMP7, IGFBP1, IGFBP7, CTBP2, AKR1B10, CD109, FHL2, TIMP1, AXL, PTHLH, RARB, SUCNR1* |
| Cellular Assembly and Organization | 1.52E-04-3.44E-02 | *PKHD1, CXCL12, CDH2, FOXJ1, MT2A, SEMA3E, DKK3, ITGA3, NDRG1, EFNA5, CLDN1, ANGPTL4, PAK7, NCF2, AREG, HAS2, ARHGAP24, CXCR4, NLGN1, VTCN1, TMEM17, MAP1B, IL1RAP, EDN1, EMP2, APBB1IP, SPP1, BDNF, ARHGEF18, SYTL2, UGCG, UCHL1, NFIB, TGFB2, FEZ1, TM4SF1, MMP7, IGFBP1, SYNE1, MARK1, FHL2, PLK2, AXL, EHD2, GJA1* |
| Cellular Function and Maintenance | 1.52E-04-3.48E-02 | *PKHD1, CDH2, FOXJ1, DKK3, EFNA5, ANGPTL4, PAK7, NCF2, CXCR4, ARHGAP24, BCL2A1, TMEM17, MAP1B, EDN1, EMP2, SPP1, BDNF, JAM2, UGCG, NFIB, NTN4, TGFB2, SULT1E1, IL18, MARK1, PLK2, EHD2, GJA1, ABCB1, CXCL12, CD59, MT2A, SEMA3E, ITGA3, NDRG1, NPY1R, CLDN1, AREG, HAS2, NLGN1, VTCN1, IL1RAP, APBB1IP, ARHGEF18, UCHL1, FEZ1, TM4SF1, MMP7, SYNE1, FHL2, AXL, SUCNR1, SPNS2* |
| Cell-To-Cell Signaling and Interaction | 2.51E-04-3.61E-02 | *CDH2, CXCL12, CD59, EFEMP1, FOXJ1, MAGEA3/MAGEA6, SEMA3E, DKK3, ITGA3, NDRG1, NPY1R, CLDN1, COLEC12, OXTR, HAS2, AREG, IL11, CXCR4, NLGN1, VTCN1, BCL2A1, EDN1, PRSS2, CST6, SPP1, NPFFR2, BDNF, HAS3, UCHL1, TGFB2, MAF, C6, MAGEA2/MAGEA2B, MMP7, IL18, IGFBP7, SLC1A1, CDKN2B, FHL2, TIMP1, AXL, HS3ST1, PTHLH, GJA1, SUCNR1* |
| Lipid Metabolism | 5.19E-04-3.44E-02 | *ABCB1, CD59, CXCL12, DKK3, ITGA3, NPY1R, EFNA5, CYP3A4, MITF, ANGPTL4, AREG, BAAT, EDN1, SPP1, BDNF, ACAA2, IRS2, UGCG, LARGE, UGT2B17, SLC16A2, SULT1E1, IL18, SLC4A4, IGFBP7, AKR1B10, UGT2B15, SLC1A1, CYP3A5, TIMP1, PTHLH, RARB, SPNS2* |
| Cell Death and Survival | 1.05E-03-3.28E-02 | *MAGEA4, PKHD1, CDH2, DKK3, NEK11, EFNA5, CYP3A4, MITF, ANGPTL4, PAK7, RASSF8, NCF2, CXCR4, BCL2A1, MAP1B, EDN1, EMP2, SPP1, BDNF, SGCB, TNFRSF19, FBN1, MT1X, UGCG, HAS3, NTN4, NFIB, TGFB2, DYRK3, MAF, C6, MAGEA2/MAGEA2B, IL18, SLC22A3, SLC1A1, PLK2, GULP1, GJA1, ABCB1, CD59, CXCL12, EFEMP1, MT2A, MAGEA3/MAGEA6, ITGA3, NDRG1, NPY1R, AREG, HAS2, CDH6, IL11, VTCN1, BAAT, MLLT3, ST3GAL1, IRS2, UCHL1, S100A6, MMP7, IGFBP1, IGFBP7, CTBP2, SYNE1, FHL2, TIMP1, AXL, PTHLH, RARB* |
| Cell Morphology | 1.13E-03-3.44E-02 | *PKHD1, CDH2, FOXJ1, DKK3, EFNA5, MITF, ANGPTL4, PAK7, NCF2, CXCR4, ARHGAP24, TMEM17, EDN1, MAP1B, SPP1, BDNF, SGCB, DCBLD2, JAM2, UGCG, HAS3, NFIB, NTN4, TGFB2, SULT1E1, IL18, CDKN2B, INPP4B, CSTF2T, GJA1, ABCB1, CXCL12, CD59, MT2A, SEMA3E, ITGA3, NPY1R, AREG, HAS2, IL11, NLGN1, VTCN1, IL1RAP, SYTL2, IRS2, UCHL1, FEZ1, TM4SF1, IGFBP1, IGFBP7, SLC4A4, CTBP2, SYNE1, AXL, SRPX2, PTHLH, RARB* |
| Drug Metabolism | 2.7E-03-3.44E-02 | *ABCB1, IRS2, HAS3, UGCG, DKK3, CYP3A4, SULT1E1, HAS2, UGT2B15, CYP3A5, EDN1, TIMP1, SPP1* |
| Molecular Transport | 2.7E-03-3.44E-02 | *ABCB1, CXCL12, CD59, DKK3, ITGA3, NPY1R, EFNA5, CYP3A4, ANGPTL4, NCF2, CXCR4, EDN1, SPP1, BDNF, IRS2, UGCG, SLC16A2, SULT1E1, IL18, SLC4A4, SLC1A1, CYP3A5, TIMP1, TMEM27, PTHLH, SPNS2* |
| Cell Signaling | 3.97E-03-2.93E-02 | *CXCL12, CXCR4, ITGA3, EDN1, NPY1R, ANGPTL4, BDNF, PTHLH, NCF2* |
|  |  |  |
| **B. Functional analysis of down-regulated genes** | | |
| Cellular movement | 9.75E-05-3.64E-02 | *LOX, ITGAL, FGF19, NQO1, CFH, ADCY10, A2M, TDGF1, GATA3, C2, HP, KNG1, ST6GAL1, ARG1, SLC1A3, DLX1, SOX2, F2, BMP7, MST1, SEMA5A, PDGFRB, COMP, CGA, CPB2, AFP, ANGPTL3, THBS4, TNFRSF11B, TBX1, CD36, TNFSF4, NTS, SGK1, MAT1A, SSTR2, AHSG, SERPIND1* |
| Lipid Metabolism | 1.23E-04-3.64E-02 | *MBL2, ITGAL, DLK1, FGF19, ADH4, NQO1, APOM, CFH, ADCY10, A2M, GALC, HP, ACSL1, KNG1, ACOX2, CYP39A1, SLC1A3, F2, BMP7, UGT2B4, PDGFRB, CGA, AFP, G0S2, ANGPTL3, CD36, TNFSF4, SGK1, NTS, GPAM, ACSL3, KCNJ5, SSTR2, AHSG* |
| Molecular Transport | 1.23E-04-3.64E-02 | *MBL2, ITGAL, TF, DLK1, FGF19, NQO1, CFH, ADCY10, APOM, FGG, A2M, GALC, HP, ACSL1, KNG1, GNG4, CKM, ACTN2, SLC16A6, ARG1, SLC1A3, BMP7, F2, SLC16A5, ZG16, KCNS3, PDGFRB, CGA, CPB2, CTHRC1, HCAR3, AFP, ANGPTL3, SLC25A27, TNFRSF11B, CD36, TNFSF4, SGK1, NTS, GPAM, ACSL3, SLC6A14, KCNJ5, SSTR2, AHSG* |
| Small Molecule Biochemistry | 1.23E-04-3.64E-02 | *MBL2, LOX, ITGAL, DLK1, FGF19, ADH4, NQO1, CFH, ADCY10, APOM, A2M, GALC, HP, ACSL1, KNG1, CKM, ACOX2, CYP39A1, ARG1, SLC1A3, BMP7, F2, UGT2B4, PDGFRB, CGA, CPB2, HCAR3, AFP, G0S2, ANGPTL3, SLC25A27, TNFRSF11B, HAL, CD36, TNFSF4, SGK1, NTS, GPAM, ACSL3, SLC6A14, KCNJ5, SSTR2, MAT1A, AHSG* |
| Cell Signaling | 3.99E-03-3.64E-02 | *ITGAL, PDGFRB, CGA, HCAR3, CFH, ADCY10, A2M, TNFRSF11B, CD36, NTS, SGK1, KNG1, GNG4, CKM, ST6GAL1, KCNJ5, SSTR2, BMP7, F2, MST1* |
| Nucleic Acid Metabolism | 3.99E-03-3.64E-02 | *CGA, HCAR3, G0S2, NQO1, SLC25A27, CFH, ADCY10, CD36, NTS, KNG1, GPAM, CKM, SSTR2, BMP7, F2* |
| Cell-To-Cell Signaling and Interaction | 3.99E-03-3.64E-02 | *MBL2, LOX, RHOH, ITGAL, TF, FGF19, CFH, FGG, GATA3, A2M, KNG1, ST6GAL1, SLC1A3, DLX1, F2, BMP7, MST1, PDGFRB, COMP, CPB2, AFP, ANGPTL3, THBS4, TBX1, TNFRSF11B, CD36, TNFSF4, NTS, SGK1, SERPIND1* |
| Cellular Function and Maintenance | 3.99E-03-3.64E-02 | *PDGFRB, ITGAL, SAA4, FGF19, SLC25A27, TNFRSF11B, GATA3, A2M, TNFSF4, CD36, SGK1, NTS, KNG1, KCNJ5, SLC1A3, SOX2, F2, BMP7* |
| Carbohydrate Metabolism | 4.85E-03-3.64E-02 | *PDGFRB, GSTO2, ITGAL, CTHRC1, FGF19, DLK1, NQO1, ANGPTL3, SLC25A27, ADCY10, CFH, CD36, ACSL1, NTS, KNG1, GPAM, CKM, ST6GAL1, SLC1A3, SSTR2, AHSG, F2, BMP7* |
| Cell Death and Survival | 5.62E-03-3.64E-02 | *MBL2, RHOH, ITGAL, TF, FGF19, DLK1, NQO1, CFH, ADCY10, TDGF1, GATA3, A2M, HP, KNG1, ST6GAL1, CSTA, ARG1, SLC1A3, SYCP2, DLX1, ADAMTSL4, SOX2, F2, BMP7, MST1, PDGFRB, COMP, CGA, CPB2, CFHR1, G0S2, RNF144B, AFP, C8G, PLAC8, SLC25A27, THBS4, TNFRSF11B, CD36, SGK1, NTS, GPAM, SSTR2* |
| Energy Production | 6.26E-03-3.64E-02 | *CD36, ACSL1, NTS, GPAM, ACSL3, FGF19, ACOX2, ADH4, G0S2, F2* |
| Cellular Growth and Proliferation | 6.88E-03-3.64E-02 | *MBL2, LOX, RHOH, ITGAL, TF, FGF19, DLK1, NQO1, ADCY10, GATA3, TDGF1, A2M, KNG1, GNG4, ST6GAL1, ARG1, SLC1A3, SOX2, F2, BMP7, MST1, SEMA5A, PDGFRB, COMP, CGA, CTHRC1, CPB2, G0S2, RNF144B, AFP, PRG4, PLAC8, SLC25A27, THBS4, TNFRSF11B, TBX1, CD36, TNFSF4, SGK1, NTS, GPAM, SPINK1, KCNJ5, SSTR2, AHSG, SERPIND1* |
