# Supplementary material for: NUPR1, a new target in liver cancer: implication in controlling cell growth, migration, invasion and sorafenib resistance
Source: Cell Death Dis. 2016 Jun 23;7(6):e2269–. doi: 10.1038/cddis.2016.175 (PMC5143401; doi:10.1038/cddis.2016.175)
Supplement: Supplementary Table S7 [file cddis2016175x7.doc]

**Supplementary Table 6**. Fold expression of validated genes following *NUPR1* knockdown in Hep3B cells.

| **Gene** | ***microarray*** | ***sqPCR*** | ***qPCR*** |
| --- | --- | --- | --- |
| **FGF19** | -3.07 |  | -14.3 |
| **AFP** | -3.15 | -1.4 |  |
| **MT2A** | 4.59 | 1.4 |  |
| **TIMP** | 4.51 | 3.6 |  |
| **TGFB2** | 5.18 |  | 8.51 |
| **BMP7** | -4.44 |  | -52.7 |
| **PDGFRB** | -10.57 |  | -50.2 |
