# Supplementary material for: NUPR1, a new target in liver cancer: implication in controlling cell growth, migration, invasion and sorafenib resistance
Source: Cell Death Dis. 2016 Jun 23;7(6):e2269–. doi: 10.1038/cddis.2016.175 (PMC5143401; doi:10.1038/cddis.2016.175)
Supplement: Supplementary Table S8 [file cddis2016175x8.doc]

**Supplementary Table 7.** Putative binding sites of RUNX2 predicted in NUPR1 promoter sequence according to JASPAR database tool (<http://jaspar.genereg.net/>).

| **predicted site sequence** | **Score** | **Relative score** | **relative position to start codon** | **Strand** | **position (GRCh38/hg38)** |
| --- | --- | --- | --- | --- | --- |
| TCACCGCAG | 8.968 | 0.891 | +293/+301 | -1 | chr16:28,538,607-28,538,615 |
| AGACCACAG | 9.269 | 0.897 | -118/-126 | 1 | chr16:28,539,022-28,539,030 |
| TGACCTCAA | 6.872 | 0.853 | -759/-767 | 1 | chr16:28,539,663-28,539,671 |
| TCACCACAA | 9.549 | 0.902 | -1453/-1461 | -1 | chr16:28,540,357-28,540,365 |
| CTACCGCAC | 6.990 | 0.855 | -1752/1760 | -1 | chr16:28,540,656-28,540,664 |
| TGACCTCAA | 6.872 | 0.853 | -2443/-2451 | -1 | chr16:28,541,347-28,541,355 |
| TCACCGCAG | 8.968 | 0.891 | -2463/-2471 | -1 | chr16:28,541,367-28,541,375 |
| TCACCGCAA | 10.838 | 0.926 | -2738/-2746 | -1 | chr16:28,541,642-28,541,650 |
