# Supplementary material for: NUPR1, a new target in liver cancer: implication in controlling cell growth, migration, invasion and sorafenib resistance
Source: Cell Death Dis. 2016 Jun 23;7(6):e2269–. doi: 10.1038/cddis.2016.175 (PMC5143401; doi:10.1038/cddis.2016.175)
Supplement: Supplementary Table S9 [file cddis2016175x9.doc]

**Supplementary Table 8A.** Scores representing immunoreactivity for RUNX2

|  | NL  (n=3) | LC  (n=3) | HCC  (n=21) |
| --- | --- | --- | --- |
| intensity of staining | 0  (0-1) | 1.00  (1) | 1^a^  (1-2) |
| % of positive nuclei | 0  (0-1) | 1.00  (1) | 3^b,c^  (1-4) |
| sum of the scores | 0  (0-2) | 2.00  (2) | 4^d,e^  (2-6) |

Data are expressed as median (min-max) of the scores. a, *p* < 0.05 compared with scores of NL; b, *p* < 0.001 compared with scores of NL; c, *p* < 0.05 compared with scores of LC; d, *p* < 0.001 compared with scores of NL; e, *p* < 0.05 compared with scores of LC

**Supplementary Table 8B.** Correlation of RUNX2 expression with TNM and Grade

|  | TNM  n=21 | *p* | Grade  n=21 | *p* |
| --- | --- | --- | --- | --- |
| intensity | 0 | ns | 0.41 | ns |
| % postive nuclei | 0 | ns | 0.61 | 0.003 |
| sum | -0.20 | ns | 0.50 | 0.03 |
