# Supplementary figures and images for: NUPR1, a new target in liver cancer: implication in controlling cell growth, migration, invasion and sorafenib resistance
Source: Cell Death Dis. 2016 Jun 23;7(6):e2269–. doi: 10.1038/cddis.2016.175 (PMC5143401; doi:10.1038/cddis.2016.175)

Supplementary Figure 1

**A**

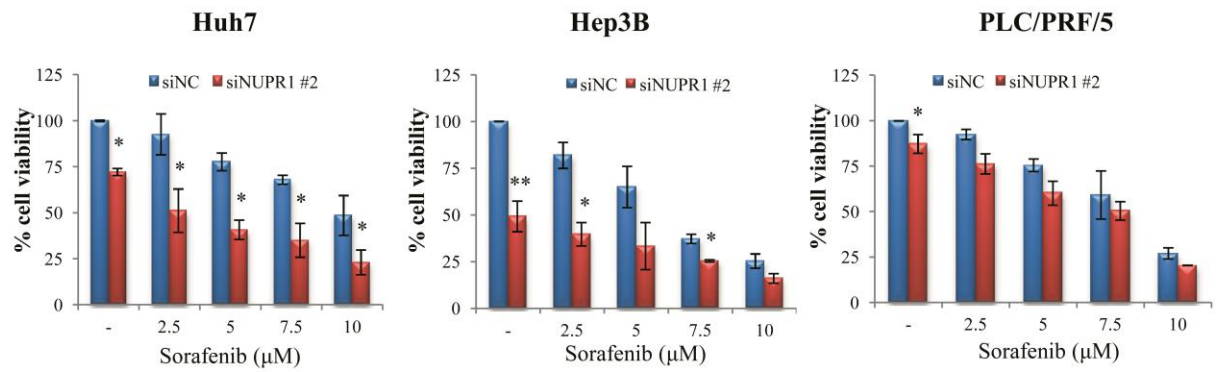

**B**

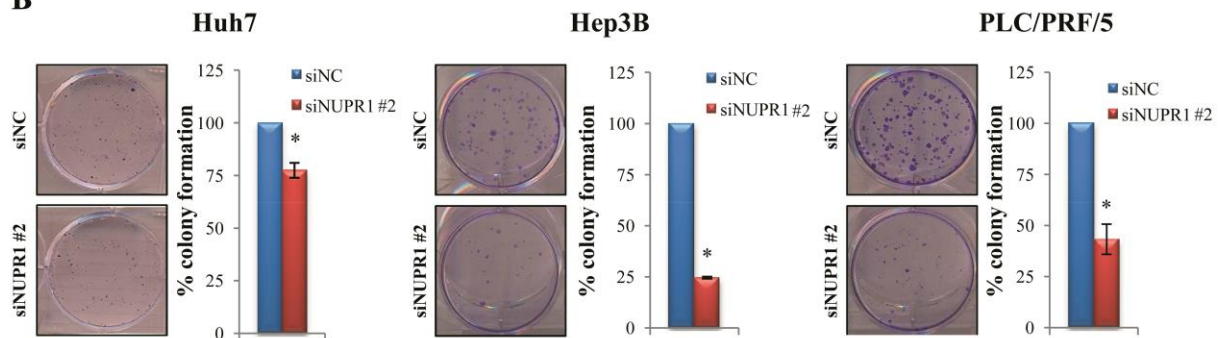

**C**

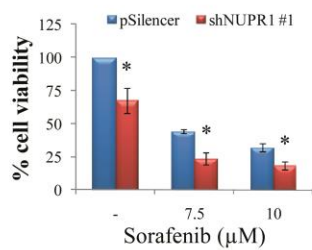

**D**

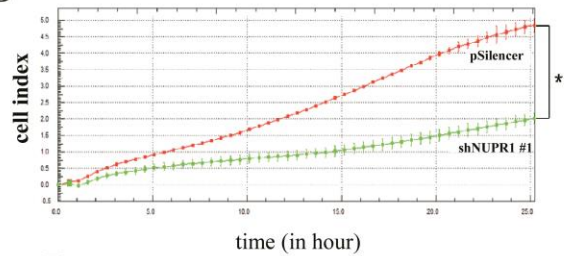

**E**

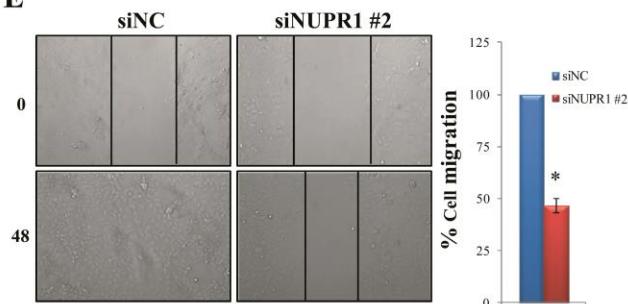

**F**

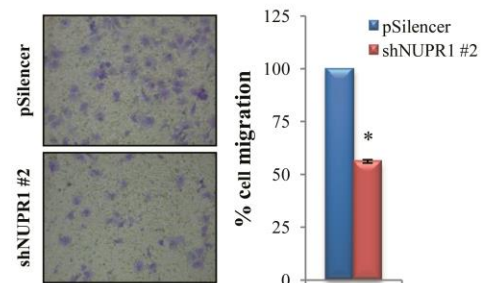

Supplement: Supplementary Figure S1 [file cddis2016175x10.pdf]

**Supplementary Figure 2**

**A**

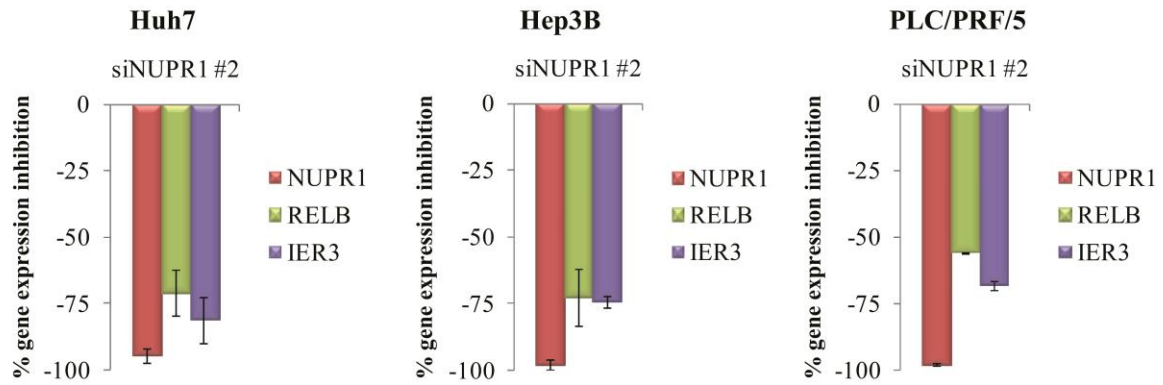

**B**

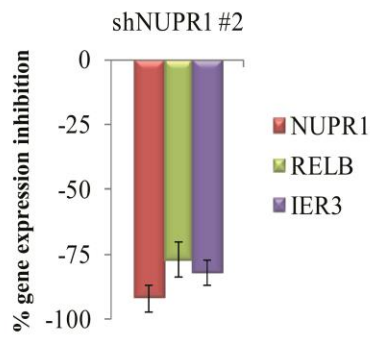

**C**

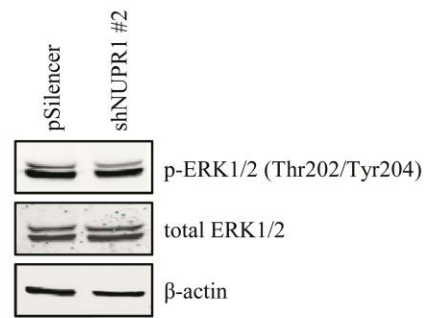

**D**

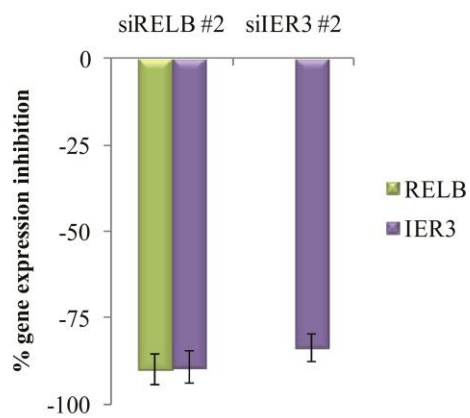

**E**

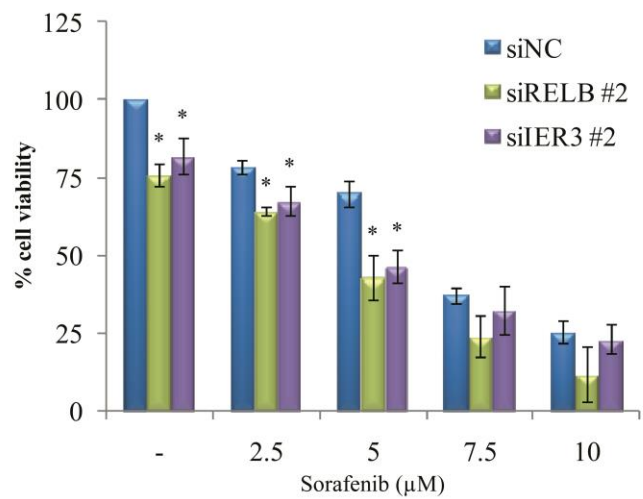

Supplement: Supplementary Figure S2 [file cddis2016175x11.pdf]

Supplementary Figure 3

A

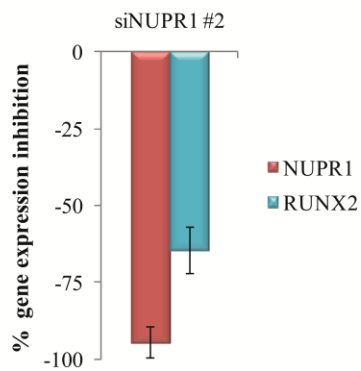

B

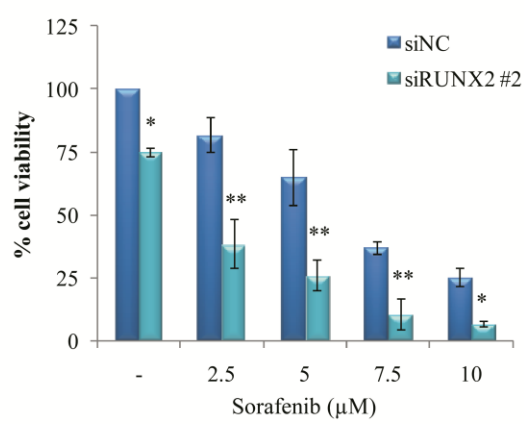

C

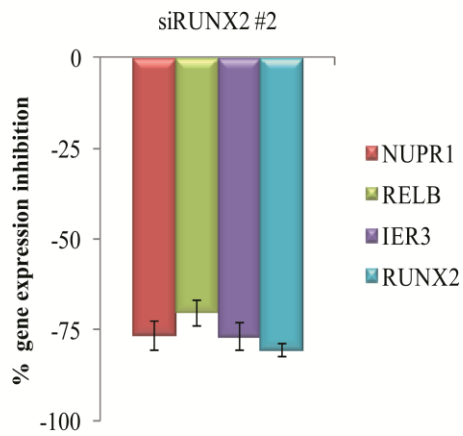

Supplement: Supplementary Figure S3 [file cddis2016175x12.pdf]
