# Supplementary material for: NUPR1, a new target in liver cancer: implication in controlling cell growth, migration, invasion and sorafenib resistance
Source: Cell Death Dis. 2016 Jun 23;7(6):e2269–. doi: 10.1038/cddis.2016.175 (PMC5143401; doi:10.1038/cddis.2016.175)
Supplement: Supplementary Figure S4 [file cddis2016175x13.pdf]

Supplementary Figure 4

CTTGGTATGTTGCCTAGGCTGGTCTCGAACTCCTGGCCTCAAGCGATCCTCCCTCCTTGGCCTCCCAAAGGCTGGAAGGCTGAGGTGG
GCAGATCATTTGAGGCCAGGAGTTTGAGACCAGCCTGGCCAACATGATGAAACCCCATCTCTACTAAAAATACAAAAATTGGCCAGG
CGTGGTGGCTGACGCCTGTAATCCCAGCACTTTGGGAGGCCAAGGTGGGTGGATCATGAGGTCAAGAGATTGAGACCATCCTGGCCA
ACATGGTGAAACTCTGTTTCTACTAAAAATACAAAAATTAGCCAGGCATGGTGGCAGGTGCCTGTAGTCCCAGCTACTCAGGAGGCTG
AAGCAGGAGAATAGCTTGAACCCAGGAGGCGGAGGTTGCAGTGAGCTGCGATCATGCCACTGCACTCCAGCCTGAGCAACAGAGTGA
GACTCTGTATCAAAAAAACAAAAAAACAAAAATTAGCCAGGCGTGGTGGTGCTCACCTGTAATCCCAGCTACTCAGGGGGCGTAGGC
ACGAGAATTGCTTGAACCCGGGAGGCAGAGGTTGCAGTGAGCCAAGATGGTGCCACTGCACTCTAACCTGAGCAACAGAGTGAGACT
CTGTCTCAAAAAAAAAAATTGTAGAGATGGGGTCTTACTATGATGCTCAGGCTGGTCTCAAACTCCTAGGCTCAAGTAATCCTCCCACT
TCGGCCTCCTAAACTGCTGTGATTACAGGCATGAGCCACTGCTCCCAGACTTTAGTTTTTGTTTGTTTGTTTGTTTTGAGACAGAGTCTC
CCTCTGTCACCCAGGCTGGAGTGCAATGGCACGATGTCGGCTCACTGCAACCTCTACCTCCTGGGTTCAAGCGATTCTACTGCCTCAGC
CTCCTGAGCAGCTGGGATTACAGGCACCTGCCACCATGCTTGGCTAATTTTTGGTATTTTTAGTGGAGATGGGGTTTCACCATGTTGGT
CAGGCTGGTCTTGAACTCCTGACTTTGTGATCCACCCACCTCGGCCTCCCAAAGTGCTGGGATTACAGGCGTGAGCCACTGCCCCCGG
CCCTGGCTTTTAATATTTTAAATCTCAGTTTTCTCTGACTTTTAGATGGCACTCATAAGTAGCGTTCCCGTCTATGAACAGGACACAAT
CAACAAAAACAGGTGCTGGGGAGGGCCGTGTTTCATGGGCAGTATCGCAAAAAAGCAGAAGAAATTCCTTTCCACTACGTAGGAGCC
CACAGAAATGTATGCACTAAAATATATGCAAGCAAAAGCAAACAAACCAAAAGCTGCATTCACAGCGTAGACCCTGCAAGAGCAGG
AACACTCCCTGCCCCAGATACATACACCAAACACAAATACACATCAAAGATGCTCTCTCAGGCTCATGAATGTGCAGGCACAGGCAA
GCACACACATGCCCGAACAAAAACACACAAAGAACAAGGATGAACACACACCCAAGCTGTGGCTGCCGTGCGTGTCTATTTATTGTT
GCTGCCACCCTGGAGGAGCCCCAGTTTCTTCTGTAGCTTTCTTTTCTGGGGGATCTTCCTGGCTCTGCCCCTCCATTCCCAGCCTCTCAC
TCCCCATCTTGCACTTTTGCTAGGGTTGGAGGCGCTTTCCTGGTAGCCCCTCAGAGACTCAGTCAGCGGGAATAAGTCCTAGGGGTGG
GGGGTGTGGCAAGCCGGCCTGGATCCTGTCCTGGGTCCTCCTTCCTCCGCAGTCCCGTCTCTATTGCTGGGTGTAGTGTCCATGGTCTG
GCCTCATCTGGGGGGTGAGGAAAAGCAGGTGGTAAAAGGGACAGAGATCTGGGTTCTAATTCTGCCTCTCCCACTCACTTGATTTGTG
ACTCCAGGGAAGGACCCACCCTCTCTATCCTCAATTTCTGTCTCTGTAAGTCAAATTTCCCTTCAATTGGAAAGGTTCTGTGTTCCCAC
TTTGCTCTGGCCACTAGGAAACTCACAATCAAACATATGTCTCGTTTTATTCTGCATCAAGACAACAGTTGCCTGATCCCTCCTCCCCA
CCCAATCACACACCTTCCGGCCTGTCCTTCATGGCAGAAGCACCACCTTGAGCTCTCTGGGCCCCCCGACTCCTTACCTCCAGCTCTGT
CTCAGCGCCGTGCCCCTCGCTTCTTCCTCTCTGAATTCTGCAGCTTGGTCACCAGTTTCCTCTCGTGCCCGCCAGGGCTGGGGCGGTTG
GTGTTGGCAGCAGCTTCTCTCTTGGTGCGACCTTTCCGGCCTCCACCTCCTGTAACCAAGGCAGGAGTCAGAGGTGAAGTGGGCATAG
GCATGATGAGAGGCCCTGTCAGAGGAGAGGCAGGGGTTCAGGGTTGTGGGGATGGGAAGAGCAGGGAGAAGCCTGCTGCTTCCCCTC
TGTGGAATGTGGGACCCCAGATGTGTGTGAGGTCCCAGGCTGAGTAACATAGCACTCCTGGGATAAGGGATTAGACAGGGGTGACGT
GAGGGTCCAAGGAACAGGTTGGCTAGAAAAGGGTGAGTGGGCTGGGCAGGGTGGCTCACTCCTGTAATCTCAGCACTTTGGGAGGCC
AAGGCAGGAGAATTGCTTGAGCCCAGGAATTTGGGATCAGCCTGGGAAACATAGTGAGACCCCATCTCACCACTGCAGGTGGTGTAC
ACCTGAAGTTCCAGCCACTCAGGAGGCTGAAGCAGGAGGATCACTTGAGCCCAGGAGTTCGAGGCTGCGGTGAGTTGTTTGAGTTGTT
GTGATCACACTACTGCACTCCAGCCTGGGTGACAGAGTGACTCTCTCAAAAAAAAAAGAAAGAAAGAAACGAAGAGGAAAGGAAAGG
AAATGAGAGGAAACAAGAGGGGTGGGTCCTGATTTCGGGAGAGGAGGAAGGACCGTGGAGATGGCTGAGTGGGCCTTACCGAGGTA
GGAATGGGCCAGGCTATAGAGGTCAGATTCATCCAGGCTGGAGTCCTCGTCCTCCGGGCCTGGGGGCTGCTGGGGGGCGCTGGTTGCT
GGTGGGAAGGTGGCCATCGTGCCTGGCTTGTCTTCCCTGCCTCTCTTCTTCTCCTAACGCTTTGTCTGTCTCTGCTTTCCTGGCCCCGGG
CCTCTCCACTCCCTCAGCTTATAAGGCTGTGTCTCCTCCTCAGACCACAGTCTGGCTCCAGCCCATCAGGCCCTGTCTGATGCAATCTG
GCCCCTTCCCAAAATCCAGCCCAGTAAACACAGTCATGAGACGGGAAGAGTCGGTCCCTGAGTCCAGCCCAACTCACCTCCTCCCTCC
CAGGCTCACCCAGCTGGATATTTTCCATAGAGGAGGTCCCGCTTCTCGGCAGAAAGCAGGGAAGAGGCAGGATCTGGGCTAAAACAC
TCGTTGGGATCATGGCCTCACCTGATCGAGGGCTTCACCTGGGCCTGGGATGACGGCCACACAGCTAACCCCGGCACCCCAATTCTCG
CTGAGATGCAGGACGTGAGGAAGGAGGGGGAAGGGATATCAGCACACTTGTGCCAGGGACAAGTCAGTGGCTTCCACCCAGTCACA
CTCAACATCTTGCTTGTCTTCTTTTTTTTTTTTGAAATGGAGTCTCTGTCGCCCGGCTGGAGTGCAGTGGTGTGATCTTGGCTCACTGCA
ACCTGCAACCTCCTCCTCCCAGGTTCAAGCAATTCTCCTGGCTCAGCCTCCCGAATAGCTGGGATTATAGACATCTGCCACCATGCCTG
GCTAATTTTTGTATTTTTAGTAGAGATGGGGTTTCACCATGTTGGACAGGCTGATCTTGAACTCCTGACCTCAAGTCATCTGCCTGCCT
CGGCCTCCCAAAGTGCTGGGATTACAGGCATGAGCCACACCCTTTTTTTTCTTTTTTCTATGAGAAACATCTTGGAAAACCCCTTAATA
TCCTGAAACAGGGTCCCCAGTAATGTAATCTCCCTCACCTAGGAGGAGGGTGGCAGAGGGTTGGAGAGTTCAGGGTCTGGAGCCAGT
TGCCCTCAGTTCAATCCCCTGACCCACCACTTTCCGTGTGACCTTGGGCGAGTCCCTTCACCTCTCTGTGCCTTGGTTTCCAATTCTGTA
AAATAAGGATGATAATAATTGTATCTTCTTCCTAGAGTTGGGGAGAGGATCAAATGAGATAATGTTTGAAAGACACATGCTACAGTGC
CCGGTGACAGAGGGAGACCCTGTCTCAAAAACAAATTATAATAAAATAATATGTATTTCAAAATATGGATGTACTGAGGCAGAAGAC
ACAATGAGGCCGAGCGTGGTGGCTTATGCCTGTAGTCCCAGCACTTTGGGAGATCCACAAAGTGGGTGGATCACCTGAGGTCAGGAG
TTCAAGACCAGCCTGGCCAACATGGCGAAATCCCATCTCTACTAAAAATACAAAAATTAGCCTGGTGTGGTGGCAAGTGCCTGTAATC
CCAGCTACTCAGGAGGCTGAGGCAGGAGAATCGCTTGAACCTGGGAGGGAGAGGTTGTGGTGAGCTGAGATCGAGCCACTGCACTCC
AGCCTCAGCAACAGAATGAGACTGTGTCTGAAAACAAAAACAAAAACACAAAAAGCTGAACTTCTGCTTGTGTTTCTCCCTTGCCTGA
GCCGGAATTGAATTTAGCCTTTGTTTTCCCAGATTTGGATGATGTATGAGTTCCTTAACACCCAGGAAATGAAAGTGAAGACAGGATA
CTTGAATGAAAACAGGATTGGGCCAATAATAACAGACCAGGTGATCAGAGAAAGAGGAAAATAATCTTAAATTAAGCAAAGATTGG
CCTAGTGCGGTAGCTCATGCCTGTAATTCCAGGGCTCTGGGAGGGTAAGCTGGGAGGATTGCTTGAAGCTGGGAGTTCAAGGCCAGCT
TGGGCAATAGAGCAAGACTCCATCCTTTTTTTGTTGGTTTGTTTGTTTTTGTTTTGAGACAGAGTCTCACTCTGTCACCAGGCTGGAGTG
CAGTGGTATGATCTCGGCTCACTGCAACCTCCGCCTCCTGGGTTCAAGTGATTCTCCTGCCTCAGCCTCCTGAGTAGCTGGGATTACAG
GCTTGTGCCACCACGCCCGGCTAATTTTTATATTTTTAGTAGAGACGGGGTTTCACCACGTTGGCCAGGCTGGTCTCGAACTCCTGACC
TCAGGTGATCTGCCCGCCTCGGCCTCCCAAAGTGCTGGGATTACAGGCGTGAGCCACCTGGCCAGTATCTTTTTTATTTGTTTGCTTTA
CCATTATTTGTCCCCACTGGCATGCAACCGTTGGTTTAGTTTAGTTAATGGTTAAAGGAGTAGGCTCTGGAACAGGGCTTCTCAGGTCT
AAAGCTTGGTTCTGCCCCCTAGTTAGCTGTGTGCTCTTAGACAAGCTTAGTCTGCATGTTTGTTTACTCGTCTATACAGTGGGATTAAT
GGCACCTAGGCTGGATAAGGTGAGGCACACCTGCAATCCCACTGTTTTGAAAGGCTGAGGTGGAAGGATCACTTGAGGTCAGGAGTT
TGAGGCTGCGGTGAGCTATGATCGTGCCACTGCACTCGAAAAAGAAAAAGAAAAGAAGGTTGGGGCCGGGCACGGTAGCTCACACCT
GTAATCCCAGCACTTTGGGAGGCCGAGGTGGGTGGATCACGAGATCAGGAGTTCGAGACCAGCCTAGCTAAAATGGTGAAACCCCGT
CTCTACTAAAAATACAAAAATTACCTAGGCGTGGTGGTGTGTGCCTGTAATCCCAGCTACTCAGCAGGCTGAGGCAGTAGAATCGCTT
GAACCCAGGAGGCAGAGGTTGCGGTGAGCTAAGATCGCGCCACTGCACTCCAGCCTGGACGACAGAGTGAGACCCGGTCTCAAAAA
AAAAAAAAAATGCTTAGCGCAGTGCCTAGAAGATAATCAGTGTGCAATAAATGCTTGCTATTTTAATTATCATTATTCTCGGGGCAGG
AATCTTG
