# Supplementary material for: NUPR1, a new target in liver cancer: implication in controlling cell growth, migration, invasion and sorafenib resistance
Source: Cell Death Dis. 2016 Jun 23;7(6):e2269–. doi: 10.1038/cddis.2016.175 (PMC5143401; doi:10.1038/cddis.2016.175)
Supplement: Supplementary Figure Legends [file cddis2016175x14.doc]

| **Supplementary Table 1.** Dataset details | | | | | | | | |
| --- | --- | --- | --- | --- | --- | --- | --- | --- |
|  | **Dataset name** | **Normal** | **HCC** | **Cancer precursor** | **Clinical**  **information** | **Platform** | **Dataset source** |  |
|  | *Archer KJ *et al*. (23) | 0 | 16 | 47 | No | U133 Plus 2 | ONCOMINE |  |
|  | *Chen X *et al*. (24) | 76 | 104 | 7 | No | custom | ONCOMINE |  |
|  | *Chiang DY *et al.* (25) | 0 | 91 | 0 | Yes | U133 Plus 2 | ONCOMINE |  |
|  | Jia HL *et al.* (26) | 0 | 283 | 0 | Yes | custom | ONCOMINE |  |
|  | Liao YL *et al.* (27) | 2 | 11 | 0 | Yes | U133 Plus 2 | ONCOMINE |  |
|  | *Mas VR E *et al*. (28) | 19 | 38 | 58 | No | U133A 2.0 | ONCOMINE |  |
|  | Roessler S *et al.* (29) | 21 | 22 | 0 | No | HT U133A | ONCOMINE |  |
|  | *Roessler S *et al*. (29) | 220 | 225 | 0 | No | U133A 2.0 | ONCOMINE |  |
|  | *Wurmbach E *et al*. (30) | 10 | 35 | 30 | Yes | U133 Plus 2 | ONCOMINE |  |
|  |  |  |  |  |  |  |  |  |

*Datasets that show differential expression analysis with a statistic significance less than 0.05 (by t-test).
